# Supplementary material for: Prevalence of domestic violence against women in informal settlements in Mumbai, India: a cross-sectional survey
Source: BMJ Open. 2020 Dec 16;10(12):e042444. doi: 10.1136/bmjopen-2020-042444 (PMC7745686; doi:10.1136/bmjopen-2020-042444)
Supplement: Author's manuscript [file bmjopen-2020-042444.draft_revisions.pdf]

BMJ Open

BMJ Open is committed to open peer review. As part of this commitment we make the peer review history of every article we publish publicly available.

When an article is published we post the peer reviewers' comments and the authors' responses online. We also post the versions of the paper that were used during peer review. These are the versions that the peer review comments apply to.

The versions of the paper that follow are the versions that were submitted during the peer review process. They are not the versions of record or the final published versions. They should not be cited or distributed as the published version of this manuscript.

BMJ Open is an open access journal and the full, final, typeset and author-corrected version of record of the manuscript is available on our site with no access controls, subscription charges or pay-per-view fees (http://bmjopen.bmj.com).

If you have any questions on BMJ Open's open peer review process please email info.bmjopen@bmj.com

BMJ Open

Prevalence of domestic violence against women in informal settlements in Mumbai, India: a cross-sectional survey

Journal:	*BMJ Open*
Manuscript ID	bmjopen-2020-042444
Article Type:	Original research
Date Submitted by the Author:	06-Jul-2020
Complete List of Authors:	Daruwalla, Nayreen; Society for Nutrition, Education & Health Action (SNEHA)<br>Kanougiya, Suman; Society for Nutrition, Education & Health Action (SNEHA)<br>Gupta, Apoorwa; Society for Nutrition, Education & Health Action (SNEHA)<br>Gram, Lu; University College London, Institute for Global Health<br>Osrin, David; University College London, Institute for Global Health
Keywords:	PUBLIC HEALTH, PRIMARY CARE, EPIDEMIOLOGY

SCHOLARONE™
Manuscripts

I, the Submitting Author has the right to grant and does grant on behalf of all authors of the Work (as defined in the below author licence), an exclusive licence and/or a non-exclusive licence for contributions from authors who are: i) UK Crown employees; ii) where BMJ has agreed a CC-BY licence shall apply, and/or iii) in accordance with the terms applicable for US Federal Government officers or employees acting as part of their official duties; on a worldwide, perpetual, irrevocable, royalty-free basis to BMJ Publishing Group Ltd ("BMJ") its licensees and where the relevant Journal is co-owned by BMJ to the co-owners of the Journal, to publish the Work in this journal and any other BMJ products and to exploit all rights, as set out in our licence.

The Submitting Author accepts and understands that any supply made under these terms is made by BMJ to the Submitting Author unless you are acting as an employee on behalf of your employer or a postgraduate student of an affiliated institution which is paying any applicable article publishing charge ("APC") for Open Access articles. Where the Submitting Author wishes to make the Work available on an Open Access basis (and intends to pay the relevant APC), the terms of reuse of such Open Access shall be governed by a Creative Commons licence – details of these licences and which Creative Commons licence will apply to this Work are set out in our licence referred to above.

Other than as permitted in any relevant BMJ Author's Self Archiving Policies, I confirm this Work has not been accepted for publication elsewhere, is not being considered for publication elsewhere and does not duplicate material already published. I confirm all authors consent to publication of this Work and authorise the granting of this licence.

For peer review only

Prevalence of domestic violence against women in informal settlements in Mumbai, India: a cross-sectional survey

Authors

Nayreen Daruwalla

Program on Prevention of Violence Against Women and Children, SNEHA, Mumbai, Maharashtra, 400017, India

nayreen@snehamumbai.org

Suman Kanougiya

Program on Prevention of Violence Against Women and Children, SNEHA, Mumbai, Maharashtra, 400017, India

suman.kanougiya@gmail.com

Apoorwa Gupta

Program on Prevention of Violence Against Women and Children, SNEHA, Mumbai, Maharashtra, 400017, India

apoorwa.gupta@snehamumbai.org

Lu Gram

Institute for Global Health, University College London, London, WC1N IEH, UK

lu.gram.13@ucl.ac.uk

David Osrin (corresponding author)

Institute for Global Health, University College London, London, WC1N IEH, UK

d.osrin@ucl.ac.uk

Contributorship

ND and DO conceived the study and acquired funding. ND, AG, SK, LG, and DO developed the methodology, ND, SK, and AG oversaw investigation, and SK and DO curated the data. DO did the first analysis and visualization and wrote the first draft. ND managed the project with support from SK. All authors critically reviewed drafts of the manuscript and read and commented on the final version.

Data sharing

Data are available in Open Science Framework: *Osrin, D. (2020, June 30). Prevalence survey. Retrieved from osf.io/khdcm*

For peer review only

Abstract

Introduction

Domestic violence against women harms individuals, families, communities, and society. Perpetrated by intimate partners or other family members, its overlapping forms include physical, sexual, emotional, and economic violence, coercive control and neglect. We aimed to describe the prevalence of these forms of violence and their perpetrators in informal settlements in Mumbai.

Methods

We did a cross-sectional survey with 100 women aged 15-49 years in each of 50 clusters in informal settlements, including questions on general health and wellbeing, household control and neglect, experience of economic, emotional, physical, and sexual violence, and disclosure. We present responses to individual questions and composite indicators.

Results

The survey included 5122 respondents, of whom 644 (13%) had experienced physical domestic violence in the last year, 188 (4%) sexual violence, and 963 (19%) emotional violence. 13% of ever-married women had experienced physical or sexual intimate partner violence in the last year. Most physical (87%) and sexual violence (99%) was done by partners, but emotional violence equally involved marital family members. All three forms of violence were more common if women were younger, in the lowest socioeconomic asset quintile, or reported disability. 1816 women (35%) had experienced at least one instance of coercive control and 33% said that they were afraid of people in their home. 10% reported domestic neglect of their food, sleep, health, or children's health.

Conclusion

Domestic violence against women remains common in urban informal settlements. Physical and sexual violence was perpetrated mainly by intimate partners, but emotional violence was attributed equally to partners and marital family. More than one-third of women described controlling behaviours perpetrated by both intimate partners and marital family members. We emphasise the need to include the spectrum of perpetrators and forms of domestic violence – particularly emotional violence and coercive control - in data gathering exercises.

Key words

Domestic Violence; Gender-based Violence; Intimate Partner Violence; Mumbai; India

Funding

Wellcome Trust 206417

For peer review only

Article summary

Strengths and limitations of this study

- In a large cross-sectional survey of 5122 women in informal settlements, we found that 13% had suffered physical and 4% sexual domestic violence in the last year, mostly from their intimate partners.
- However, 19% had suffered emotional violence, 35% had been subjected to controlling behavior, and 10% had suffered neglect of their food, sleep, health, or children's health.
- These forms of domestic violence were perpetrated by both intimate partners and in-laws.
- The full spectrum of domestic violence—particularly emotional violence and coercive control—and the potential range of perpetrators are sources of concern.
- Limitations include the cross-sectional nature of the study, the possibility of under-reporting, and an emphasis on younger married women.

For peer review only

For peer review only - http://bmjopen.bmj.com/site/about/guidelines.xhtml

Background

The global burden of domestic violence is underlined by its inclusion as an indicator of gender equality for the fifth United Nations Sustainable Development Goal (https://sdg-tracker.org/gender-equality). Domestic violence against women harms individuals, families, communities, and society. For individuals, it causes injury or death (1, 2), reproductive health problems, harmful drug and alcohol use, anxiety, depression, post-traumatic stress disorder, self-harm, and suicide (1, 3). For families, it leads to miscarriage, induced abortion, stillbirth, low birth weight, preterm delivery, and further violence (4, 5). For communities and society, it leads to lack of agency, limited participation, and lost economic productivity (5, 6).

Globally, 30% of women have survived physical or sexual violence by an intimate partner or sexual violence by a non-partner (4). The precision of the wording of this statement illustrates the challenge of defining violence against women. The United Nations Declaration on the Elimination of Violence against Women defines it as ‘‘any act of gender-based violence that results in, or is likely to result in, physical, sexual or psychological harm or suffering to women, including threats of such acts, coercion or arbitrary deprivation of liberty, whether occurring in public or in private life” (7). This definition allows for a range of perpetrators and a range of forms of violence. The perpetrator may be a husband or partner (intimate partner violence), a family or household member, or someone known or unknown outside the domestic environment (in this paper, we define domestic violence as falling into either or both of the first two categories). Figure 1 conceptualises the intersecting forms of domestic violence: physical, sexual, emotional or psychological, control, neglect, or economic. These forms of violence overlap and occur in combination, in some settings with forms such as female genital mutilation and modern slavery.

There is consensus that measuring the scope and prevalence of violence against women is important, and that we should try to measure its different sources and forms (8). Doing so may help identify the harm it can cause, advocate on behalf of individual women and against an unacceptable societal burden of coercive control (a perspective that owes much to the work of feminists over the last half century), and compare research findings across time and place and in a shared language (9). We should be aware, however, that an emphasis on definition and metrics is characteristic of a public health approach (10). As feminists have emphasized, violence against women is equally a human rights and criminal justice concern (11), the product of an ecology of micro, meso, and macro factors (12), and a manifestation of structural violence and gendered power imbalances. Too tight a focus on its epidemiology might depoliticize violence against women and shift attention away from these issues (13).

Surveys have been important in international efforts to establish prevalence, the most prominent being the WHO multi-country study on women’s health and domestic violence against women (14) and the International Violence against Women Surveys (15). Choosing and comparing between the available scales and questionnaires is difficult (16, 17). The challenges to measurement are substantial (18), not least in terms of definitions and terminology (19). Early surveys focused on intimate partner physical violence. The Conflict Tactics Scale (20), on which they have often been based, has been extended to include sexual and emotional violence (21), and the definition has been extended to include perpetrators other than intimate partners (22). Whatever has been achieved in terms of definitions and scales, a fundamental challenge remains disclosure: women may not perceive, acknowledge, or communicate that they are surviving violence (23, 24). This is a central issue for clinical and social service provision (25-29), makes crime and hospital records difficult to use in estimating prevalence, and extends to interviews in surveys.

India is a signatory to the Convention on the Elimination of All Forms of Discrimination Against Women (30). Violence against women is addressed by criminal law, particularly domestic violence in Section 498-A of the Indian Penal Code, and by civil law in the form of the Protection of Women from Domestic Violence Act, 2005, which encompasses the varied forms of domestic violence and its perpetrators (available at

For peer review only - http://bmjopen.bmj.com/site/about/guidelines.xhtml

https://indiacode.nic.in/bitstream/123456789/2021/1/200543.pdf). A systematic review of 137 studies suggested that 22% of women in India had survived physical violence in the past year (131 studies), 22% had suffered emotional or verbal psychological violence (60 studies), 7% sexual violence (79 studies), and 30% multiple forms of violence (31). Women in India also commonly experience forms of violence that extend to coercive control and neglect (32), articulated in the idea of gender-based household maltreatment (33), and captured in the recent Indian Family Violence and Control Scale (34).

We work on primary, secondary, and tertiary prevention of violence against women in informal settlements in Mumbai. The prevalence of physical and emotional violence in such settings has been reported as high (35-38). At the beginning of a programme of community mobilisation to prevent violence against women and girls, we conducted a baseline survey. Our objectives in this analysis were to estimate the prevalence of physical, sexual, and emotional violence, control and neglect. We were particularly interested in sources of domestic violence other than intimate partners and in forms of violence other than physical.

Methods

Setting

Over 377 million people live in urban India (39, 40). Around 40% of Mumbai's homes are in informal settlements (41), characterized by overcrowding, insubstantial housing, insufficient water and sanitation, lack of tenure, and hazardous location (42, 43). SNEHA (Society for Nutrition, Education and Health Action) is a non-government organization working to improve health in such settlements. The SNEHA programme on Prevention of Violence against Women and Children began in 2000. Primary prevention is addressed through a combination of community group activities and resulting individual voluntarism. Secondary prevention includes local crisis response and psychological first aid by community organisers and referral to centres which provide counselling, legal, and psychotherapeutic support, with links to the police and medical, shelter, and social service providers. Tertiary prevention is provided primarily through referral to psychiatric and legal services. Having satisfied criteria for counselling, shelter, legal aid, and access to medical care, SNEHA is a service provider under the Protection of Women from Domestic Violence Act, 2005, and is authorised to file Domestic Incident Reports. Under the Protection of Children from Sexual Offences Act, 2012, SNEHA has reporting rights to the police and Child Welfare Committee.

Design

We did a cross-sectional survey of women's experience of violence, collected at the beginning of a cluster randomized controlled trial of community interventions to prevent violence against women and girls (the SNEHA TARA trial) (44). An estimate of prevalence from a cross-sectional sample of 5000 in a population of 125,000 would have a precision of ~1%. Within this, a comparison of two categories of determinant for 100 respondents in each of 50 clusters would provide 80% power to detect a difference of 6% in prevalence estimates of 10-20%.

Data collection

Data were collected from clusters of around 500 households. Potential clusters were identified in four phases in two areas of the city, and 50 were selected on the basis of (a) vulnerability assessed against a scorecard (45), (b) no known plans for rehabilitation or demolition in the next two years, (c) absence of existing nongovernment organization programs addressing violence against women, (d) more than 75% of structures residential and less than 25% rental, and (e) clear separation from each other. Structures and homes within clusters were mapped and 16 women investigators with graduate education and three months training visited households to enumerate residents and list possible participants. We aimed to complete 100 questionnaires in each cluster (5000 in total). Investigators began at a random start point and visited every second household to enroll participants. Inclusion criteria were that respondents should be women aged 18-49 years who were usual

For peer review only - http://bmjopen.bmj.com/site/about/guidelines.xhtml

residents. When there was more than one potential respondent in a household, the investigator applied an algorithm that selected the youngest woman at risk of disability, followed by the youngest married woman, followed by the youngest unmarried woman.

The questionnaire included modules on general health and wellbeing, common mental disorder, household decision-making, household power and control, neglect, experience of economic, emotional, physical, and sexual violence, disclosure and support. Questions were taken from existing Hindi versions where possible. If not, they were translated from English, piloted in two clusters external to the trial, amended, and back-translated.

Data management

Investigators used electronic tablets to enter information in a database in CommCare (www.dimagi.com). To optimize accuracy, the system included field constraints, lookup tables, and automated skip logic.

Statistical analysis

In developing our questionnaire, we aimed to provide data that would allow comparisons between studies and settings. We included questions from major surveys, international and national, and it should be possible to develop summary measures of violence against women using any of a number of combinations of questions. Our idea was to make the resulting anonymized dataset available so that others might use it, choosing specific questions to facilitate comparison or pooling of data. Here we present composite indicators of lifetime and 12-month prevalence of physical, sexual, and emotional violence.

Table 1 summarises the questions in three surveys from India (our TARA Trial questionnaire, the National Family Health Survey (NFHS-4) (50), and the Indian Family Violence and Control Scale (34), and two international surveys (the International Violence Against Women Survey (15) and the WHO multi-country study (14)).

Our composite estimate of prevalence of physical violence included nine questions that were comparable across surveys, although there was variation in whether threats of violence were considered incidents of physical violence. The estimate of prevalence of domestic sexual violence included four questions. The comparable questions about sexual violence were similar, although only the TARA survey and the Indian Family Violence Survey included a question on coercion to replicate activities seen in pornographic material. The estimate of prevalence of domestic emotional violence included five questions that were comparable across surveys. We asked a series of questions about domestic coercive control, addressing mobility, education, employment, appearance, and pregnancy. For control and neglect, we present the individual questions because composite indicators are not yet established.

We used the Washington Group Short Set on Functioning questions to identify disability, including difficulty with seeing, hearing, walking, remembering, self-care, and speaking (http://www.washingtongroup-disability.com/washington-group-question-sets/short-set-of-disability-questions/). We took the most permissive of four possible approaches to classification, in which disability is registered if the respondent reports at least some difficulty in at least one of the six domains (51). We classified socioeconomic position with quintiles of scores derived from standardized weights for the first component of a principal components analysis of 22 individual household assets (52, 53).

Prevalence is summarized by frequency and percentage. We plotted prevalence of physical, sexual, and emotional violence in the last year by age group, schooling, socioeconomic asset quintile, and disability, collapsed by grouping variable and rounded using the twoway (connected) command in Stata 15. We calculated odds ratios for associations using logistic regression and Stata survey commands with cluster as the primary sampling unit and phase as stratum. We calculated intracluster correlation coefficients (ICC) for prevalence of physical, sexual, and emotional violence in the last year using the loneway command in

For peer review only - http://bmjopen.bmj.com/site/about/guidelines.xhtml

Stata 15. The paper was prepared according to the STROBE Statement for reports of observational studies (www.strobe-statement.org).

Ethical considerations

We took permission for the survey from cluster gatekeepers identified by residents (46, 47), and followed WHO guidelines for research on domestic violence against women (48), and on sexual violence (49). Participants gave signed consent after discussing a participant information sheet which they were welcome to keep or return. Data collectors explained their right to not provide answers to specific questions, to terminate the interview, and to withdraw temporarily or permanently without penalty. All team members were trained in Good Clinical Practice for research ethics and participant protection (Scientia Clinical Services, 14th February 2019), and interviewers were supported by three field supervisors with direct linkage to counselling services. They were all women, ensured privacy for interviews, and provided both time and sufficient information for women to consider whether to participate. Interviews began with general questions about demography, household residents, education, socioeconomic position, maternity, and health.

Interviewing women about their possible experience of violence raises issues of consent, interviewer behaviour, privacy, and confidentiality. Of particular concern is the duty of care after disclosure. We believe that an interviewee who discloses experience of violence should be offered optimal support, and interviewers were members of a broader team who were able to provide a full suite of crisis and counselling services, including home visits, medical, surgical, and psychiatric referral, and negotiation with families, the police, and legal representatives. When survivors disclosed violence, we followed established intervention protocols which included safety assessment, counselling, liaison with healthcare, police, and legal services, and developing follow-up plans for the survivor and her family. When a survivor was not ready to disclose violence, the interviewer provided her with information on available services and legal rights. She took consent for any action from the participant herself.

The trial and associated data collection were approved by the Institutional Ethics Committee of Partners for Urban Knowledge, Action, and Research (PUKAR) (25th December 2017), and the University College London Research Ethics Committee (3546/003, 27th September 2017). The TARA trial within which data collection took place is registered with the Controlled Trials Registry of India (CTRI/2018/02/012047, 21st February 2018) and with ISRCTN84502355 (22nd February 2018: http://www.isrctn.com/ISRCTN84502355).

Patient and public involvement

Community members have been involved in the design of our research since 2000. It arises from the need to provide and improve services for survivors of violence, and a commitment to working with communities to prevent it. Research questions and outcome measures were derived from international efforts, but client priorities were reflected in some specific questions that we asked as a result of client experiences. All questions were discussed with participants in pilot exercises in order to understand their perceptions and ability to respond. Community guardians were involved in recruitment and the practicalities of administering the survey. Respondents took about an hour to answer the questions, and participated only after consideration. The general findings will be disseminated through community groups involved in ongoing interventions in the study areas.

Results

Participants

Between 5th December 2017 and 28th March 2019, 5277 households were approached for the survey. 423 (2%) had no eligible woman resident and in 544 (3%) an eligible woman was unavailable after three visits. Interviewers were unable to achieve privacy in 592 cases

For peer review only - http://bmjopen.bmj.com/site/about/guidelines.xhtml

(11%) and 155 (3%) potential respondents declined interview. The final survey included 5122 respondents.

Table 2 summarises characteristics of households, respondents, and their partners. Dwellings were generally of robust or mixed robust and insubstantial construction (93%). Only 17% had their own toilet. Respondents were predominantly of Hindu (59%) or Muslim (37%) faith and 58% described themselves as of general caste. 68% of women had been born outside Mumbai, 96% had been married or partnered, 2% had separated or divorced, and 2% had been widowed. 36% had married before the age of 18 years, 88% of them by arrangement, 14% had had a pregnancy before the age of 18 years, and 36% had three children or more. Women were a mean 32 years old (SD 7.3) and their partners a mean 36 (SD 8.1). 18% of women had had no schooling, compared with 10% of partners, and 36% had completed high school, compared with 48% of partners. Only 24% of women had undertaken paid work in the last year, compared with 98% of men. For these women, the main types of employment were home-based (62%), informal or formal services (17%), and domestic labour (10%). The main types of employment for men were in informal or formal services (49%) or as drivers (18%).

Domestic physical violence

Table 3 presents lifetime and 12-month prevalence of domestic violence. It includes individual questions and composite estimates for physical (9 questions), sexual (4 questions), and emotional violence (5 questions). 25% of women had ever experienced domestic physical violence and 13% had experienced it in the last year. This was usually intimate partner violence (11%), but 2% had experienced physical violence from their marital family and 2% from their natal family. Around 4% of women had been kicked, dragged, or beaten in the last year, and around 3% had been threatened or attacked with a blunt or sharp object.

Domestic sexual violence

6% of women had ever experienced domestic sexual violence and 4% had experienced it in the last year, almost all by their intimate partner. Removing the question about coercion to replicate pornography from the composite estimate reduced the estimated prevalence of sexual violence by 0.1% (domestic sexual violence to 5.4% ever (274 reports) and 3.5% in the last year (179), and intimate partner sexual violence to 5.1% ever (262) and 3.5% in the last year (177). Additional questions suggested that 2.5% of partners had insisted on repeated intercourse (126 women in the last year), that 1.1% of wives had been forced to view pornography (56), that 0.8% had been subjected to coercive sex without contraception (39), and that 1.0% of partners were reported as withholding sexual pleasure from their wives (49). The prevalence of domestic physical or sexual violence in the last 12 months was 14% for currently married women, but 29% for women who had separated from their partner.

Domestic emotional violence

19% of women had experienced emotional violence in the last year. The source was roughly evenly split between intimate partner (12%) and marital family members (11%). Emotional violence came from both intimate partner and marital family for 5% of women. 6% of women had been accused of infidelity, 6% had been insulted for being a woman, and 8% of 505 women with disability had been insulted for it.

Summary figures

Limiting the dataset to 4913 ever-married women for comparison with surveys such as the NFHS, the prevalence of domestic physical violence in the last 12 months was 13%, of sexual violence 4%, of physical or sexual violence 14%, and of emotional violence 19%. Recent guidance suggests that studies present the proportion of ever-partnered women and girls aged 15 years and older who have survived physical, sexual, or psychological violence

For peer review only - http://bmjopen.bmj.com/site/about/guidelines.xhtml

252 by an intimate partner in the last year (54). For intimate partner violence (a subgroup within domestic violence) our findings were 11% for physical violence, 4% for sexual, 13% for emotional (psychological), 13% for physical or sexual, and 17% for physical, sexual, or emotional violence.

256 Figure 2 shows prevalence of physical, sexual, and emotional violence for each of 50 clusters. The ranges were substantial: for physical violence 2-27% with interquartile range (IQR) 12-16%, for sexual violence 0-8% (IQR 2-5%), and for emotional violence 7-32% (IQR 15-23%). Intracluster correlation coefficients (ICC) were 0.023 for domestic physical violence in the last year (95% CI 0.011, 0.036), 0.005 for sexual violence (0.000, 0.010), and 0.016 for emotional violence ICC (0.006, 0.026).

262 Figure 3 summarises prevalence of physical, sexual, and emotional violence by age group, schooling, socioeconomic asset quintile, and disability. Women were less likely to face physical and emotional violence as they aged: the prevalence of physical violence halved between the twenties and the forties. There was no obvious pattern for schooling. All three forms of violence were more common against women in the lowest socioeconomic asset quintile and against women with disability. The odds ratios in Figure 3 did not change substantially when all four covariates were included in multivariable models, apart from for disability, which was associated with an adjusted odds ratio of 1.68 (95% confidence interval 1.24, 2.29) for physical violence, 2.18 (1.37, 3.47) for sexual violence, and 1.66 (1.31, 2.12) for emotional violence.

272 *Domestic coercive control*

273 **Table 4** summarises forms and sources of coercive control. We took a conservative approach to classifying controlling behaviour. A restrictive behaviour was registered only if it was reported as always occurring, and a permissive behaviour only if it was reported as never occurring. Overall, 1816 women reported being subject to at least one controlling behavior in the list in Table 4 (35%), although we recorded responses to a larger set of questions. Respondents commonly reported that their mobility and socializing were restricted: 26% said that they always required permission to go out, 14% that they were never allowed out in the evening, and 10% that they were always accompanied when they did go out. 48% were never allowed to meet with male friends, 10% were never allowed to meet with female friends, and 5% were never allowed to meet their natal family. At home, 33% of women said that they were afraid of family members. 13% were never free to speak on the phone and 10% were never able to speak freely in the home. These kinds of restrictions were described as coming more often from marital family members than from partners. In terms of sexual and reproductive health, 52 women had been denied access to contraception and 15 had been coerced to use it. 37 (1% of 4577) had been prevented from having a termination of pregnancy, while 15 (0.3%) had been coerced to have one.

289 *Domestic neglect*

290 7% of women reported that their family had neglected their food in the last year, 8% that they had neglected their sleep, 8% that they had neglected their health, and 12% of 4375 with children that they had neglected their children's health. Collectively, 10% of women reported at least one of these four dimensions of neglect.

294 *Disclosure of violence*

295 Of 1153 women who had survived physical, sexual, or emotional domestic violence in the last year, 47% had disclosed to someone. The main confidants were family and friends (40%) and teachers, faith leaders, or local leaders (4%). Even though 18% had sought medical help, only 8% had disclosed to a healthcare provider, social worker, or counsellor. Twenty women approached our services for support within two months of the survey.

300 Discussion

For peer review only - http://bmjopen.bmj.com/site/about/guidelines.xhtml

Our cross-sectional survey of 5122 women aged 15-49 years in informal settlements in Mumbai documented high rates of domestic violence. In the preceding year, 13% of women had suffered physical violence, 4% sexual violence, and 19% emotional violence. Physical and sexual violence was perpetrated mainly by intimate partners, but emotional violence was perpetrated equally by intimate partners and marital family members. Controlling behaviours were ascribed to both intimate partners and marital family members, with 35% of women reporting at least one of 12 restrictions.

Our findings have three general limitations. First, although the prevalence of domestic violence was high, we should assume some underreporting given the constellation of forces inhibiting disclosure. Second, we preferentially recruited younger, married, and disabled women within households, which would tend to increase reported prevalence. Third, our study was cross-sectional and subject to the usual caveats around causal inference and the accuracy of time recall.

We updated the review of quantitative studies by Kalokhe and colleagues (31), identifying publications from 1st January 2015 to 1st November 2019 using similar search terms, and found eight more recent published studies. The reported prevalence of domestic violence was higher in rural (55-57) than in urban settings. The estimated lifetime prevalence of physical violence ranged from 18% (58) to 27% (59, 60), of sexual violence from 10% (58) to 26% (60), and of emotional violence from 20% (58) to 43% (this high estimate included questions on economic violence)(60). A cross-sectional survey similar to ours, of 1137 mothers aged 18-39 years in an informal settlement in Mumbai, found a lifetime prevalence of physical intimate partner violence of 17%, of emotional violence of 12%, and of sexual violence of 5% (61).

Our findings also compare with the NFHS-4 for Maharashtra state, in which estimates of prevalence of intimate partner violence were based on interviews with 2472 ever-married women aged 15-49 years (62). Both surveys estimated lifetime physical violence at 21%. The NFHS-4 estimate for the last 12 months was 15% (our study 11%). We found higher prevalence of sexual (5% ever compared with 2% in the NFHS-4; 4% in the last 12 months compared with 2%) and emotional violence (17% ever compared with 10% in the NFHS-4; 12% in the last 12 months compared with 7%). Our estimates of prevalence of all three types of violence were higher than those of the urban sample in the Maharashtra NFHS-4, which interviewed 1220 women (lifetime intimate partner physical violence 21% in our study, compared with 16% in the NFHS-4, sexual violence 5% compared with 2%, and emotional violence 17% compared with 9%). There are three possible explanations for our higher estimates. First, they might be based on different sets of questions: we used similar questions and composites of them and do not think this was an issue. Second, they might reflect true population differences: our participants were all residents of informal settlements and might be more likely to face violence than the urban average. Third, women might have been more likely to disclose violence to our team of interviewers, who were backed up by a strong programme of support for survivors. It is also possible that women answer questions differently when they are responding to a survey on violence than when the questions on violence are included in a broad survey like the NFHS-4 (10).

We have a clear picture of the high burden of domestic violence against women, and of the range of findings across urban and rural settings in India. The odds of physical violence are greater for younger married women, with poorer socioeconomic position, and where underage marriage is common (63). We found that 47% of women who had suffered violence had sought help from somebody. This is considerably more than the NFHS-4, which found that 14% of urban women who had experienced physical or sexual violence had sought help from any source, usually natal family, marital family, friend or neighbor. Although disclosure to a healthcare provider, social worker, or counsellor was uncommon (8%), it was also higher than the NFHS-4 urban figure of 3%. Perhaps this provides some hope. Awareness of domestic violence is certainly increasing and it may be that women are

For peer review only - http://bmjopen.bmj.com/site/about/guidelines.xhtml

becoming more confident that what they are going through is violence and that it is not acceptable.

Our particular concerns are perpetration of violence by the marital family and the burden of emotional violence and coercive control. Although only 11% of studies in the recent review considered non-partner violence (31), domestic violence by people other than intimate partners has long been a concern in the Indian context (64). Women entering the marital household are traditionally subordinate and the spectrum of violence extends from subtle neglect to dowry death. Toleration of their subservience in exchange for security and later authority over their own daughters-in-law has been described as the patriarchal bargain (65), and the burden of violence against women by mothers-in-law (66) and other family members has been termed gender-based household maltreatment. It informed the design of our own questionnaire (67) and includes control over reproduction, limiting contact with friends and family, and access to food (32, 34). These kinds of less visible emotional violence and control have been less well studied, but they need to be: marital family members were the source of emotional violence in about half of reports in our study and of 35% of domestic violence in rural Tamil Nadu (55).

Recent work on conceptualising and measuring psychological violence (here termed emotional violence) has suggested that it is distinct from controlling behaviour (68). More than one-third of women in our study reported experiencing one of 12 controlling behaviors and other studies have reported high levels of control by intimate partners: 72% in rural Tamil Nadu (56), and 60% in rural Rajasthan (57), 43% in urban Delhi (58), and 12% in Karnataka (69). In a survey of married women in Uttar Pradesh, 12% had experienced reproductive control from husbands or in-laws, with a roughly equal split between them (70).

Although emotional violence and coercive control are less visible than physical violence, the gravity of their effects on women's mental health is being increasingly appreciated (71). Follingstad identified six types of emotional abuse that survivors of violence in the United States had experienced: verbal attacks (ridicule, name-calling, humiliation in public), isolation (social or financial), jealousy and possessiveness (including accusations of infidelity), threats of harm, threats of divorce or abandonment, and destruction of personal property. All had strong negative effects on women and 73% said that emotional violence had greater impact than physical violence (72). This finding is supported by both qualitative (72, 73) and quantitative research in high-income countries (74). In an analysis of the US National Violence Against Women Survey, psychological violence was more strongly associated with depressive symptoms than physical violence, and abuse of power and control were more strongly associated with depressive symptoms than verbal abuse (75).

We are seeing similar evidence from India. In rural Rajasthan, Richardson and colleagues "… found evidence that psychological abuse and controlling behavior were more damaging to mental health than physical abuse" (57). A cross-sectional survey of 9938 mothers aged 15-49 years in seven urban and rural locations, of whom 3155 lived in urban informal settlements, suggested that severe harassment by in-laws was associated with poor mental health (76). An analysis of follow-up data from 6303 rural married women aged 15-49 years in Bihar, Jharkhand, Maharashtra, and Tamil Nadu showed a strong association between 'verbal' violence and mental health and a much weaker association with physical violence (77).

While domestic violence is a global experience, there are differences in cultural experiences. A feature of the discussion in high-income countries from the 1970s onward was the disappointment of professional providers of support when a survivor of intimate partner violence did not leave the perpetrator (23, 24, 78). The emphasis in India – on the part of survivors and professionals – has been on interventions that allow families to remain together. Tolerating domestic violence has been a normalized element of women's reproductive labour (79, 80), and the ability to bear it and still fulfil the expectations of a wife and mother has been seen as praiseworthy (81, 82) and characteristic of the 'real' or 'good'

For peer review only - http://bmjopen.bmj.com/site/about/guidelines.xhtml

woman (83). Even when domestic violence spills out into community knowledge, women who break with this tradition risk social ostracism (84), and their perceptions of their options are limited (27, 66)

Conclusion

Domestic violence against women continues to be common in urban informal settlements in Mumbai and we see no reason to doubt the external validity of our findings for other locations. Physical and sexual violence is perpetrated mainly by intimate partners, but emotional violence against women is attributed equally to partners and marital family. More than one-third of women describe at least one of a limited set of controlling behaviours, perpetrated more by intimate partners, but often by marital family members. We emphasise the need to include the spectrum of domestic violence – particularly emotional violence and coercive control - and perpetrators in data gathering exercises.

Funding statement

The funder had no role in study design, data collection and analysis, decision to publish, or preparation of the manuscript.

Acknowledgments

Our greatest thanks go to the women and community guardians who agreed to contribute to the study. We thank the team of investigators who collected data in challenging conditions, Miheeka Vast and Manju Singh for supervising the field investigation team, Unnati Machchhar and Shilpa Adelkar for their supervision of the intervention programme, Gauri Savkur for contributing to investigator training, Bhaskar Kakad for investigator support, Archana Bagra and Vibhavari Bali for financial and human resources management, and Vanessa D'Souza and Shanti Pantvaidya for leadership at SNEHA.

List of figures and tables

Figure 1. Forms of domestic violence against women

Figure 2. Prevalence of physical, sexual, and emotional violence in 50 informal settlement clusters in Mumbai

Figure 3. Prevalence of physical, sexual, and emotional violence by age group, schooling, socioeconomic asset quintile, and disability

Table 1. Comparison of questions in five surveys describing physical, sexual, and emotional violence

Table 2. Characteristics of households, respondents, and their partners

Table 3. Lifetime and 12-month prevalence of domestic physical, sexual, and emotional violence, by perpetrator: intimate partner, marital family, or natal family member

Table 4. Lifetime prevalence of domestic control, by perpetrator: intimate partner, marital family, or natal family member

Competing interests

The authors declare no competing interest.

References

1. Ellsberg M, Jansen H, Heise L, Watts C, Garcia-Moreno C, on behalf of the WHO Multi-country Study on Women's Health and Domestic Violence against Women Study Team. Intimate partner violence and women's physical and mental health in the WHO multi-country study on women's health and domestic violence: an observational study. Lancet. 2008;371:1165-72.
2. Stöckl H, Devries K, Rotstein A, Abrahams N, Campbell J, Watts C, et al. The global prevalence of intimate partner homicide: a systematic review. Lancet. 2013;382:859-65.

For peer review only

3. Satyanarayana VA, Chandra PS, Vaddiparti K. Mental health consequences of violence against women and girls. Curr Opin Psychiatry. 2015;28:350-6.
4. WHO. Global and regional estimates of violence against women: prevalence and health effects of intimate partner violence and non-partner sexual violence. Geneva: World Health Organization; 2013. Report No.: 9789241564625.
5. Garcia-Moreno C, Zimmerman C, Morris-Gehring A, Heise L, Amin A, Abrahams N, et al. Addressing violence against women: a call to action. Lancet. 2015;385:1685-95.
6. Solotaroff JL, Pande RP. Violence against women and girls: lessons from South Asia. Washington DC; 2014.
7. UN General Assembly. Declaration on elimination of violence against women. GA res. 48/104, 48 UN GAOR. Supp. No. 49 at 217, UN Doc. A/48/49. www1.umn.edu/humanrts/instree/e4devw.htm; 1993.
8. UNDAW. Indicators to measure violence against women. Report of the Expert Group Meeting. Geneva: United Nations Division for the Advancement of Women, United Nations Economic Commission for Europe, United Nations Statistical Division; 2007.
9. Gelles RJ. Estimating the incidence and prevalence of violence against women. Violence Against Women. 2000;6:784-804.
10. Saltzman LE. Definitional and methodological issues related to transnational research on intimate partner violence. Violence Against Women. 2004;10:812-30.
11. Kilpatrick DG. What is violence against women? Defining and measuring the problem. J Interpers Violence. 2004;19:1209-34.
12. Heise L. Violence against women: an integrated ecological framework. Violence Against Women. 1998;4:262-90.
13. Piedalue A. Understanding violence in place: travelling knowledge paradigms and measuring domestic violence in India. Ind J Gender Stud. 2015;22.
14. Garcia-Moreno C, Jansen HA, Ellsberg M, Heise L, Watts C. WHO multi-country study on women's health and domestic violence against women: initial results on prevalence, health outcomes and women's responses. Geneva: World Health Organization; 2005.
15. Johnson H, Ollus N, Nevala S. Violence against women: an international perspective. New York NY: European Institute for Crime Prevention and Control (HEUNI); 2008.
16. Waltermaurer E. Measuring intimate partner violence (IPV): you may only get what you ask for. J Interpers Violence. 2005;20:501-6.
17. Thompson MP, Basile KC, Hertz MF, Sitterle D. Measuring intimate partner violence victimization and perpetration: a compendium of assessment tools. Atlanta GA: Centers for Disease Control and Prevention, National Center for Injury Prevention and Control; 2006.
18. Ruiz-Pérez I, Plazaola-Castaño J, Vives-Cases C. Methodological issues in the study of violence against women. J Epidemiol Commun Health. 2007;61:ii26-ii31.
19. Bagwell-Gray ME, Messing JT, Baldwin-White A. Intimate partner sexual violence: a review of terms, definitions, and prevalence. Trauma Violence Abuse. 2015;16:316-35.
20. Straus M. Measuring intrafamily conflict and violence: the Conflict Tactics (CT) Scales. J Marriage Family. 1979;41:75-88.
21. Straus M, Hamby S, Boney-McCoy S, Sugarman D. The Revised Conflict Tactics Scales (CTS2) development and preliminary psychometric data. J Family Issues. 1996;17:283-316.
22. Tjaden P. Defining and measuring violence against women: background, issues, and recommendations. Geneva: UN Division for the Advancement of Women, Economic Commission for Europe, World Health Organization; 2005.
23. Ferraro KJ, Johnson JM. How women experience battering: the process of victimization. Social Problems. 1983;30:325-39.
24. Mills T. The assault on the self: stages in coping with battering husbands. Qualitative Sociology. 1985;8:103-23.
25. Decker MR, Nair S, Saggurti N, Sabri B, Jethva M, Raj A, et al. Violence-related coping, help-seeking and health care-based intervention preferences among perinatal women in Mumbai, India. J Interpers Violence. 2013;28:1924-47.

For peer review only - http://bmjopen.bmj.com/site/about/guidelines.xhtml

26. Jejeebhoy S, Santhya KG, Acharya R. Violence against women in South Asia: the need for the active engagement of the health sector. Global Public Health. 2014;9:678-90.
27. Ragavan M, Iyengar K, Wurtz R. Perceptions of options available for victims of physical intimate partner violence in northern India. Violence Against Women. 2015;21:652-75.
28. Leonardsson M, San Sebastion M. Prevalence and predictors of help-seeking for women exposed to spousal violence in India - a cross-sectional study. BMC Women's Health. 2017;17:99.
29. Vranda MN, Kumar CN, Muralidhar D, Janardhana N, Sivakumar PT. Barriers to disclosure of intimate partner violence among female patients availing services at tertiary care psychiatric hospitals: a qualitative study. J Neurosci Rural Pract. 2018;9:326-30.
30. United Nations. Convention on the elimination of all forms of discrimination against women. http://www.un.org/womenwatch/daw/cedaw/. New York: United Nations; 1979.
31. Kalokhe A, del Rio C, Dunkle K, Stephenson R, Metheny N, Paranjape A, et al. Domestic violence against women in India: A systematic review of a decade of quantitative studies. Global Public Health. 2016:1-16.
32. Kalokhe AS, Potdar RR, Stephenson R, Dunkle KL, Paranjape A, del rio C, et al. How well does the World Health Organization definition of domestic violence work for India? PLoS ONE. 2015;10:e0120909.
33. Silverman JG, Donta B, Decker MR, Boyce SC, Ritter J, Naik DD, et al. Family violence and maltreatment of women during the perinatal period: associations with infant morbidity in Indian slum communities. Matern Child Health J. 2016;20:149-57.
34. Kalokhe AS, Stephenson R, Kelley ME, Dunkle KL, Paranjape A, Solas V, et al. The Development and Validation of the Indian Family Violence and Control Scale. PLoS One. 2016;11(1):e0148120.
35. Ruikar MM, Pratinidhi AK. Physical wife abuse in an urban slum of Pune, Maharashtra. Ind J Public Health. 2008;52:215-7.
36. Sinha A, Mallik S, Sanyal D, Dasgupta S, Pal D, Mukherjee A. Domestic violence among ever married women of reproductive age group in a slum area of Kolkata. Indian J Public Health. 2012;56(1):31-6.
37. Shrivastava PS, Shrivastava SR. A study of spousal domestic violence in an urban slum of Mumbai. Int J Prev Med. 2013;4:27-32.
38. Sabri B, Campbell JC. Intimate partner violence against women in slums in India. Ind J Med Res. 2015;141:757-9.
39. Office of the Registrar General and Census Commissioner. Census of India 2011. Provisional population totals. Urban agglomerations and cities. (http://censusindia.gov.in/2011-prov-results/paper2/data_files/India2/1.%20Data%20Highlight.pdf). New Delhi: Government of India, MInistry of Home Affairs; 2012.
40. Central Bureau of Health Intelligence. National health profile 2015. New Delhi: Directorate General of Health Services, Ministry of Health and Family Welfare; 2015.
41. Chandramouli C. Housing stock, amenities and assets in slums - Census 2011. Available from www.censusindia.gov.in. New Delhi: Office of the Registrar General and Census Commissioner,; 2011.
42. United Nations Human Settlements Programme (UN-Habitat). The challenge of slums: global report on human settlements 2003. London and Sterling VA: Earthscan Publications Ltd; 2003.
43. Ministry of Housing and Urban Poverty Alleviation. Report of the Committee on Slum Statistics/Census. New Delhi: Government of India; National Buildings Organisation; 2010.
44. Daruwalla N, Machchhar U, Pantvaidya S, D'Souza V, Gram L, Copas A, et al. Community interventions to prevent violence against women and girls in informal settlements in Mumbai: the SNEHA-TARA pragmatic cluster randomised controlled trial. Trials. 2019;20(1):743.

For peer review only

For peer review only - http://bmjopen.bmj.com/site/about/guidelines.xhtml

45. Osrin D, Das S, Bapat U, Alcock GA, Joshi W, More NS. A rapid assessment scorecard to identify informal settlements at higher maternal and child health risk in Mumbai. J Urban Health. 2011;88(5):919-32.
46. Taljaard M, Weijer C, Grimshaw JM, Eccles MP. The Ottawa Statement on the ethical design and conduct of cluster randomised trials: precis for researchers and research ethics committees. BMJ. 2013;346:f2838.
47. Lignou S, Das S, Mistry J, Alcock G, More NS, Osrin D, et al. Reconstructing communities in cluster trials? Trials. 2016;17:166.
48. WHO. Putting women first: ethical and safety recommendations for research on domestic violence against women. WHO/FCH/GWH/01.1. Geneva: Department of Gender and Women's Health, Family and Community Health, World Health Organization; 2001.
49. Jewkes R, Dartnall E, Sikweyiya Y. Ethical and safety recommendations for research on perpetration of sexual violence. Pretoria: Medical Research Council, Sexual Violence Research Initiative; 2012.
50. IIPS. National Family Health Survey 2015-16 (NFHS-4): interviewer's manual. Mumbai: International Institute for Population Sciences; 2014.
51. Washington Group on Disability Statistics (WG). Understanding and interpreting disability as measured using the WG Short Set of questions. Hyattsville MD; 2009.
52. Filmer D, Pritchett L. Estimating wealth effects without expenditure data - or tears: an application to educational enrollments in states of India. Demography. 2001;38:115-32.
53. Vyas S, Kumaranayake L. Constructing socio-economic status indices: how to use principal components analysis. Health Policy Plan. 2006;21:459-68.
54. WHO. Consensus on priority indicators on violence against women Geneva2020 [Available from: https://www.who.int/reproductivehealth/topics/violence/vaw-indicators/en/.
55. George J, Nair D, Premkumar NR, Saravanan N, Chinnakali P, Roy G. The prevalence of domestic violence and its associated factors among married women in a rural area of Puducherry, South India. J Family Med Prim Care. 2016;5(3):672-6.
56. Ram A, Victor CP, Christy H, Hembrom S, Cherian AG, Mohan VR. Domestic violence and its determinants among 15-49-year-old women in a rural block in South India. Ind J Commun Med. 2019;44:362-7.
57. Richardson R, Nandi A, Jaswal S, Harper S. The effect of intimate partner violence on women's mental distress: a prospective cohort study of 3010 rural Indian women. Soc Psychiatry Psychiatr Epidemiol. 2019.
58. Mukherjee R, Joshi RK. Controlling Behavior and Intimate Partner Violence: A Cross-Sectional Study in an Urban Area of Delhi, India. J Interpers Violence. 2019:886260519876720.
59. Nadda A, Malik JS, Rohilla R, Chahal S, Chayal V, Arora V. Study of domestic violence amoung currently married females of Haryana, India. Ind J Psychol Med. 2018;40:534-9.
60. Sharma KK, Vatsa M, Kalaivani M, Bhardwaj D. Mental health effects of domestic violence against women in Delhi: A community-based study. J Family Med Prim Care. 2019;8(7):2522-7.
61. Begum S, Donta B, Nair S, Prakasam CP. Socio-demographic factors associated with domestic violence in urban slums, Mumbai, Maharashtra, India Indian J Med Res. 2015;141:783-8.
62. International Institute for Population Sciences (IIPS), ICF. National Family Health Survey (NFHS-4), India, 2015-16: Maharashtra. Mumbai: IIPS; 2018.
63. Ahmad J, Khan NH, Mozumdar A. Spousal Violence Against Women in India: A Social-Ecological Analysis Using Data From the National Family Health Survey 2015 to 2016. J Interpers Violence. 2019(Oct 23):886260519881530.
64. Kumar V, Kanth S. Bride burning. Lancet. 2004;364 Suppl 1:s18-9.
65. Kandiyoti D. Bargaining with patriarchy. Gender and Society. 1988;2:274-90.
66. Ragavan M, Iyengar K. Violence perpetrated by mothers-in-law in Northern India: perceived frequency, acceptability, and options for survivors. J Interpers Violence. 2017(May 1):886260517708759.

For peer review only - http://bmjopen.bmj.com/site/about/guidelines.xhtml

67. Ritter J, Dasgupta A, Raj A, Decker MR, Nair S, Trivedi A, et al. Measuring gender-based household maltreatment in urban slums of Mumbai, India. J Interpers Violence. 2019;1-23(Jul 31):886260519864370.
68. Heise L, Pallitto C, García-Moreno C, Clark CJ. Measuring psychological abuse by intimate partners: constructing a cross-cultural indicator for the Sustainable Development Goals. SSM Popul Health. 2019;9:100377.
69. Kundapur R, Shetty SM, Kempaller VJ, Kumar A, Anurupa M. Violence against Educated Women by Intimate Partners in Urban Karnataka, India. Indian J Community Med. 2017;42(3):147-50.
70. Silverman JG, Boyce SC, Dehingia N, Rao N, Chandurkar D, Nanda P, et al. Reproductive coercion in Uttar Pradesh, India: Prevalence and associations with partner violence and reproductive health. SSM Popul Health. 2019;9:100484.
71. Jewkes R. Emotional abuse: a neglected dimension of partner violence. Lancet. 2010;376:851-2.
72. Follingstad DR, Rutledge LL, Berg BJ, Hause ES, Polek DS. The role of emotional abuse in physically abusive relationships. J Family Violence. 1990;5:107-20.
73. Vitanza S, Vogel LCM, Marshall LL. Distress and symptoms of posttraumatic stress disorder in abused women. Violence Victims. 1995;10:23-34.
74. Mechanic MB, Weaver TL, Resick PA. Mental health consequences of intimate partner abuse: a multidimensional assessment of four different forms of abuse. Violence Against Women. 2008;14:634-54.
75. Coker AL, Davis KE, Arias I, Desai S, Sanderson M, Brandt HM, et al. Physical and mental health effects of intimate partner violence for men and women. Am J Prev Med. 2002;23:260-8.
76. Kumar S, Jeyaseelan L, Suresh S, Ahuja RC. Domestic violence and its mental health correlates in Indian women. Br J Psychiatry. 2005;187:62-7.
77. Stephenson R, Winter A, Hindin M. Frequency of intimate partner violence and rural women's mental health in four Indian states. Violence Against Women. 2013;19:1133.
78. Gelles RJ. Abused wives: why do they stay. J Marriage Family. 1976;38:659-68.
79. Anitha S. Understanding economic abuse through an intersectional lens: financial abuse, control, and exploitation of women’s productive and reproductive labor. Violence Against Women. 2019;25:1854-77.
80. Jejeebhoy SJ, Cook RJ. State accountability for wife-beating: the Indian challenge. Lancet. 1997;349(Suppl 1):s10-2.
81. Narula DK, Narula A. Patriarchal societies and domestic violence: need for empowering women. In: Charak P, Mohan C, editors. Women and development: self, society and empowerment. New Delhi: Primus Books; 2012. p. 169-83.
82. Ahmed-Ghosh H. Chattels of society: domestic violence in India. Violence Against Women. 2004;10:94-118.
83. Jejeebhoy SJ, Santhya KG, Sabarwal S. Gender-based violence: a qualitative exploration of norms, experiences and positive deviance. New Delhi: Population Council; 2013.
84. Ghosh S. Watching, blaming, silencing, intervening: exploring the role of the community in preventing domestic violence in India. Practicing Anthropology. 2011;33:22-6.

For peer review only

Table 1. Comparison of questions in five surveys describing physical, sexual, and emotional violence

	SNEHA TARA	NFHS-4 (50)	Indian Family Violence and Control Scale (34)	WHO multi-country study (14)	International Violence Against Women Survey (15)
**Physical**					
Pushed, shoved, shaken, hurt	√	√	√	√	√
Twisted arm, banged head, pulled hair	√	√	√		√
Slapped, pinched, bitten	√	√	√	√	√
Hit, punched	√	√	√	√	
Kicked, dragged, beaten	√	√	√	√	
Things thrown at, burned	√		√	√	
Attacked or threatened with sharp object	√	√	√	√	√
Attacked or threatened with blunt object	√	√	√	√	√
Suffocated, choked, hung, poisoned	√	√	√	√	√
**Sexual**					
Forced intercourse	√	√	√	√	√
Forced other degrading act	√	√	√	√	
Threatened other act	√	√			
Forced to replicate pornography	√		√		
Forced sex during menses	√		√		
Forced sex with someone else	√		√		
Forced sex without contraception	√		√		
Threatened sex with another person	√		√		
Forced video of sex	√		√		
Taken advantage of when drugged or drunk	√		√		
Forced to watch pornography	√				
Insisted on repeated intercourse	√				
Forced to entertain others sexually	√				
Forced drugs or alcohol for sex	√				
Partner withheld sexual pleasure	√				
**Emotional**					
Insulted, made to feel bad about herself	√	√	√	√	
Belittled, humiliated in front of others	√	√	√	√	
Ignored, treated indifferently	√		√	√	
Scared or intimidated on purpose	√		√	√	
Threated to hurt her, someone close, or take child	√	√		√	√
Insulted for not having a baby	√		√		
Insulted for not having a son	√		√		
Afraid of family members	√				
Accused of infidelity	√	√		√	
Insulted for being a woman	√				

For peer review only

For peer review only - http://bmjopen.bmj.com/site/about/guidelines.xhtml

Table 2. Characteristics of households, respondents, and their partners

**Household**	n	(%)
**Fabric**		
Kachha (insubstantial)	353	(7)
Mixed	2149	(42)
Pukka (robust)	2620	(51)
**Toilet**		
Private	875	(17)
Public or charity	4245	(83)
Open defecation	2	(0)
**Religion**		
Hindu	3002	(59)
Muslim	1902	(37)
Buddhist	158	(3)
Other	60	(1)
Total	5122	(100)

**Individual**	Woman respondent		Husband or partner	
	n	(%)	n	(%)
**Age group**				
18-19 y	115	(2)	14	(0)
20-29 y	2038	(40)	920	(19)
30-39 y	2056	(40)	2108	(44)
40-49 y	913	(18)	1372	(29)
50+ y			391	(8)
**Education**				
None	947	(18)	474	(10)
Primary 1-5 y	859	(17)	590	(12)
Middle 6-8 y	1122	(22)	981	(20)
High 9-10 y	1158	(23)	1543	(32)
Senior 11-12 y	572	(11)	698	(15)
Undergraduate	363	(7)	371	(8)
Postgraduate	100	(2)	123	(3)
Other	1	(0)	25	(1)
**Paid work in last 12 m**				
Yes	1252	(24)	4697	(98)
No	3870	(76)	108	(2)
Total	5122	(100)	4805	(100)

For peer review only

Table 3. Lifetime and 12-month prevalence of domestic physical, sexual, and emotional violence, by perpetrator: intimate partner, marital family, or natal family member

	Lifetime		Lifetime by perpetrator						Last 12 m		Last 12 m by perpetrator					
	Domestic		Intimate partner		Marital		Natal		Domestic		Intimate partner		Marital		Natal	
	n	(%)	n	(%)	n	(%)	n	(%)	n	(%)	n	(%)	n	(%)	n	(%)
**Physical violence**																
Pushed, shoved, shaken, hurt	595	(11.6)	458	(8.9)	186	(3.6)	33	(0.6)	292	(5.7)	247	(4.8)	70	(1.4)	17	(0.3)
Twisted arm, banged head, pulled hair	464	(9.1)	371	(7.2)	121	(2.4)	23	(0.4)	224	(4.4)	194	(3.8)	43	(0.8)	10	(0.2)
Slapped, pinched, bitten	1154	(22.5)	986	(19.3)	134	(2.6)	139	(2.7)	565	(11.0)	504	(9.8)	52	(1.0)	71	(1.4)
Hit, punched	421	(8.2)	351	(6.9)	90	(1.8)	28	(0.5)	206	(4.0)	182	(3.6)	30	(0.6)	10	(0.2)
Kicked, dragged, beaten	398	(7.8)	326	(6.4)	88	(1.7)	28	(0.5)	199	(3.9)	175	(3.4)	32	(0.6)	10	(0.2)
Things thrown at, burned	51	(1.0)	41	(0.8)	14	(0.3)	0	(0.0)	15	(0.3)	13	(0.3)	3	(0.1)	0	(0.0
Attacked or threatened with sharp object	96	(1.9)	74	(1.4)	22	(0.4)	3	(0.1)	51	(1.0)	44	(0.9)	6	(0.1)	2	(0.0)
Attacked or threatened with blunt object	218	(4.3)	165	(3.2)	51	(1.0)	22	(0.4)	103	(2.0)	90	(1.8)	16	(0.3)	6	(0.1)
Suffocated, choked, hung, poisoned	114	(2.2)	96	(1.9)	20	(0.4)	5	(0.1)	52	(1.0)	48	(0.9)	8	(0.2)	1	(0.0)
**Sexual violence**																
Forced intercourse	231	(4.5)	219	(4.3)	6	(0.1)	4	(0.1)	138	(2.7)	136	(2.7)	1	(0.0)	0	(0.0)
Forced other degrading act	128	(2.5)	126	(2.5)	1	(0.0)	1	(0.0)	95	(1.9)	95	(1.9)	1	(0.0)	0	(0.0)
Threatened other act	74	(1.4)	72	(1.4)	0	(0.0)	1	(0.0)	43	(0.8)	43	(0.8)	0	(0.0)	0	(0.0)
Forced to replicate pornography	75	(1.5)	75	(1.5)	0	(0.0)	0	(0.0)	45	(0.9)	45	(0.9)	0	(0.0)	0	(0.0)
**Emotional violence**																
Insulted, made to feel bad about herself	1296	(25.3)	536	(10.5)	877	(17.1)	99	(1.9)	690	(13.5)	347	(6.8)	419	(8.2)	64	(1.2)
Ignored, treated indifferently	773	(15.1)	339	(6.6)	541	(10.6)	50	(1.0)	449	(8.8)	230	(4.5)	290	(5.7)	34	(0.7)
Belittled, humiliated in front of others	682	(13.3)	313	(6.1)	445	(8.7)	52	(1.0)	417	(8.1)	223	(4.4)	242	(4.7)	35	(0.7)
Scared or intimidated on purpose	841	(16.4)	592	(11.6)	330	(6.4)	67	(1.3)	570	(11.1)	432	(8.4)	191	(3.7)	52	(1.0)
Threated to hurt her, someone close, or take child away	213	(4.2)	147	(2.9)	100	(2.0)	6	(0.1)	130	(2.5)	100	(2.0)	49	(1.0)	4	(0.1)
**Any physical violence**	1293	(25.2)	1084	(21.2)	261	(5.1)	170	(3.3)	644	(12.6)	562	(11.0)	103	(2.0)	84	(1.6)
**Any sexual violence**	289	(5.6)	277	(5.4)	7	(0.1)	4	(0.1)	188	(3.7)	186	(3.6)	2	(0.0)	0	(0.0)
**Any emotional violence**	1599	(31.2)	876	(17.1)	1045	(20.4)	138	(2.7)	963	(18.8)	607	(11.9)	538	(10.5)	90	(1.8)
**Total**	**5122**	**(100.0)**	**5122**	**(100.0)**	**5122**	**(100.0)**	**5122**	**(100.0)**	**5122**	**(100.0)**	**5122**	**(100.0)**	**5122**	**(100.0)**	**5122**	**(100.0)**

For peer review only

For peer review only - http://bmjopen.bmj.com/site/about/guidelines.xhtml

Table 4. Lifetime prevalence of domestic control, by perpetrator: intimate partner, marital family, or natal family member

Control	Domestic		Intimate partner		Marital		Natal	
	n	(%)	n	(%)	n	(%)	n	(%)
**Mobility**								
Movement always monitored	419	(8.2)	278	(5.4)	140	(2.7)	44	(0.9)
Always prevented from attending meetings	227	(4.4)	129	(2.5)	118	(2.3)	5	(0.1)
**Education**								
Prevented from schooling	152	(3.0)	53	(1.0)	47	(0.9)	62	(1.2)
**Employment**								
Prevented from seeking employment	853	(16.7)	728	(14.2)	162	(3.2)	31	(0.6)
Coerced to seek employment	78	(1.5)	42	(0.8)	35	(0.7)	7	(0.1)
**Home dynamics**								
Excluded from family matters	366	(7.1)	108	(2.1)	300	(5.9)	14	(0.3)
Dress or hairstyle always controlled	267	(5.2)	119	(2.3)	145	(2.8)	25	(0.5)
Limited access to house	173	(3.4)	56	(1.1)	137	(2.7)	7	(0.1)
Forced out of house	165	(3.2)	52	(1.0)	121	(2.4)	5	(0.1)
Locked in house	39	(0.8)	18	(0.4)	22	(0.4)	1	(0.0)
**Health**								
Made to do excessive work	239	(4.7)	39	(0.8)	209	(4.1)	11	(0.2)
Always requires permission for healthcare	753	(14.7)	513	(10.0)	286	(5.6)	48	(0.9)
**Any of the above**	**1816**	**(35.4)**	**1303**	**(25.4)**	**830**	**(16.2)**	**184**	**(3.6)**
**Total**	5122	(100.0)	5122	(100.0)	5122	(100.0)	5122	(100.0)

For peer review only

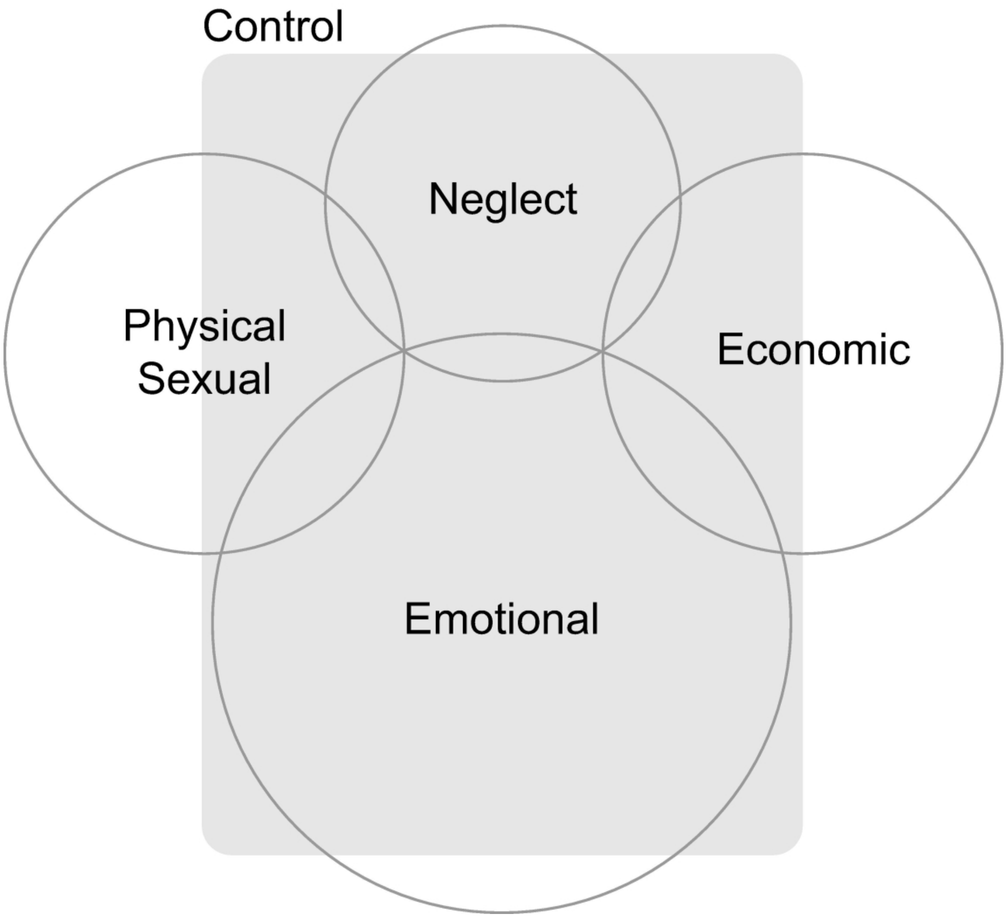

Figure 1. Forms of domestic violence against women

143x130mm (600 x 600 DPI)

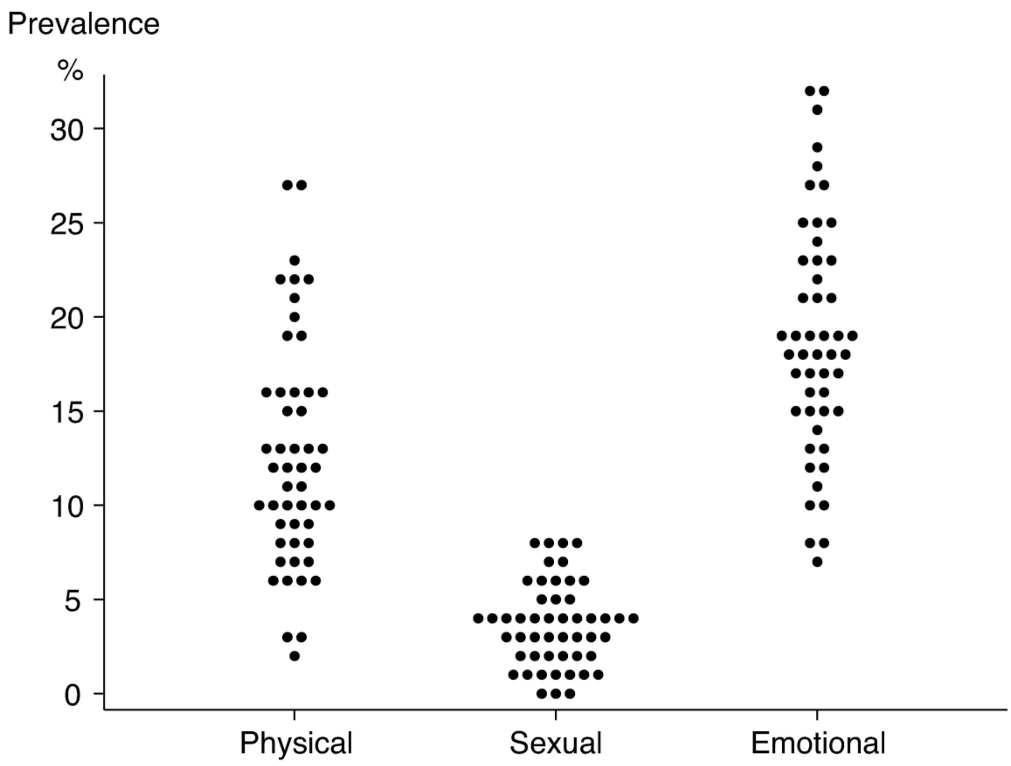

Figure 2. Prevalence of physical, sexual, and emotional violence against 5122 women aged 18-49 years in 50 informal settlement clusters in Mumbai

138x105mm (600 x 600 DPI)

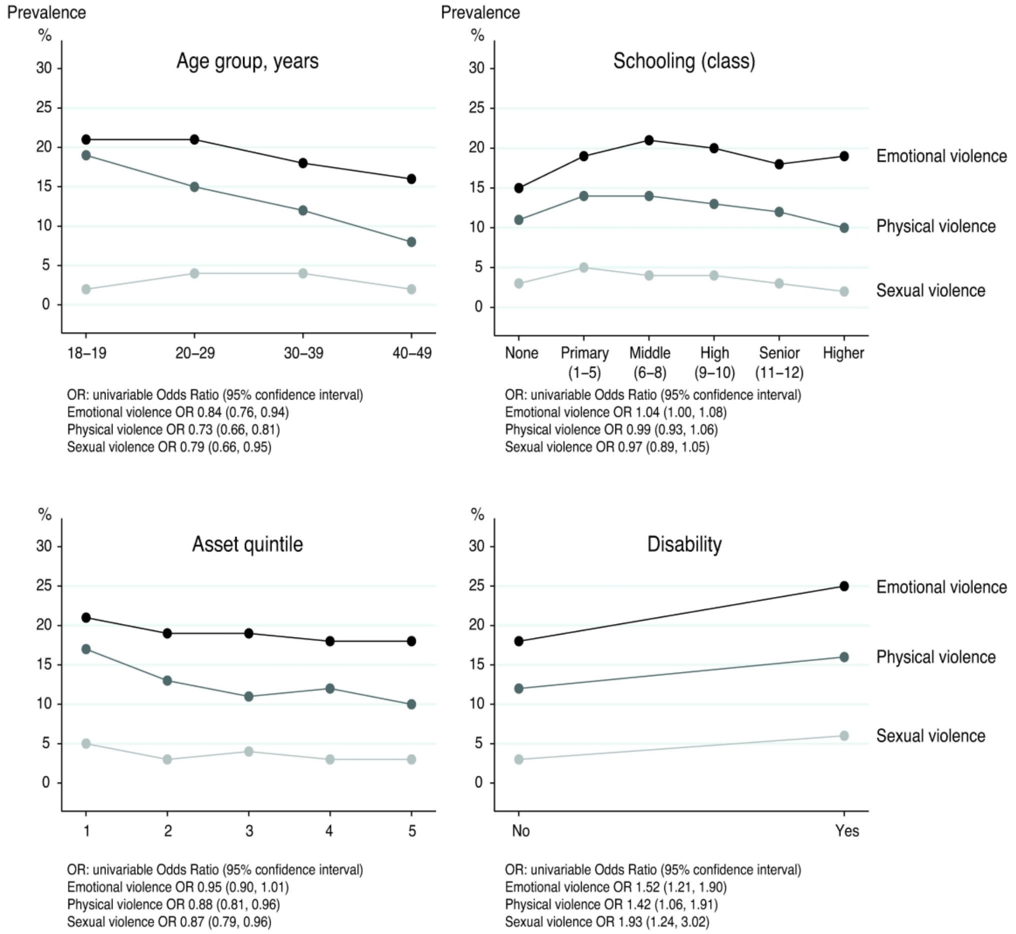

Figure 3. Prevalence of physical, sexual, and emotional violence against 5122 women aged 18-49 years, by age group, schooling, socioeconomic asset quintile, and disability

195x181mm (600 x 600 DPI)

BMJ Open

Prevalence of domestic violence against women in informal settlements in Mumbai, India: a cross-sectional survey

Journal:	*BMJ Open*
Manuscript ID	bmjopen-2020-042444.R1
Article Type:	Original research
Date Submitted by the Author:	26-Aug-2020
Complete List of Authors:	Daruwalla, Nayreen; Society for Nutrition, Education & Health Action (SNEHA)<br>Kanougiya, Suman; Society for Nutrition, Education & Health Action (SNEHA)<br>Gupta, Apoorwa; Society for Nutrition, Education & Health Action (SNEHA)<br>Gram, Lu; University College London, Institute for Global Health<br>Osrin, David; University College London, Institute for Global Health
**Primary Subject Heading**:	Public health
Secondary Subject Heading:	Epidemiology, Global health
Keywords:	PUBLIC HEALTH, PRIMARY CARE, EPIDEMIOLOGY

SCHOLARONE™ Manuscripts

For peer review only - http://bmjopen.bmj.com/site/about/guidelines.xhtml

I, the Submitting Author has the right to grant and does grant on behalf of all authors of the Work (as defined in the below author licence), an exclusive licence and/or a non-exclusive licence for contributions from authors who are: i) UK Crown employees; ii) where BMJ has agreed a CC-BY licence shall apply, and/or iii) in accordance with the terms applicable for US Federal Government officers or employees acting as part of their official duties; on a worldwide, perpetual, irrevocable, royalty-free basis to BMJ Publishing Group Ltd ("BMJ") its licensees and where the relevant Journal is co-owned by BMJ to the co-owners of the Journal, to publish the Work in this journal and any other BMJ products and to exploit all rights, as set out in our licence.

The Submitting Author accepts and understands that any supply made under these terms is made by BMJ to the Submitting Author unless you are acting as an employee on behalf of your employer or a postgraduate student of an affiliated institution which is paying any applicable article publishing charge ("APC") for Open Access articles. Where the Submitting Author wishes to make the Work available on an Open Access basis (and intends to pay the relevant APC), the terms of reuse of such Open Access shall be governed by a Creative Commons licence – details of these licences and which Creative Commons licence will apply to this Work are set out in our licence referred to above.

Other than as permitted in any relevant BMJ Author's Self Archiving Policies, I confirm this Work has not been accepted for publication elsewhere, is not being considered for publication elsewhere and does not duplicate material already published. I confirm all authors consent to publication of this Work and authorise the granting of this licence.

For peer review only

Prevalence of domestic violence against women in informal settlements in Mumbai, India: a cross-sectional survey

Authors

Nayreen Daruwalla

Program on Prevention of Violence Against Women and Children, SNEHA, Mumbai, Maharashtra, 400017, India

nayreen@snehamumbai.org

Suman Kanougiya

Program on Prevention of Violence Against Women and Children, SNEHA, Mumbai, Maharashtra, 400017, India

suman.kanougiya@gmail.com

Apoorwa Gupta

Program on Prevention of Violence Against Women and Children, SNEHA, Mumbai, Maharashtra, 400017, India

apoorwa.gupta@snehamumbai.org

Lu Gram

Institute for Global Health, University College London, London, WC1N IEH, UK

lu.gram.13@ucl.ac.uk

David Osrin (corresponding author)

Institute for Global Health, University College London, London, WC1N IEH, UK

d.osrin@ucl.ac.uk

For peer review only

Abstract

Introduction

Domestic violence against women harms individuals, families, communities, and society. Perpetrated by intimate partners or other family members, its overlapping forms include physical, sexual, and emotional violence, control and neglect. We aimed to describe the prevalence of these forms of violence and their perpetrators in informal settlements in Mumbai.

Methods

A cross-sectional survey in two large informal settlement areas aimed to include approximately 5000 participants (~100 women aged 18-49 years in each of 50 clusters). The questionnaire included questions on general health, household control and neglect, experience of physical, sexual, or emotional violence, and disclosure. We present responses to individual questions and composite indicators.

Results

The survey included 5122 respondents, of whom 644 (13%) had experienced physical domestic violence in the last year, 188 (4%) sexual violence, and 963 (19%) emotional violence. 13% of ever-married women had experienced physical or sexual intimate partner violence in the last year. Most physical (87%) and sexual violence (99%) was done by partners, but emotional violence equally involved marital family members. All three forms of violence were more common if women were younger, in the lowest socioeconomic asset quintile, or reported disability. 1816 women (35%) had experienced at least one instance of coercive control and 33% said that they were afraid of people in their home. 10% reported domestic neglect of their food, sleep, health, or children's health.

Conclusion

Domestic violence against women remains common in urban informal settlements. Physical and sexual violence was perpetrated mainly by intimate partners, but emotional violence was attributed equally to partners and marital family. More than one-third of women described controlling behaviours perpetrated by both intimate partners and marital family members. We emphasise the need to include the spectrum of perpetrators and forms of domestic violence – particularly emotional violence and coercive control - in data gathering.

Key words

Domestic Violence; Gender-based Violence; Intimate Partner Violence; Mumbai; India

For peer review only

For peer review only - http://bmjopen.bmj.com/site/about/guidelines.xhtml

Article summary

Strengths and limitations of this study

- In a large cross-sectional survey of 5122 women in informal settlements, we found that 13% had suffered physical and 4% sexual domestic violence in the last year, mostly from their intimate partners.
- However, 19% had suffered emotional violence, 35% had been subjected to controlling behavior, and 10% had suffered neglect of their food, sleep, health, or children's health.
- These forms of domestic violence were perpetrated by both intimate partners and in-laws.
- The full spectrum of domestic violence—particularly emotional violence and coercive control—and the potential range of perpetrators are sources of concern.
- Limitations include the cross-sectional nature of the study, the possibility of under-reporting, and an emphasis on younger married women.

For peer review only

Background

The global burden of domestic violence is underlined by its inclusion as an indicator of gender equality for the fifth United Nations Sustainable Development Goal (https://sdg-tracker.org/gender-equality). Domestic violence against women harms individuals, families, communities, and society. For individuals, it causes injury or death (1, 2), reproductive health problems, harmful drug and alcohol use, anxiety, depression, post-traumatic stress disorder, self-harm, and suicide (1, 3). For families, it leads to miscarriage, induced abortion, stillbirth, low birth weight, preterm delivery, and further violence (4, 5). For communities and society, it leads to lack of agency, limited participation, and lost economic productivity (5, 6).

Globally, 30% of women have survived physical or sexual violence by an intimate partner or sexual violence by a non-partner (4). The precision of the wording of this statement illustrates the challenge of defining violence against women. The United Nations Declaration on the Elimination of Violence against Women defines it as ‘‘any act of gender-based violence that results in, or is likely to result in, physical, sexual or psychological harm or suffering to women, including threats of such acts, coercion or arbitrary deprivation of liberty, whether occurring in public or in private life” (7). This definition allows for a range of perpetrators and a range of forms of violence. The perpetrator may be a husband or partner (intimate partner violence), a family or household member, or someone known or unknown outside the domestic environment (in this paper, we define domestic violence as falling into either or both of the first two categories). Figure 1 conceptualises the intersecting forms of domestic violence: physical, sexual, emotional or psychological, control, neglect, or economic. These forms of violence overlap and occur in combination, in some settings with forms such as female genital mutilation and modern slavery.

There is consensus that measuring the scope and prevalence of violence against women is important, and that we should try to measure its different sources and forms (8). Doing so may help identify the harm it can cause, advocate on behalf of individual women and against an unacceptable societal burden of coercive control (a perspective that owes much to the work of feminists over the last half century), and compare research findings across time and place and in a shared language (9). We should be aware, however, that an emphasis on definition and metrics is characteristic of a public health approach (10). As feminists have emphasized, violence against women is equally a human rights and criminal justice concern (11), the product of an ecology of micro, meso, and macro factors (12), and a manifestation of structural violence and gendered power imbalances. Too tight a focus on its epidemiology might depoliticize violence against women and shift attention away from these issues (13).

Surveys have been important in international efforts to establish prevalence, the most prominent being the WHO multi-country study on women’s health and domestic violence against women (14) and the International Violence against Women Surveys (15). Choosing and comparing between the available scales and questionnaires is difficult (16, 17). The challenges to measurement are substantial (18), not least in terms of definitions and terminology (19). Early surveys focused on intimate partner physical violence. The Conflict Tactics Scale (20), on which they have often been based, has been extended to include sexual and emotional violence (21), and the definition has been extended to include perpetrators other than intimate partners (22). Whatever has been achieved in terms of definitions and scales, a fundamental challenge remains disclosure: women may not perceive, acknowledge, or communicate that they are surviving violence (23, 24). This is a central issue for clinical and social service provision (25-29), makes crime and hospital records difficult to use in estimating prevalence, and extends to interviews in surveys.

India is a signatory to the Convention on the Elimination of All Forms of Discrimination Against Women (30). Violence against women is addressed by criminal law, particularly domestic violence in Section 498-A of the Indian Penal Code, and by civil law in the form of the Protection of Women from Domestic Violence Act, 2005, which encompasses the varied forms of domestic violence and its perpetrators (available at

For peer review only - http://bmjopen.bmj.com/site/about/guidelines.xhtml

https://indiacode.nic.in/bitstream/123456789/2021/1/200543.pdf). A systematic review of 137 studies suggested that 22% of women in India had survived physical violence in the past year (131 studies), 22% had suffered emotional or verbal psychological violence (60 studies), 7% sexual violence (79 studies), and 30% multiple forms of violence (31). Women in India also commonly experience forms of violence that extend to coercive control and neglect (32), articulated in the idea of gender-based household maltreatment (33), and captured in the recent Indian Family Violence and Control Scale (34).

We work on primary, secondary, and tertiary prevention of violence against women in informal settlements in Mumbai. The prevalence of physical and emotional violence in such settings has been reported as high (35-38). At the beginning of a programme of community mobilisation to prevent violence against women and girls, we conducted a baseline survey. Our objectives in this analysis were to estimate the prevalence of physical, sexual, and emotional violence, control and neglect. We were particularly interested in sources of domestic violence other than intimate partners and in forms of violence other than physical.

Methods

Setting

One in four of the world's urban residents live in informal settlements (slums) (39), characterized by overcrowding, insubstantial housing, insufficient water and sanitation, lack of tenure, and hazardous location (40). In India, the state of Maharashtra accounts for 18% of the national total: more than 100 million residents (41). Just over 40% of Mumbai homes are in informal settlements. The Census of India describes these as areas in which dwellings are unfit for human habitation as a result of dilapidation, overcrowding, and poor building and street design, with associated poor ventilation, light, and sanitation (42).

SNEHA (Society for Nutrition, Education and Health Action) is a non-government organization working to improve health in such settlements. The SNEHA programme on Prevention of Violence against Women and Children began in 2000. Primary prevention is addressed through a combination of community group activities and resulting individual voluntarism. Secondary prevention includes local crisis response and psychological first aid by community organisers and referral to centres which provide counselling, legal, and psychotherapeutic support, with links to the police and medical, shelter, and social service providers. Tertiary prevention is provided primarily through referral to psychiatric and legal services. Having satisfied criteria for counselling, shelter, legal aid, and access to medical care, SNEHA is a service provider under the Protection of Women from Domestic Violence Act, 2005, and is authorised to file Domestic Incident Reports. Under the Protection of Children from Sexual Offences Act, 2012, SNEHA has reporting rights to the police and Child Welfare Committee.

Design

We did a cross-sectional survey of women's experience of violence, collected at the beginning of a cluster randomized controlled trial of community interventions to prevent violence against women and girls (the SNEHA TARA trial) (43).

Sample size

We aimed to complete approximately 100 questionnaires in each of 50 clusters, giving a total sample of around 5000. An estimate of prevalence from a cross-sectional sample of 5000 in a population of 125,000 would have a precision of ~1%. Within this, a comparison of two categories of determinant for 100 respondents in each of 50 clusters would provide 80% power to detect a difference of 6% in prevalence estimates of 10-20%.

Sampling process

Potential clusters were identified in four phases in two areas of the city, and 50 were selected on the basis of (a) vulnerability assessed against a scorecard (44), (b) no known

For peer review only - http://bmjopen.bmj.com/site/about/guidelines.xhtml

plans for rehabilitation or demolition in the next two years, (c) absence of existing nongovernment organization programs addressing violence against women, (d) more than 75% of structures residential and less than 25% rental, and (e) clear separation from each other.

Data collection

Structures and homes within clusters were mapped and 16 women interviewers with graduate education and three months training visited households to enumerate residents and list possible participants. Interviewers began at a random start point and visited every second household to enroll participants. Inclusion criteria were that respondents should be women aged 18-49 years who were usual residents. When there was more than one potential respondent in a household, the investigator applied an algorithm that selected the youngest woman at risk of disability, followed by the youngest married woman, followed by the youngest unmarried woman.

The questionnaire included modules on general health and wellbeing, common mental disorder, household decision-making, household power and control, neglect, experience of economic, emotional, physical, and sexual violence, disclosure and support. Questions were taken from existing Hindi versions where possible. If not, they were translated from English, piloted in two clusters external to the trial, amended, and back-translated.

Interviewers were all women and provided both time and sufficient information for women to consider whether to participate. They were supported by three field supervisors with direct linkage to counselling services, available by phone at any time. The interview team visited the local police station and social services to discuss their activities before starting in each area. Interviewers worked in groups of 7-8 in one cluster at a time, accompanied by a supervisor. A pair worked together in each lane and administered interviews in adjacent households.

To ensure privacy, interviews were arranged by advance appointment and avoided times when partners or children were likely to return from work or school. Women were interviewed at home or in a local community office if they preferred it. The interview began with general questions about demography, household residents, education, socioeconomic position, maternity, and health. If a family member, neighbour, or friend entered, the interviewer went back to asking questions about general health. If the person showed signs of staying, the interview was terminated and completed over up to three repeat visits. As a result of the gatekeeper consent process, community members were aware that interviewers would be visiting people in their area and this limited curiosity and intrusion.

Data management

Interviewers used electronic tablets to enter information in a database in CommCare (www.dimagi.com). To optimize accuracy, the system included field constraints, lookup tables, and automated skip logic. Mobile connectivity allowed immediate contact between interviewers and supervisors. We examined variation in prevalence rates by interviewer and discussed performance in supervisory meetings. We selected for field observation interviewers who showed signs of deviation from the group average and provided feedback where necessary.

Statistical analysis

In developing our questionnaire, we aimed to provide data that would allow comparisons between studies and settings. We included questions from major surveys, international and national, and it should be possible to develop summary measures of violence against women using any of a number of combinations of questions. Our idea was to make the resulting anonymized dataset available so that others might use it, choosing specific

For peer review only - http://bmjopen.bmj.com/site/about/guidelines.xhtml

questions to facilitate comparison or pooling of data. Here we present composite indicators of lifetime and 12-month prevalence of physical, sexual, and emotional violence.

Table 1 summarises the questions in three surveys from India (our TARA Trial questionnaire, the National Family Health Survey (NFHS-4) (45), and the Indian Family Violence and Control Scale (34), and two international surveys (the International Violence Against Women Survey (15) and the WHO multi-country study (14)).

Our composite estimate of prevalence of physical violence included nine questions that were comparable across surveys, although there was variation in whether threats of violence were considered incidents of physical violence. The estimate of prevalence of domestic sexual violence included four questions. The comparable questions about sexual violence were similar, although only the TARA survey and the Indian Family Violence Survey included a question on coercion to replicate activities seen in pornographic material. The estimate of prevalence of domestic emotional violence included five questions that were comparable across surveys. We asked a series of questions about domestic coercive control, addressing mobility, education, employment, appearance, and pregnancy. For control and neglect, we present the individual questions because composite indicators are not yet established.

We used the Washington Group Short Set on Functioning questions to identify disability, including difficulty with seeing, hearing, walking, remembering, self-care, and speaking (http://www.washingtongroup-disability.com/washington-group-question-sets/short-set-of-disability-questions/). We took the most permissive of four possible approaches to classification, in which disability is registered if the respondent reports at least some difficulty in at least one of the six domains (46). We classified socioeconomic position with quintiles of scores derived from standardized weights for the first component of a principal components analysis of 22 individual household assets (47, 48).

Prevalence is summarized by frequency and percentage. We plotted prevalence of physical, sexual, and emotional violence in the last year by age group, schooling, socioeconomic asset quintile, and disability, collapsed by grouping variable and rounded using the twoway (connected) command in Stata 15. We calculated odds ratios for associations using logistic regression and Stata survey commands with cluster as the primary sampling unit and phase as stratum. We calculated intracluster correlation coefficients (ICC) for prevalence of physical, sexual, and emotional violence in the last year using the loneway command in Stata 15. The paper was prepared according to the STROBE Statement for reports of observational studies (www.strobe-statement.org).

Ethical considerations

We took permission for the survey from cluster gatekeepers identified by residents (49, 50), and followed WHO guidelines for research on domestic violence against women (51), and on sexual violence (52). Participants gave signed consent after discussing a participant information sheet. We made provision for storage of participant information sheets on women's behalf if they were concerned that the paperwork might be seen by others.

Data collectors explained their right to not provide answers to specific questions, to terminate the interview, and to withdraw temporarily or permanently without penalty. All team members were trained in Good Clinical Practice for research ethics and participant protection (Scientia Clinical Services, 14th February 2019), and interviewers were supported by three field supervisors with direct linkage to counselling services.

Interviewing women about their possible experience of violence raises issues of consent, interviewer behaviour, privacy, and confidentiality. Of particular concern is the duty of care after disclosure. We believe that an interviewee who discloses experience of violence should be offered optimal support, and interviewers were members of a broader team who were able to provide a full suite of crisis and counselling services, including home visits, medical, surgical, and psychiatric referral, and negotiation with families, the police, and legal representatives. When survivors disclosed violence, we followed established intervention

For peer review only - http://bmjopen.bmj.com/site/about/guidelines.xhtml

protocols which included safety assessment, counselling, liaison with healthcare, police, and legal services, and developing follow-up plans for the survivor and her family. Participants were able to speak with counsellors immediately by phone. When a survivor was not ready to disclose violence, the interviewer provided her with information on available services and legal rights and gave her a small card that was easy to hide and listed essential contact numbers and addresses for 24-hour crisis support, medical emergencies, and the police. She took consent for any action from the participant herself.

The trial and associated data collection were approved by the Institutional Ethics Committee of Partners for Urban Knowledge, Action, and Research (PUKAR) (25th December 2017), and the University College London Research Ethics Committee (3546/003, 27th September 2017). The TARA trial within which data collection took place is registered with the Controlled Trials Registry of India (CTRI/2018/02/012047, 21st February 2018) and with ISRCTN84502355 (22nd February 2018: http://www.isrctn.com/ISRCTN84502355).

Patient and public involvement

Community members have been involved in the design of our research since 2000. It arises from the need to provide and improve services for survivors of violence, and a commitment to working with communities to prevent it. Research questions and outcome measures were derived from international efforts, but client priorities were reflected in some specific questions that we asked as a result of client experiences. All questions were discussed with participants in pilot exercises in order to understand their perceptions and ability to respond. Community guardians were involved in recruitment and the practicalities of administering the survey. Respondents took about an hour to answer the questions, and participated only after consideration. The general findings will be disseminated through community groups involved in ongoing interventions in the study areas.

Results

Participants

Between 5th December 2017 and 28th March 2019, 5277 households were approached for the survey. 423 (2%) had no eligible woman resident and in 544 (3%) an eligible woman was unavailable after three visits. Interviewers were unable to achieve privacy in 592 cases (11%) and 155 (3%) potential respondents declined interview. The final survey included 5122 respondents. A median 101 interviews were achieved in each cluster (interquartile range 100-103; range 94-118).

Table 2 summarises characteristics of households, respondents, and their partners. Dwellings were generally of robust or mixed robust and insubstantial construction (93%). Only 17% had their own toilet. Respondents were predominantly of Hindu (59%) or Muslim (37%) faith and 58% described themselves as of general caste. 68% of women had been born outside Mumbai, 96% had been married or partnered, 2% had separated or divorced, and 2% had been widowed. 36% had married before the age of 18 years, 88% of them by arrangement, 14% had had a pregnancy before the age of 18 years, and 36% had three children or more. Women were a mean 32 years old (SD 7.3) and their partners a mean 36 (SD 8.1). 18% of women had had no schooling, compared with 10% of partners, and 36% had completed high school, compared with 48% of partners. Only 24% of women had undertaken paid work in the last year, compared with 98% of men. For these women, the main types of employment were home-based (62%), informal or formal services (17%), and domestic labour (10%). The main types of employment for men were in informal or formal services (49%) or as drivers (18%).

Domestic physical violence

Table 3 presents lifetime and 12-month prevalence of domestic violence. It includes individual questions and composite estimates for physical (9 questions), sexual (4 questions), and emotional violence (5 questions). 25% of women had ever experienced

For peer review only

domestic physical violence and 13% had experienced it in the last year. This was usually intimate partner violence (11%), but 2% had experienced physical violence from their marital family and 2% from their natal family. Around 4% of women had been kicked, dragged, or beaten in the last year, and around 3% had been threatened or attacked with a blunt or sharp object.

Domestic sexual violence

6% of women had ever experienced domestic sexual violence and 4% had experienced it in the last year, almost all by their intimate partner. Removing the question about coercion to replicate pornography from the composite estimate reduced the estimated prevalence of sexual violence by 0.1% (domestic sexual violence to 5.4% ever (274 reports) and 3.5% in the last year (179), and intimate partner sexual violence to 5.1% ever (262) and 3.5% in the last year (177). Additional questions suggested that 2.5% of partners had insisted on repeated intercourse (126 women in the last year), that 1.1% of wives had been forced to view pornography (56), that 0.8% had been subjected to coercive sex without contraception (39), and that 1.0% of partners were reported as withholding sexual pleasure from their wives (49). The prevalence of domestic physical or sexual violence in the last 12 months was 14% for currently married women, but 29% for women who had separated from their partner.

Domestic emotional violence

19% of women had experienced emotional violence in the last year. The source was roughly evenly split between intimate partner (12%) and marital family members (11%). Emotional violence came from both intimate partner and marital family for 5% of women. 6% of women had been accused of infidelity, 6% had been insulted for being a woman, and 8% of 505 women with disability had been insulted for it.

Summary figures

Limiting the dataset to 4913 ever-married women for comparison with surveys such as the NFHS, the prevalence of domestic physical violence in the last 12 months was 13%, of sexual violence 4%, of physical or sexual violence 14%, and of emotional violence 19%. Recent guidance suggests that studies present the proportion of ever-partnered women and girls aged 15 years and older who have survived physical, sexual, or psychological violence by an intimate partner in the last year (53). For intimate partner violence (a subgroup within domestic violence) our findings were 11% for physical violence, 4% for sexual, 13% for emotional (psychological), 13% for physical or sexual, and 17% for physical, sexual, or emotional violence.

Figure 2 shows prevalence of physical, sexual, and emotional violence for each of 50 clusters. The ranges were substantial: for physical violence 2-27% with interquartile range (IQR) 12-16%, for sexual violence 0-8% (IQR 2-5%), and for emotional violence 7-32% (IQR 15-23%). Intracluster correlation coefficients (ICC) were 0.023 for domestic physical violence in the last year (95% CI 0.011, 0.036), 0.005 for sexual violence (0.000, 0.010), and 0.016 for emotional violence ICC (0.006, 0.026).

Figure 3 summarises prevalence of physical, sexual, and emotional violence by age group, schooling, socioeconomic asset quintile, and disability. Women were less likely to face physical and emotional violence as they aged: the prevalence of physical violence halved between the twenties and the forties. There was no obvious pattern for schooling. All three forms of violence were more common against women in the lowest socioeconomic asset quintile and against women with disability. The odds ratios in Figure 3 did not change substantially when all four covariates were included in multivariable models, apart from for disability, which was associated with an adjusted odds ratio of 1.68 (95% confidence interval 1.24, 2.29) for physical violence, 2.18 (1.37, 3.47) for sexual violence, and 1.66 (1.31, 2.12) for emotional violence.

For peer review only - http://bmjopen.bmj.com/site/about/guidelines.xhtml

Domestic coercive control

Table 4 summarises forms and sources of coercive control. We took a conservative approach to classifying controlling behaviour. A restrictive behaviour was registered only if it was reported as always occurring, and a permissive behaviour only if it was reported as never occurring. Overall, 1816 women reported being subject to at least one controlling behavior in the list in Table 4 (35%), although we recorded responses to a larger set of questions. Respondents commonly reported that their mobility and socializing were restricted: 26% said that they always required permission to go out, 14% that they were never allowed out in the evening, and 10% that they were always accompanied when they did go out. 48% were never allowed to meet with male friends, 10% were never allowed to meet with female friends, and 5% were never allowed to meet their natal family. At home, 33% of women said that they were afraid of family members. 13% were never free to speak on the phone and 10% were never able to speak freely in the home. These kinds of restrictions were described as coming more often from marital family members than from partners. In terms of sexual and reproductive health, 52 women had been denied access to contraception and 15 had been coerced to use it. 37 (1% of 4577) had been prevented from having a termination of pregnancy, while 15 (0.3%) had been coerced to have one.

Domestic neglect

7% of women reported that their family had neglected their food in the last year, 8% that they had neglected their sleep, 8% that they had neglected their health, and 12% of 4375 with children that they had neglected their children's health. Collectively, 10% of women reported at least one of these four dimensions of neglect.

Disclosure of violence

Of 1153 women who had survived physical, sexual, or emotional domestic violence in the last year, 47% had disclosed to someone. The main confidants were family and friends (40%) and teachers, faith leaders, or local leaders (4%). Even though 18% had sought medical help, only 8% had disclosed to a healthcare provider, social worker, or counsellor. Twenty women approached our services for support within two months of the survey.

Discussion

Key findings

Our cross-sectional survey of 5122 women aged 15-49 years in informal settlements in Mumbai documented high rates of domestic violence. In the preceding year, 13% of women had suffered physical violence, 4% sexual violence, and 19% emotional violence. Physical and sexual violence was perpetrated mainly by intimate partners, but emotional violence was perpetrated equally by intimate partners and marital family members. Controlling behaviours were ascribed to both intimate partners and marital family members, with 35% of women reporting at least one of 12 restrictions.

Limitations

Our findings have three general limitations. First, although the prevalence of domestic violence was high, we should assume some underreporting given the constellation of forces inhibiting disclosure. Second, we preferentially recruited younger, married, and disabled women within households, which would tend to increase reported prevalence. Third, our study was cross-sectional and subject to the usual caveats around causal inference and the accuracy of time recall.

Comparison with other studies

We updated the review of quantitative studies by Kalokhe and colleagues (31), identifying publications from 1st January 2015 to 1st November 2019 using similar search terms, and found eight more recent published studies. The reported prevalence of domestic violence was higher in rural (54-56) than in urban settings. The estimated lifetime prevalence of

For peer review only - http://bmjopen.bmj.com/site/about/guidelines.xhtml

physical violence ranged from 18% (57) to 27% (58, 59), of sexual violence from 10% (57) to 26% (59), and of emotional violence from 20% (57) to 43% (this high estimate included questions on economic violence)(59). A cross-sectional survey similar to ours, of 1137 mothers aged 18-39 years in an informal settlement in Mumbai, found a lifetime prevalence of physical intimate partner violence of 17%, of emotional violence of 12%, and of sexual violence of 5% (60).

Our findings also compare with the NFHS-4 for Maharashtra state, in which estimates of prevalence of intimate partner violence were based on interviews with 2472 ever-married women aged 15-49 years (61). Both surveys estimated lifetime physical violence at 21%. The NFHS-4 estimate for the last 12 months was 15% (our study 11%). We found higher prevalence of sexual (5% ever compared with 2% in the NFHS-4; 4% in the last 12 months compared with 2%) and emotional violence (17% ever compared with 10% in the NFHS-4; 12% in the last 12 months compared with 7%). Our estimates of prevalence of all three types of violence were higher than those of the urban sample in the Maharashtra NFHS-4, which interviewed 1220 women (lifetime intimate partner physical violence 21% in our study, compared with 16% in the NFHS-4, sexual violence 5% compared with 2%, and emotional violence 17% compared with 9%). There are three possible explanations for our higher estimates. First, they might be based on different sets of questions: we used similar questions and composites of them and do not think this was an issue. Second, they might reflect true population differences: our participants were all residents of informal settlements and might be more likely to face violence than the urban average. Third, women might have been more likely to disclose violence to our team of interviewers, who were backed up by a strong programme of support for survivors. It is also possible that women answer questions differently when they are responding to a survey on violence than when the questions on violence are included in a broad survey like the NFHS-4. In a consideration of methodological issues in research on intimate partner violence, Saltzman suggests that questionnaire context and cues given to the participant can influence response (10).

Implications

We have a clear picture of the high burden of domestic violence against women, and of the range of findings across urban and rural settings in India. The odds of physical violence are greater for younger married women, with poorer socioeconomic position, and where underage marriage is common (62). We found that 47% of women who had suffered violence had sought help from somebody. This is considerably more than the NFHS-4, which found that 14% of urban women who had experienced physical or sexual violence had sought help from any source, usually natal family, marital family, friend or neighbor. Although disclosure to a healthcare provider, social worker, or counsellor was uncommon (8%), it was also higher than the NFHS-4 urban figure of 3%. Perhaps this provides some hope. Awareness of domestic violence is certainly increasing and it may be that women are becoming more confident that what they are going through is violence and that it is not acceptable.

Our particular concerns are perpetration of violence by the marital family and the burden of emotional violence and coercive control. Although only 11% of studies in the recent review considered non-partner violence (31), domestic violence by people other than intimate partners has long been a concern in the Indian context (63). Women entering the marital household are traditionally subordinate and the spectrum of violence extends from subtle neglect to dowry death. Toleration of their subservience in exchange for security and later authority over their own daughters-in-law has been described as the patriarchal bargain (64), and the burden of violence against women by mothers-in-law (65) and other family members has been termed gender-based household maltreatment. It informed the design of our own questionnaire (66) and includes control over reproduction, limiting contact with friends and family, and access to food (32, 34). These kinds of less visible emotional violence and control have been less well studied, but they need to be: marital family members were the

For peer review only - http://bmjopen.bmj.com/site/about/guidelines.xhtml

source of emotional violence in about half of reports in our study and of 35% of domestic violence in rural Tamil Nadu (54).

Recent work on conceptualising and measuring psychological violence (here termed emotional violence) has suggested that it is distinct from controlling behaviour (67). More than one-third of women in our study reported experiencing one of 12 controlling behaviors and other studies have reported high levels of control by intimate partners: 72% in rural Tamil Nadu (55), and 60% in rural Rajasthan (56), 43% in urban Delhi (57), and 12% in Karnataka (68). In a survey of married women in Uttar Pradesh, 12% had experienced reproductive control from husbands or in-laws, with a roughly equal split between them (69).

Although emotional violence and coercive control are less visible than physical violence, the gravity of their effects on women's mental health is being increasingly appreciated (70). Follingstad identified six types of emotional abuse that survivors of violence in the United States had experienced: verbal attacks (ridicule, name-calling, humiliation in public), isolation (social or financial), jealousy and possessiveness (including accusations of infidelity), threats of harm, threats of divorce or abandonment, and destruction of personal property. All had strong negative effects on women and 73% said that emotional violence had greater impact than physical violence (71). This finding is supported by both qualitative (71, 72) and quantitative research in high-income countries (73). In an analysis of the US National Violence Against Women Survey, psychological violence was more strongly associated with depressive symptoms than physical violence, and abuse of power and control were more strongly associated with depressive symptoms than verbal abuse (74).

We are seeing similar evidence from India. In rural Rajasthan, Richardson and colleagues "… found evidence that psychological abuse and controlling behavior were more damaging to mental health than physical abuse" (56). A cross-sectional survey of 9938 mothers aged 15-49 years in seven urban and rural locations, of whom 3155 lived in urban informal settlements, suggested that severe harassment by in-laws was associated with poor mental health (75). An analysis of follow-up data from 6303 rural married women aged 15-49 years in Bihar, Jharkhand, Maharashtra, and Tamil Nadu showed a strong association between 'verbal' violence and mental health and a much weaker association with physical violence (76).

Generalisability

While domestic violence is a global experience, there are differences in cultural experiences. A feature of the discussion in high-income countries from the 1970s onward was the disappointment of professional providers of support when a survivor of intimate partner violence did not leave the perpetrator (23, 24, 77). The emphasis in India – on the part of survivors and professionals – has been on interventions that allow families to remain together. Tolerating domestic violence has been a normalized element of women's reproductive labour (78, 79), and the ability to bear it and still fulfil the expectations of a wife and mother has been seen as praiseworthy (80, 81) and characteristic of the 'real' or 'good' woman (82). Even when domestic violence spills out into community knowledge, women who break with this tradition risk social ostracism (83), and their perceptions of their options are limited (27, 65)

Conclusion

Domestic violence against women continues to be common in urban informal settlements in Mumbai and we see no reason to doubt the external validity of our findings for other locations. Physical and sexual violence is perpetrated mainly by intimate partners, but emotional violence against women is attributed equally to partners and marital family. More than one-third of women describe at least one of a limited set of controlling behaviours, perpetrated more by intimate partners, but often by marital family members.

We have two recommendations for research and action. First, social workers and healthcare providers should be aware of the importance of emotional violence and coercive control.

For peer review only - http://bmjopen.bmj.com/site/about/guidelines.xhtml

Both are common and cause substantial suffering, particularly to women's mental health. These forms of violence need to be considered in interactions with women because of their subtlety and their intersection with poverty in urban informal settlements. Researchers should make sure that they are included in studies of domestic violence.

Second, violence is often perpetrated by family members other than intimate partners. Again, this is particularly true of emotional violence and coercive control. In interacting with clients, social workers and healthcare providers need to be aware that family members accompanying them might be involved in abuse, and studies should assume the possibility of other perpetrators.

Acknowledgments

Our greatest thanks go to the women and community guardians who agreed to contribute to the study. We thank the team of investigators who collected data in challenging conditions, Miheeka Vast and Manju Singh for supervising the field investigation team, Unnati Machchhar and Shilpa Adelkar for their supervision of the intervention programme, Gauri Savkur for contributing to investigator training, Bhaskar Kakad for investigator support, Archana Bagra and Vibhavari Bali for financial and human resources management, and Vanessa D'Souza and Shanti Pantvaidya for leadership at SNEHA.

List of figures and tables

Figure 1. Forms of domestic violence against women

Figure 2. Prevalence of physical, sexual, and emotional violence against 5122 women aged 18-49 years in 50 informal settlement clusters in Mumbai

Figure 3. Prevalence of physical, sexual, and emotional violence against 5122 women aged 18-49 years, by age group, schooling, socioeconomic asset quintile, and disability

Table 1. Comparison of questions in five surveys describing physical, sexual, and emotional violence

Table 2. Characteristics of households, respondents, and their partners

Table 3. Lifetime and 12-month prevalence of domestic physical, sexual, and emotional violence against 5122 women aged 18-49 years, by perpetrator: intimate partner, marital family, or natal family member

Table 4. Lifetime prevalence of domestic control of 5122 women aged 18-49 years, by perpetrator: intimate partner, marital family, or natal family member

Contributorship

ND and DO conceived the study and acquired funding. ND, AG, SK, LG, and DO developed the methodology, ND, SK, and AG oversaw investigation, and SK and DO curated the data. DO did the first analysis and visualization and wrote the first draft. ND managed the project with support from SK. All authors critically reviewed drafts of the manuscript and read and commented on the final version.

Competing interests

The authors declare no competing interest.

Funding

Wellcome Trust 206417. The funder had no role in study design, data collection and analysis, decision to publish, or preparation of the manuscript.

Data sharing

Data are available in Open Science Framework: *Osrin, D. (2020, June 30). Prevalence survey. Retrieved from osf.io/khdcm*

For peer review only

References

1. Ellsberg M, Jansen H, Heise L, Watts C, Garcia-Moreno C, on behalf of the WHO Multi-country Study on Women's Health and Domestic Violence against Women Study Team. Intimate partner violence and women's physical and mental health in the WHO multi-country study on women's health and domestic violence: an observational study. Lancet. 2008;371:1165-72.

2. Stöckl H, Devries K, Rotstein A, Abrahams N, Campbell J, Watts C, et al. The global prevalence of intimate partner homicide: a systematic review. Lancet. 2013;382:859-65.
3. Satyanarayana VA, Chandra PS, Vaddiparti K. Mental health consequences of violence against women and girls. Curr Opin Psychiatry. 2015;28:350-6.
4. WHO. Global and regional estimates of violence against women: prevalence and health effects of intimate partner violence and non-partner sexual violence. Geneva: World Health Organization; 2013. Report No.: 9789241564625.
5. Garcia-Moreno C, Zimmerman C, Morris-Gehring A, Heise L, Amin A, Abrahams N, et al. Addressing violence against women: a call to action. Lancet. 2015;385:1685-95.
6. Solotaroff JL, Pande RP. Violence against women and girls: lessons from South Asia. Washington DC; 2014.
7. UN General Assembly. Declaration on elimination of violence against women. GA res. 48/104, 48 UN GAOR. Supp. No. 49 at 217, UN Doc. A/48/49. www1.umn.edu/humanrts/instree/e4devw.htm; 1993.
8. UNDAW. Indicators to measure violence against women. Report of the Expert Group Meeting. Geneva: United Nations Division for the Advancement of Women, United Nations Economic Commission for Europe, United Nations Statistical Division; 2007.
9. Gelles RJ. Estimating the incidence and prevalence of violence against women. Violence Against Women. 2000;6:784-804.
10. Saltzman LE. Definitional and methodological issues related to transnational research on intimate partner violence. Violence Against Women. 2004;10:812-30.
11. Kilpatrick DG. What is violence against women? Defining and measuring the problem. J Interpers Violence. 2004;19:1209-34.
12. Heise L. Violence against women: an integrated ecological framework. Violence Against Women. 1998;4:262-90.
13. Piedalue A. Understanding violence in place: travelling knowledge paradigms and measuring domestic violence in India. Ind J Gender Stud. 2015;22.
14. Garcia-Moreno C, Jansen HA, Ellsberg M, Heise L, Watts C. WHO multi-country study on women's health and domestic violence against women: initial results on prevalence, health outcomes and women's responses. Geneva: World Health Organization; 2005.
15. Johnson H, Ollus N, Nevala S. Violence against women: an international perspective. New York NY: European Institute for Crime Prevention and Control (HEUNI); 2008.
16. Waltermaurer E. Measuring intimate partner violence (IPV): you may only get what you ask for. J Interpers Violence. 2005;20:501-6.
17. Thompson MP, Basile KC, Hertz MF, Sitterle D. Measuring intimate partner violence victimization and perpetration: a compendium of assessment tools. Atlanta GA: Centers for Disease Control and Prevention, National Center for Injury Prevention and Control; 2006.
18. Ruiz-Pérez I, Plazaola-Castaño J, Vives-Cases C. Methodological issues in the study of violence against women. J Epidemiol Commun Health. 2007;61:ii26-ii31.
19. Bagwell-Gray ME, Messing JT, Baldwin-White A. Intimate partner sexual violence: a review of terms, definitions, and prevalence. Trauma Violence Abuse. 2015;16:316-35.

For peer review only - http://bmjopen.bmj.com/site/about/guidelines.xhtml

20. Straus M. Measuring intrafamily conflict and violence: the Conflict Tactics (CT) Scales. J Marriage Family. 1979;41:75-88.
21. Straus M, Hamby S, Boney-McCoy S, Sugarman D. The Revised Conflict Tactics Scales (CTS2) development and preliminary psychometric data. J Family Issues. 1996;17:283-316.
22. Tjaden P. Defining and measuring violence against women: background, issues, and recommendations. Geneva: UN Division for the Advancement of Women, Economic Commission for Europe, World Health Organization; 2005.
23. Ferraro KJ, Johnson JM. How women experience battering: the process of victimization. Social Problems. 1983;30:325-39.
24. Mills T. The assault on the self: stages in coping with battering husbands. Qualitative Sociology. 1985;8:103-23.
25. Decker MR, Nair S, Saggurti N, Sabri B, Jethva M, Raj A, et al. Violence-related coping, help-seeking and health care-based intervention preferences among perinatal women in Mumbai, India. J Interpers Violence. 2013;28:1924-47.
26. Jejeebhoy S, Santhya KG, Acharya R. Violence against women in South Asia: the need for the active engagement of the health sector. Global Public Health. 2014;9:678-90.
27. Ragavan M, Iyengar K, Wurtz R. Perceptions of options available for victims of physical intimate partner violence in northern India. Violence Against Women. 2015;21:652-75.
28. Leonardsson M, San Sebastion M. Prevalence and predictors of help-seeking for women exposed to spousal violence in India - a cross-sectional study. BMC Women's Health. 2017;17:99.
29. Vranda MN, Kumar CN, Muralidhar D, Janardhana N, Sivakumar PT. Barriers to disclosure of intimate partner violence among female patients availing services at tertiary care psychiatric hospitals: a qualitative study. J Neurosci Rural Pract. 2018;9:326-30.
30. United Nations. Convention on the elimination of all forms of discrimination against women. http://www.un.org/womenwatch/daw/cedaw/. New York: United Nations; 1979.
31. Kalokhe A, del Rio C, Dunkle K, Stephenson R, Metheny N, Paranjape A, et al. Domestic violence against women in India: A systematic review of a decade of quantitative studies. Global Public Health. 2016:1-16.
32. Kalokhe AS, Potdar RR, Stephenson R, Dunkle KL, Paranjape A, del rio C, et al. How well does the World Health Organization definition of domestic violence work for India? PLoS ONE. 2015;10:e0120909.
33. Silverman JG, Donta B, Decker MR, Boyce SC, Ritter J, Naik DD, et al. Family violence and maltreatment of women during the perinatal period: associations with infant morbidity in Indian slum communities. Matern Child Health J. 2016;20:149-57.
34. Kalokhe AS, Stephenson R, Kelley ME, Dunkle KL, Paranjape A, Solas V, et al. The Development and Validation of the Indian Family Violence and Control Scale. PLoS One. 2016;11(1):e0148120.
35. Ruikar MM, Pratinidhi AK. Physical wife abuse in an urban slum of Pune, Maharashtra. Ind J Public Health. 2008;52:215-7.
36. Sinha A, Mallik S, Sanyal D, Dasgupta S, Pal D, Mukherjee A. Domestic violence among ever married women of reproductive age group in a slum area of Kolkata. Indian J Public Health. 2012;56(1):31-6.
37. Shrivastava PS, Shrivastava SR. A study of spousal domestic violence in an urban slum of Mumbai. Int J Prev Med. 2013;4:27-32.

For peer review only - http://bmjopen.bmj.com/site/about/guidelines.xhtml

38. Sabri B, Campbell JC. Intimate partner violence against women in slums in India. Ind J Med Res. 2015;141:757-9.
39. UNDESA. World social report 2020: inequality in a rapidly changing world. New York NY: United Nations Department of Economic and Social Affairs; 2020. Contract No.: ST/ESA/372.
40. United Nations Human Settlements Programme (UN-Habitat). The challenge of slums: global report on human settlements 2003. London and Sterling VA: Earthscan Publications Ltd; 2003.
41. Central Bureau of Health Intelligence. National health profile 2019. New Delhi: Directorate General of Health Services, Ministry of Health and Family Welfare, Government of India; 2019.
42. Chandramouli C. Housing stock, amenities and assets in slums - Census 2011. Available from www.censusindia.gov.in. New Delhi: Office of the Registrar General and Census Commissioner,; 2011.
43. Daruwalla N, Machchhar U, Pantvaidya S, D'Souza V, Gram L, Copas A, et al. Community interventions to prevent violence against women and girls in informal settlements in Mumbai: the SNEHA-TARA pragmatic cluster randomised controlled trial. Trials. 2019;20(1):743.
44. Osrin D, Das S, Bapat U, Alcock GA, Joshi W, More NS. A rapid assessment scorecard to identify informal settlements at higher maternal and child health risk in Mumbai. J Urban Health. 2011;88(5):919-32.
45. IIPS. National Family Health Survey 2015-16 (NFHS-4): interviewer's manual. Mumbai: International Institute for Population Sciences; 2014.
46. Washington Group on Disability Statistics (WG). Understanding and interpreting disability as measured using the WG Short Set of questions. Hyattsville MD; 2009.
47. Filmer D, Pritchett L. Estimating wealth effects without expenditure data - or tears: an application to educational enrollments in states of India. Demography. 2001;38:115-32.
48. Vyas S, Kumaranayake L. Constructing socio-economic status indices: how to use principal components analysis. Health Policy Plan. 2006;21:459-68.
49. Taljaard M, Weijer C, Grimshaw JM, Eccles MP. The Ottawa Statement on the ethical design and conduct of cluster randomised trials: precis for researchers and research ethics committees. BMJ. 2013;346:f2838.
50. Lignou S, Das S, Mistry J, Alcock G, More NS, Osrin D, et al. Reconstructing communities in cluster trials? Trials. 2016;17:166.
51. WHO. Putting women first: ethical and safety recommendations for research on domestic violence against women. WHO/FCH/GWH/01.1. Geneva: Department of Gender and Women's Health, Family and Community Health, World Health Organization; 2001.
52. Jewkes R, Dartnall E, Sikweyiya Y. Ethical and safety recommendations for research on perpetration of sexual violence. Pretoria: Medical Research Council, Sexual Violence Research Initiative; 2012.
53. WHO. Consensus on priority indicators on violence against women Geneva2020 [Available from: https://www.who.int/reproductivehealth/topics/violence/vaw-indicators/en/.
54. George J, Nair D, Premkumar NR, Saravanan N, Chinnakali P, Roy G. The prevalence of domestic violence and its associated factors among married women in a rural area of Puducherry, South India. J Family Med Prim Care. 2016;5(3):672-6.

For peer review only - http://bmjopen.bmj.com/site/about/guidelines.xhtml

55. Ram A, Victor CP, Christy H, Hembrom S, Cherian AG, Mohan VR. Domestic violence and its determinants among 15-49-year-old women in a rural block in South India. Ind J Commun Med. 2019;44:362-7.
56. Richardson R, Nandi A, Jaswal S, Harper S. The effect of intimate partner violence on women's mental distress: a prospective cohort study of 3010 rural Indian women. Soc Psychiatry Psychiatr Epidemiol. 2019.
57. Mukherjee R, Joshi RK. Controlling Behavior and Intimate Partner Violence: A Cross-Sectional Study in an Urban Area of Delhi, India. J Interpers Violence. 2019:886260519876720.
58. Nadda A, Malik JS, Rohilla R, Chahal S, Chayal V, Arora V. Study of domestic violence amoung currently married females of Haryana, India. Ind J Psychol Med. 2018;40:534-9.
59. Sharma KK, Vatsa M, Kalaivani M, Bhardwaj D. Mental health effects of domestic violence against women in Delhi: A community-based study. J Family Med Prim Care. 2019;8(7):2522-7.
60. Begum S, Donta B, Nair S, Prakasam CP. Socio-demographic factors associated with domestic violence in urban slums, Mumbai, Maharashtra, India Indian J Med Res. 2015;141:783-8.
61. International Institute for Population Sciences (IIPS), ICF. National Family Health Survey (NFHS-4), India, 2015-16: Maharashtra. Mumbai: IIPS; 2018.
62. Ahmad J, Khan NH, Mozumdar A. Spousal Violence Against Women in India: A Social-Ecological Analysis Using Data From the National Family Health Survey 2015 to 2016. J Interpers Violence. 2019(Oct 23):886260519881530.
63. Kumar V, Kanth S. Bride burning. Lancet. 2004;364 Suppl 1:s18-9.
64. Kandiyoti D. Bargaining with patriarchy. Gender and Society. 1988;2:274-90.
65. Ragavan M, Iyengar K. Violence perpetrated by mothers-in-law in Northern India: perceived frequency, acceptability, and options for survivors. J Interpers Violence. 2017(May 1):886260517708759.
66. Ritter J, Dasgupta A, Raj A, Decker MR, Nair S, Trivedi A, et al. Measuring gender-based household maltreatment in urban slums of Mumbai, India. J Interpers Violence. 2019;1-23(Jul 31):886260519864370.
67. Heise L, Pallitto C, García-Moreno C, Clark CJ. Measuring psychological abuse by intimate partners: constructing a cross-cultural indicator for the Sustainable Development Goals. SSM Popul Health. 2019;9:100377.
68. Kundapur R, Shetty SM, Kempaller VJ, Kumar A, Anurupa M. Violence against Educated Women by Intimate Partners in Urban Karnataka, India. Indian J Community Med. 2017;42(3):147-50.
69. Silverman JG, Boyce SC, Dehingia N, Rao N, Chandurkar D, Nanda P, et al. Reproductive coercion in Uttar Pradesh, India: Prevalence and associations with partner violence and reproductive health. SSM Popul Health. 2019;9:100484.
70. Jewkes R. Emotional abuse: a neglected dimension of partner violence. Lancet. 2010;376:851-2.
71. Follingstad DR, Rutledge LL, Berg BJ, Hause ES, Polek DS. The role of emotional abuse in physically abusive relationships. J Family Violence. 1990;5:107-20.
72. Vitanza S, Vogel LCM, Marshall LL. Distress and symptoms of posttraumatic stress disorder in abused women. Violence Victims. 1995;10:23-34.

For peer review only - http://bmjopen.bmj.com/site/about/guidelines.xhtml

73. Mechanic MB, Weaver TL, Resick PA. Mental health consequences of intimate partner abuse: a multidimensional assessment of four different forms of abuse. Violence Against Women. 2008;14:634-54.
74. Coker AL, Davis KE, Arias I, Desai S, Sanderson M, Brandt HM, et al. Physical and mental health effects of intimate partner violence for men and women. Am J Prev Med. 2002;23:260-8.
75. Kumar S, Jeyaseelan L, Suresh S, Ahuja RC. Domestic violence and its mental health correlates in Indian women. Br J Psychiatry. 2005;187:62-7.
76. Stephenson R, Winter A, Hindin M. Frequency of intimate partner violence and rural women's mental health in four Indian states. Violence Against Women. 2013;19:1133.
77. Gelles RJ. Abused wives: why do they stay. J Marriage Family. 1976;38:659-68.
78. Anitha S. Understanding economic abuse through an intersectional lens: financial abuse, control, and exploitation of women's productive and reproductive labor. Violence Against Women. 2019;25:1854-77.
79. Jejeebhoy SJ, Cook RJ. State accountability for wife-beating: the Indian challenge. Lancet. 1997;349(Suppl 1):s10-2.
80. Narula DK, Narula A. Patriarchal societies and domestic violence: need for empowering women. In: Charak P, Mohan C, editors. Women and development: self, society and empowerment. New Delhi: Primus Books; 2012. p. 169-83.
81. Ahmed-Ghosh H. Chattels of society: domestic violence in India. Violence Against Women. 2004;10:94-118.
82. Jejeebhoy SJ, Santhya KG, Sabarwal S. Gender-based violence: a qualitative exploration of norms, experiences and positive deviance. New Delhi: Population Council; 2013.
83. Ghosh S. Watching, blaming, silencing, intervening: exploring the role of the community in preventing domestic violence in India. Practicing Anthropology. 2011;33:22-6.

For peer review only

Table 1. Comparison of questions in five surveys describing physical, sexual, and emotional violence

	SNEHA TARA	NFHS-4 (45)	Indian Family Violence and Control Scale (34)	WHO multi-country study (14)	International Violence Against Women Survey (15)
**Physical**					
Pushed, shoved, shaken, hurt	√	√	√	√	√
Twisted arm, banged head, pulled hair	√	√	√		√
Slapped, pinched, bitten	√	√	√	√	√
Hit, punched	√	√	√	√	
Kicked, dragged, beaten	√	√	√	√	
Things thrown at, burned	√		√	√	
Attacked or threatened with sharp object	√	√	√	√	√
Attacked or threatened with blunt object	√	√	√	√	√
Suffocated, choked, hung, poisoned	√	√	√	√	√
**Sexual**					
Forced intercourse	√	√	√	√	√
Forced other degrading act	√	√	√	√	
Threatened other act	√	√			
Forced to replicate pornography	√		√		
Forced sex during menses	√		√		
Forced sex with someone else	√		√		
Forced sex without contraception	√		√		
Threatened sex with another person	√		√		
Forced video of sex	√		√		
Taken advantage of when drugged or drunk	√		√		
Forced to watch pornography	√				
Insisted on repeated intercourse	√				
Forced to entertain others sexually	√				
Forced drugs or alcohol for sex	√				
Partner withheld sexual pleasure	√				
**Emotional**					
Insulted, made to feel bad about herself	√	√	√	√	
Belittled, humiliated in front of others	√	√	√	√	
Ignored, treated indifferently	√		√	√	
Scared or intimidated on purpose	√		√	√	
Threated to hurt her, someone close, or take child	√	√		√	√
Insulted for not having a baby	√		√		
Insulted for not having a son	√		√		
Afraid of family members	√				
Accused of infidelity	√	√		√	
Insulted for being a woman	√				

For peer review only

Table 2. Characteristics of households, respondents, and their partners

Household	n	(%)
**Fabric**		
Kachha (insubstantial)	353	(7)
Mixed	2149	(42)
Pukka (robust)	2620	(51)
**Toilet**		
Private	875	(17)
Public or charity	4245	(83)
Open defecation	2	(0)
**Religion**		
Hindu	3002	(59)
Muslim	1902	(37)
Buddhist	158	(3)
Other	60	(1)
All	5122	(100)

Individual	Woman respondent		Husband or partner	
	n	(%)	n	(%)
**Age group**				
18-19 y	115	(2)	14	(0)
20-29 y	2038	(40)	920	(19)
30-39 y	2056	(40)	2108	(44)
40-49 y	913	(18)	1372	(29)
50+ y			391	(8)
**Education**				
None	947	(18)	474	(10)
Primary 1-5 y	859	(17)	590	(12)
Middle 6-8 y	1122	(22)	981	(20)
High 9-10 y	1158	(23)	1543	(32)
Senior 11-12 y	572	(11)	698	(15)
Undergraduate	363	(7)	371	(8)
Postgraduate	100	(2)	123	(3)
Other	1	(0)	25	(1)
**Paid work in last 12 m**				
Yes	1252	(24)	4697	(98)
No	3870	(76)	108	(2)
All	5122	(100)	4805	(100)

For peer review only

Table 3. Lifetime and 12-month prevalence of domestic physical, sexual, and emotional violence against 5122 women aged 18-49 years, by perpetrator: intimate partner, marital family, or natal family member

	Lifetime		Lifetime by perpetrator						Last 12 m		Last 12 m by perpetrator					
	Domestic		Intimate partner		Marital		Natal		Domestic		Intimate partner		Marital		Natal	
	n	(%)	n	(%)	n	(%)	n	(%)	n	(%)	n	(%)	n	(%)	n	(%)
**Physical violence**																
Pushed, shoved, shaken, hurt	595	(11.6)	458	(8.9)	186	(3.6)	33	(0.6)	292	(5.7)	247	(4.8)	70	(1.4)	17	(0.3)
Twisted arm, banged head, pulled hair	464	(9.1)	371	(7.2)	121	(2.4)	23	(0.4)	224	(4.4)	194	(3.8)	43	(0.8)	10	(0.2)
Slapped, pinched, bitten	1154	(22.5)	986	(19.3)	134	(2.6)	139	(2.7)	565	(11.0)	504	(9.8)	52	(1.0)	71	(1.4)
Hit, punched	421	(8.2)	351	(6.9)	90	(1.8)	28	(0.5)	206	(4.0)	182	(3.6)	30	(0.6)	10	(0.2)
Kicked, dragged, beaten	398	(7.8)	326	(6.4)	88	(1.7)	28	(0.5)	199	(3.9)	175	(3.4)	32	(0.6)	10	(0.2)
Things thrown at, burned	51	(1.0)	41	(0.8)	14	(0.3)	0	(0.0)	15	(0.3)	13	(0.3)	3	(0.1)	0	(0.0
Attacked or threatened with sharp object	96	(1.9)	74	(1.4)	22	(0.4)	3	(0.1)	51	(1.0)	44	(0.9)	6	(0.1)	2	(0.0)
Attacked or threatened with blunt object	218	(4.3)	165	(3.2)	51	(1.0)	22	(0.4)	103	(2.0)	90	(1.8)	16	(0.3)	6	(0.1)
Suffocated, choked, hung, poisoned	114	(2.2)	96	(1.9)	20	(0.4)	5	(0.1)	52	(1.0)	48	(0.9)	8	(0.2)	1	(0.0)
**Sexual violence**																
Forced intercourse	231	(4.5)	219	(4.3)	6	(0.1)	4	(0.1)	138	(2.7)	136	(2.7)	1	(0.0)	0	(0.0)
Forced other degrading act	128	(2.5)	126	(2.5)	1	(0.0)	1	(0.0)	95	(1.9)	95	(1.9)	1	(0.0)	0	(0.0)
Threatened other act	74	(1.4)	72	(1.4)	0	(0.0)	1	(0.0)	43	(0.8)	43	(0.8)	0	(0.0)	0	(0.0)
Forced to replicate pornography	75	(1.5)	75	(1.5)	0	(0.0)	0	(0.0)	45	(0.9)	45	(0.9)	0	(0.0)	0	(0.0)
**Emotional violence**																
Insulted, made to feel bad about herself	1296	(25.3)	536	(10.5)	877	(17.1)	99	(1.9)	690	(13.5)	347	(6.8)	419	(8.2)	64	(1.2)
Ignored, treated indifferently	773	(15.1)	339	(6.6)	541	(10.6)	50	(1.0)	449	(8.8)	230	(4.5)	290	(5.7)	34	(0.7)
Belittled, humiliated in front of others	682	(13.3)	313	(6.1)	445	(8.7)	52	(1.0)	417	(8.1)	223	(4.4)	242	(4.7)	35	(0.7)
Scared or intimidated on purpose	841	(16.4)	592	(11.6)	330	(6.4)	67	(1.3)	570	(11.1)	432	(8.4)	191	(3.7)	52	(1.0)
Threated to hurt her, someone close, or take child away	213	(4.2)	147	(2.9)	100	(2.0)	6	(0.1)	130	(2.5)	100	(2.0)	49	(1.0)	4	(0.1)
**Any physical violence**	1293	(25.2)	1084	(21.2)	261	(5.1)	170	(3.3)	644	(12.6)	562	(11.0)	103	(2.0)	84	(1.6)
**Any sexual violence**	289	(5.6)	277	(5.4)	7	(0.1)	4	(0.1)	188	(3.7)	186	(3.6)	2	(0.0)	0	(0.0)
**Any emotional violence**	1599	(31.2)	876	(17.1)	1045	(20.4)	138	(2.7)	963	(18.8)	607	(11.9)	538	(10.5)	90	(1.8)
**All**	**5122**	**(100.0)**	**5122**	**(100.0)**	**5122**	**(100.0)**	**5122**	**(100.0)**	**5122**	**(100.0)**	**5122**	**(100.0)**	**5122**	**(100.0)**	**5122**	**(100.0)**

For peer review only

Table 4. Lifetime prevalence of domestic control of 5122 women aged 18-49 years, by perpetrator: intimate partner, marital family, or natal family member

**Control**	Domestic		Intimate partner		Marital		Natal	
	n	(%)	n	(%)	n	(%)	n	(%)
**Mobility**								
Movement always monitored	419	(8.2)	278	(5.4)	140	(2.7)	44	(0.9)
Always prevented from attending meetings	227	(4.4)	129	(2.5)	118	(2.3)	5	(0.1)
**Education**								
Prevented from schooling	152	(3.0)	53	(1.0)	47	(0.9)	62	(1.2)
**Employment**								
Prevented from seeking employment	853	(16.7)	728	(14.2)	162	(3.2)	31	(0.6)
Coerced to seek employment	78	(1.5)	42	(0.8)	35	(0.7)	7	(0.1)
**Home dynamics**								
Excluded from family matters	366	(7.1)	108	(2.1)	300	(5.9)	14	(0.3)
Dress or hairstyle always controlled	267	(5.2)	119	(2.3)	145	(2.8)	25	(0.5)
Limited access to house	173	(3.4)	56	(1.1)	137	(2.7)	7	(0.1)
Forced out of house	165	(3.2)	52	(1.0)	121	(2.4)	5	(0.1)
Locked in house	39	(0.8)	18	(0.4)	22	(0.4)	1	(0.0)
**Health**								
Made to do excessive work	239	(4.7)	39	(0.8)	209	(4.1)	11	(0.2)
Always requires permission for healthcare	753	(14.7)	513	(10.0)	286	(5.6)	48	(0.9)
**Any of the above**	**1816**	**(35.4)**	**1303**	**(25.4)**	**830**	**(16.2)**	**184**	**(3.6)**
**All**	5122	(100.0)	5122	(100.0)	5122	(100.0)	5122	(100.0)

For peer review only

For peer review only - http://bmjopen.bmj.com/site/about/guidelines.xhtml

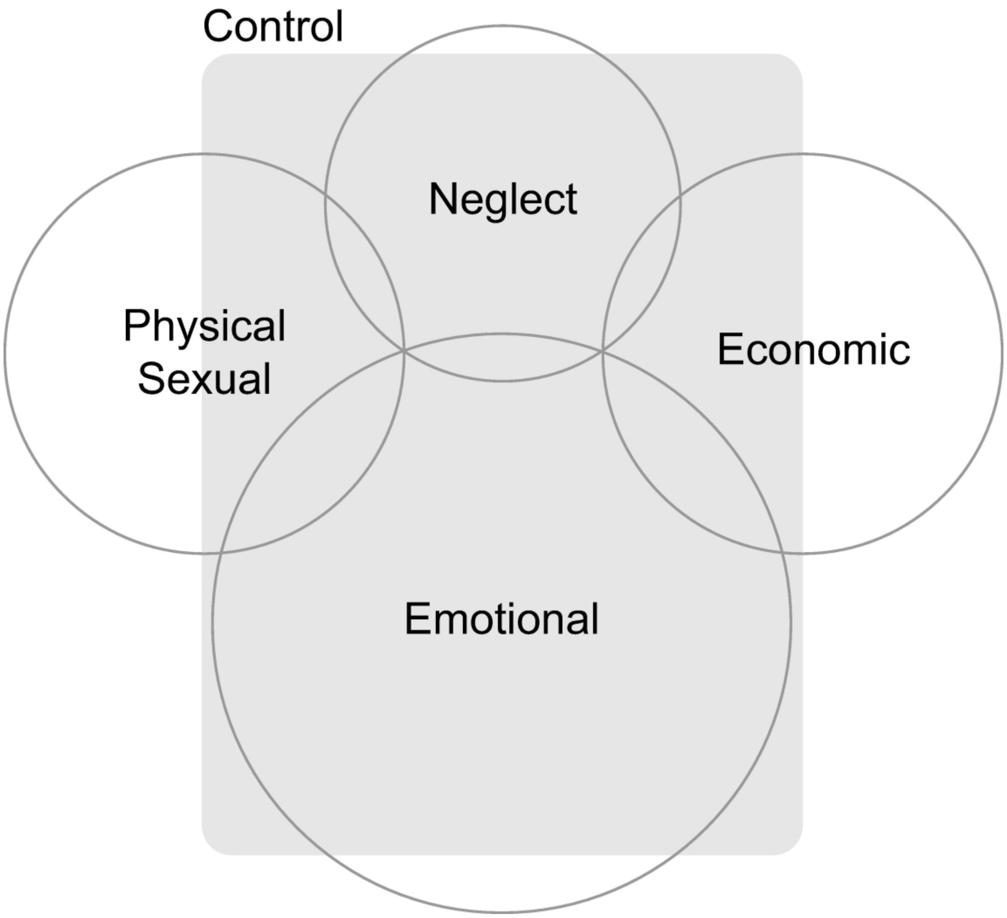

Figure 1. Forms of domestic violence against women

143x130mm (600 x 600 DPI)

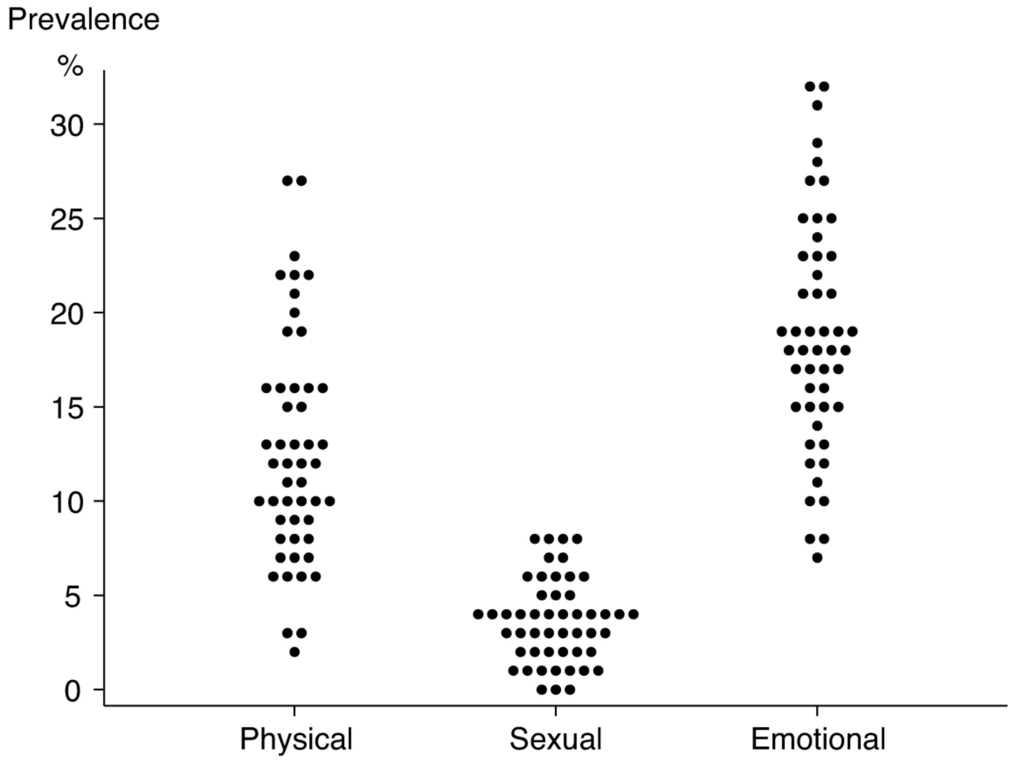

Figure 2. Prevalence of physical, sexual, and emotional violence against 5122 women aged 18-49 years in 50 informal settlement clusters in Mumbai

138x105mm (600 x 600 DPI)

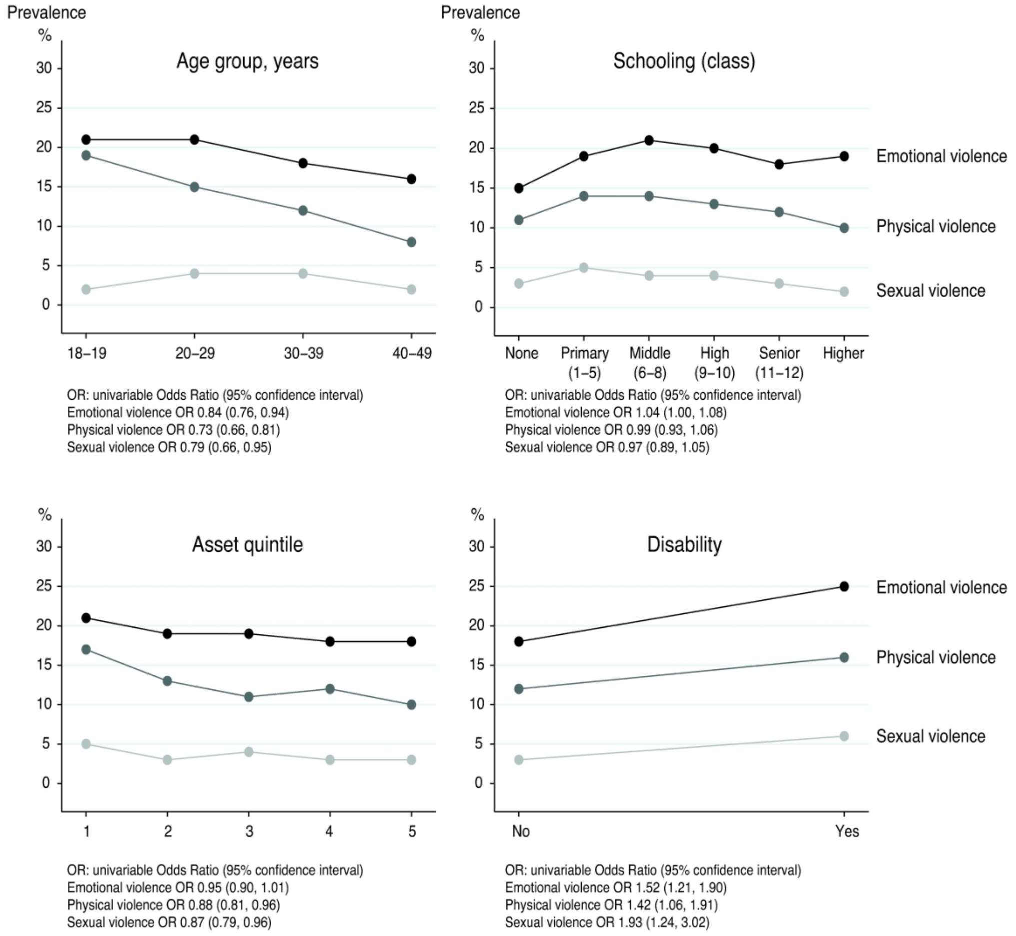

Figure 3. Prevalence of physical, sexual, and emotional violence against 5122 women aged 18-49 years, by age group, schooling, socioeconomic asset quintile, and disability

195x181mm (600 x 600 DPI)

STROBE 2007 (v4) Statement—Checklist of items that should be included in reports of *cross-sectional studies*

Section/Topic	Item #	Recommendation	Reported on page #
**Title and abstract**	1	(*a*) Indicate the study's design with a commonly used term in the title or the abstract	1
		(*b*) Provide in the abstract an informative and balanced summary of what was done and what was found	2
**Introduction**			
Background/rationale	2	Explain the scientific background and rationale for the investigation being reported	4-5
Objectives	3	State specific objectives, including any prespecified hypotheses	5
**Methods**			
Study design	4	Present key elements of study design early in the paper	5
Setting	5	Describe the setting, locations, and relevant dates, including periods of recruitment, exposure, follow-up, and data collection	5-6, 8
Participants	6	(*a*) Give the eligibility criteria, and the sources and methods of selection of participants	6
Variables	7	Clearly define all outcomes, exposures, predictors, potential confounders, and effect modifiers. Give diagnostic criteria, if applicable	6-7
Data sources/ measurement	8*	For each variable of interest, give sources of data and details of methods of assessment (measurement). Describe comparability of assessment methods if there is more than one group	7
Bias	9	Describe any efforts to address potential sources of bias	6
Study size	10	Explain how the study size was arrived at	5
Quantitative variables	11	Explain how quantitative variables were handled in the analyses. If applicable, describe which groupings were chosen and why	6-7
Statistical methods	12	(*a*) Describe all statistical methods, including those used to control for confounding	6-7
		(*b*) Describe any methods used to examine subgroups and interactions	7
		(*c*) Explain how missing data were addressed	7
		(*d*) If applicable, describe analytical methods taking account of sampling strategy	7
		(*e*) Describe any sensitivity analyses	
**Results**			

For peer review only

Participants	13*	(a) Report numbers of individuals at each stage of study—eg numbers potentially eligible, examined for eligibility, confirmed eligible, included in the study, completing follow-up, and analysed	8
		(b) Give reasons for non-participation at each stage	8
		(c) Consider use of a flow diagram	
Descriptive data	14*	(a) Give characteristics of study participants (eg demographic, clinical, social) and information on exposures and potential confounders	8 (Table 2)
		(b) Indicate number of participants with missing data for each variable of interest	8 (Table 2)
Outcome data	15*	Report numbers of outcome events or summary measures	8-10 (Tables 3, 4)
Main results	16	(*a*) Give unadjusted estimates and, if applicable, confounder-adjusted estimates and their precision (eg, 95% confidence interval). Make clear which confounders were adjusted for and why they were included	9
		(*b*) Report category boundaries when continuous variables were categorized	
		(*c*) If relevant, consider translating estimates of relative risk into absolute risk for a meaningful time period	
Other analyses	17	Report other analyses done—eg analyses of subgroups and interactions, and sensitivity analyses	
**Discussion**			
Key results	18	Summarise key results with reference to study objectives	10
Limitations	19	Discuss limitations of the study, taking into account sources of potential bias or imprecision. Discuss both direction and magnitude of any potential bias	10
Interpretation	20	Give a cautious overall interpretation of results considering objectives, limitations, multiplicity of analyses, results from similar studies, and other relevant evidence	11-12
Generalisability	21	Discuss the generalisability (external validity) of the study results	12
**Other information**			
Funding	22	Give the source of funding and the role of the funders for the present study and, if applicable, for the original study on which the present article is based	13

*Give information separately for cases and controls in case-control studies and, if applicable, for exposed and unexposed groups in cohort and cross-sectional studies.

Note: An Explanation and Elaboration article discusses each checklist item and gives methodological background and published examples of transparent reporting. The STROBE checklist is best used in conjunction with this article (freely available on the Web sites of PLoS Medicine at http://www.plosmedicine.org/, Annals of Internal Medicine at http://www.annals.org/, and Epidemiology at http://www.epidem.com/). Information on the STROBE Initiative is available at www.strobe-statement.org.

For peer review only

BMJ Open

Prevalence of domestic violence against women in informal settlements in Mumbai, India: a cross-sectional survey

Journal:	*BMJ Open*
Manuscript ID	bmjopen-2020-042444.R2
Article Type:	Original research
Date Submitted by the Author:	09-Nov-2020
Complete List of Authors:	Daruwalla, Nayreen; Society for Nutrition, Education & Health Action (SNEHA)<br>Kanougiya, Suman; Society for Nutrition, Education & Health Action (SNEHA)<br>Gupta, Apoorwa; Society for Nutrition, Education & Health Action (SNEHA)<br>Gram, Lu; University College London, Institute for Global Health<br>Osrin, David; University College London, Institute for Global Health
**Primary Subject Heading**:	Public health
Secondary Subject Heading:	Epidemiology, Global health
Keywords:	PUBLIC HEALTH, PRIMARY CARE, EPIDEMIOLOGY

SCHOLARONE™
Manuscripts

I, the Submitting Author has the right to grant and does grant on behalf of all authors of the Work (as defined in the below author licence), an exclusive licence and/or a non-exclusive licence for contributions from authors who are: i) UK Crown employees; ii) where BMJ has agreed a CC-BY licence shall apply, and/or iii) in accordance with the terms applicable for US Federal Government officers or employees acting as part of their official duties; on a worldwide, perpetual, irrevocable, royalty-free basis to BMJ Publishing Group Ltd ("BMJ") its licensees and where the relevant Journal is co-owned by BMJ to the co-owners of the Journal, to publish the Work in this journal and any other BMJ products and to exploit all rights, as set out in our licence.

The Submitting Author accepts and understands that any supply made under these terms is made by BMJ to the Submitting Author unless you are acting as an employee on behalf of your employer or a postgraduate student of an affiliated institution which is paying any applicable article publishing charge ("APC") for Open Access articles. Where the Submitting Author wishes to make the Work available on an Open Access basis (and intends to pay the relevant APC), the terms of reuse of such Open Access shall be governed by a Creative Commons licence – details of these licences and which Creative Commons licence will apply to this Work are set out in our licence referred to above.

Other than as permitted in any relevant BMJ Author's Self Archiving Policies, I confirm this Work has not been accepted for publication elsewhere, is not being considered for publication elsewhere and does not duplicate material already published. I confirm all authors consent to publication of this Work and authorise the granting of this licence.

For peer review only

Prevalence of domestic violence against women in informal settlements in Mumbai, India: a cross-sectional survey

Authors

Nayreen Daruwalla

Program on Prevention of Violence Against Women and Children, SNEHA, Mumbai, Maharashtra, 400017, India

nayreen@snehamumbai.org

Suman Kanougiya

Program on Prevention of Violence Against Women and Children, SNEHA, Mumbai, Maharashtra, 400017, India

suman.kanougiya@gmail.com

Apoorwa Gupta

Program on Prevention of Violence Against Women and Children, SNEHA, Mumbai, Maharashtra, 400017, India

apoorwa.gupta@snehamumbai.org

Lu Gram

Institute for Global Health, University College London, London, WC1N IEH, UK

lu.gram.13@ucl.ac.uk

David Osrin (corresponding author)

Institute for Global Health, University College London, London, WC1N IEH, UK

d.osrin@ucl.ac.uk

For peer review only

Abstract

Objectives

Domestic violence against women harms individuals, families, communities, and society. Perpetrated by intimate partners or other family members, its overlapping forms include physical, sexual, and emotional violence, control and neglect. We aimed to describe the prevalence of these forms of violence and their perpetrators in informal settlements in Mumbai.

Design

Cross-sectional survey.

Setting

Two large urban informal settlement areas.

Participants

5122 women aged 18-49 years.

Primary and secondary outcome measures

Prevalence and perpetrators in the last year of physical, sexual, and emotional domestic violence, coercive control and neglect. For each of these forms of violence, responses to questions about individual acts and composite estimates.

Results

In the last year, 644 (13%) women had experienced physical domestic violence, 188 (4%) sexual violence, and 963 (19%) emotional violence. Of ever-married women, 13% had experienced physical or sexual intimate partner violence in the last year. Most physical (87%) and sexual violence (99%) was done by partners, but emotional violence equally involved marital family members. All three forms of violence were more common if women were younger, in the lowest socioeconomic asset quintile, or reported disability. 1816 women (35%) had experienced at least one instance of coercive control and 33% said that they were afraid of people in their home. 10% reported domestic neglect of their food, sleep, health, or children’s health.

Conclusions

Domestic violence against women remains common in urban informal settlements. Physical and sexual violence was perpetrated mainly by intimate partners, but emotional violence was attributed equally to partners and marital family. More than one-third of women described controlling behaviours perpetrated by both intimate partners and marital family members. We emphasise the need to include the spectrum of perpetrators and forms of domestic violence – particularly emotional violence and coercive control - in data gathering.

Key words

Domestic Violence; Gender-based Violence; Intimate Partner Violence; Mumbai; India

For peer review only

Article summary

Strengths and limitations of this study

- A large cross-sectional survey of women in informal settlements.
- The study included emotional violence, coercive control, and neglect, as well as physical and sexual domestic violence against women.
- The study considered perpetrators of violence other than intimate partners.
- The cross-sectional nature of the study limited the possibility of causal inference.

For peer review only

For peer review only - http://bmjopen.bmj.com/site/about/guidelines.xhtml

Background

The global burden of domestic violence is underlined by its inclusion as an indicator of gender equality for the fifth United Nations Sustainable Development Goal (https://sdg-tracker.org/gender-equality). Domestic violence against women harms individuals, families, communities, and society. For individuals, it causes injury or death (1, 2), reproductive health problems, harmful drug and alcohol use, anxiety, depression, post-traumatic stress disorder, self-harm, and suicide (1, 3). For families, it leads to miscarriage, induced abortion, stillbirth, low birth weight, preterm delivery, and further violence (4, 5). For communities and society, it leads to lack of agency, limited participation, and lost economic productivity (5, 6).

Globally, 30% of women have survived physical or sexual violence by an intimate partner or sexual violence by a non-partner (4). The precision of the wording of this statement illustrates the challenge of defining violence against women. The United Nations Declaration on the Elimination of Violence against Women defines it as ‘‘any act of gender-based violence that results in, or is likely to result in, physical, sexual or psychological harm or suffering to women, including threats of such acts, coercion or arbitrary deprivation of liberty, whether occurring in public or in private life” (7). This definition allows for a range of perpetrators and a range of forms of violence. The perpetrator may be a husband or partner (intimate partner violence), a family or household member, or someone known or unknown outside the domestic environment (in this paper, we define domestic violence as falling into either or both of the first two categories). **Figure 1** conceptualises the intersecting forms of domestic violence: physical, sexual, emotional or psychological, control, neglect, or economic. These forms of violence overlap and occur in combination, in some settings with forms such as female genital mutilation and modern slavery.

There is consensus that measuring the scope and prevalence of violence against women is important, and that we should try to measure its different sources and forms (8). Doing so may help identify the harm it can cause, advocate on behalf of individual women and against an unacceptable societal burden of coercive control (a perspective that owes much to the work of feminists over the last half century), and compare research findings across time and place and in a shared language (9). We should be aware, however, that an emphasis on definition and metrics is characteristic of a public health approach (10). As feminists have emphasized, violence against women is equally a human rights and criminal justice concern (11), the product of an ecology of micro, meso, and macro factors (12), and a manifestation of structural violence and gendered power imbalances. Too tight a focus on its epidemiology might depoliticize violence against women and shift attention away from these issues (13).

Surveys have been important in international efforts to establish prevalence, the most prominent being the WHO multi-country study on women’s health and domestic violence against women (14) and the International Violence against Women Surveys (15). Choosing and comparing between the available scales and questionnaires is difficult (16, 17). The challenges to measurement are substantial (18), not least in terms of definitions and terminology (19). Early surveys focused on intimate partner physical violence. The Conflict Tactics Scale (20), on which they have often been based, has been extended to include sexual and emotional violence (21), and the definition has been extended to include perpetrators other than intimate partners (22). Whatever has been achieved in terms of definitions and scales, a fundamental challenge remains disclosure: women may not perceive, acknowledge, or communicate that they are surviving violence (23, 24). This is a central issue for clinical and social service provision (25-29), makes crime and hospital records difficult to use in estimating prevalence, and extends to interviews in surveys.

India is a signatory to the Convention on the Elimination of All Forms of Discrimination Against Women (30). Violence against women is addressed by criminal law, particularly domestic violence in Section 498-A of the Indian Penal Code, and by civil law in the form of the Protection of Women from Domestic Violence Act, 2005, which encompasses the varied forms of domestic violence and its perpetrators (available at

For peer review only - http://bmjopen.bmj.com/site/about/guidelines.xhtml

https://indiacode.nic.in/bitstream/123456789/2021/1/200543.pdf). A systematic review of 137 studies suggested that 22% of women in India had survived physical violence in the past year (131 studies), 22% had suffered emotional or verbal psychological violence (60 studies), 7% sexual violence (79 studies), and 30% multiple forms of violence (31). Women in India also commonly experience forms of violence that extend to coercive control and neglect (32), articulated in the idea of gender-based household maltreatment (33), and captured in the recent Indian Family Violence and Control Scale (34).

We work on primary, secondary, and tertiary prevention of violence against women in informal settlements in Mumbai. The prevalence of physical and emotional violence in such settings has been reported as high (35-38). At the beginning of a programme of community mobilisation to prevent violence against women and girls, we conducted a baseline survey. Our objectives in this analysis were to estimate the prevalence of physical, sexual, and emotional violence, control and neglect. We were particularly interested in sources of domestic violence other than intimate partners and in forms of violence other than physical.

Methods

Setting

One in four of the world's urban residents live in informal settlements (slums) (39), characterized by overcrowding, insubstantial housing, insufficient water and sanitation, lack of tenure, and hazardous location (40). In India, the state of Maharashtra accounts for 18% of the national total: more than 100 million residents (41). Just over 40% of Mumbai homes are in informal settlements. The Census of India describes these as areas in which dwellings are unfit for human habitation as a result of dilapidation, overcrowding, and poor building and street design, with associated poor ventilation, light, and sanitation (42).

SNEHA (Society for Nutrition, Education and Health Action) is a non-government organization working to improve health in such settlements. The SNEHA programme on Prevention of Violence against Women and Children began in 2000. Primary prevention is addressed through a combination of community group activities and resulting individual voluntarism. Secondary prevention includes local crisis response and psychological first aid by community organisers and referral to centres which provide counselling, legal, and psychotherapeutic support, with links to the police and medical, shelter, and social service providers. Tertiary prevention is provided primarily through referral to psychiatric and legal services. Having satisfied criteria for counselling, shelter, legal aid, and access to medical care, SNEHA is a service provider under the Protection of Women from Domestic Violence Act, 2005, and is authorised to file Domestic Incident Reports. Under the Protection of Children from Sexual Offences Act, 2012, SNEHA has reporting rights to the police and Child Welfare Committee.

Design

We did a cross-sectional survey of women's experience of violence, collected at the beginning of a cluster randomized controlled trial of community interventions to prevent violence against women and girls (the SNEHA TARA trial) (43).

Sample size

We aimed to complete approximately 100 questionnaires in each of 50 clusters, giving a total sample of around 5000. An estimate of prevalence from a cross-sectional sample of 5000 in a population of 125,000 would have a precision of ~1%. Within this, a comparison of two categories of determinant for 100 respondents in each of 50 clusters would provide 80% power to detect a difference of 6% in prevalence estimates of 10-20%.

Sampling process

Potential clusters were identified in four phases in two areas of the city, and 50 were selected on the basis of (a) vulnerability assessed against a scorecard (44), (b) no known

For peer review only - http://bmjopen.bmj.com/site/about/guidelines.xhtml

plans for rehabilitation or demolition in the next two years, (c) absence of existing nongovernment organization programs addressing violence against women, (d) more than 75% of structures residential and less than 25% rental, and (e) clear separation from each other.

Data collection

Structures and homes within clusters were mapped and 16 women interviewers with graduate education and three months training visited households to enumerate residents and list possible participants. Interviewers began at a random start point and visited every second household to enroll participants. Inclusion criteria were that respondents should be women aged 18-49 years who were usual residents. When there was more than one potential respondent in a household, the investigator applied an algorithm that selected the youngest woman at risk of disability, followed by the youngest married woman, followed by the youngest unmarried woman.

The questionnaire included modules on general health and wellbeing, common mental disorder, household decision-making, household power and control, neglect, experience of economic, emotional, physical, and sexual violence, disclosure and support. Questions were taken from existing Hindi versions where possible. If not, they were translated from English, piloted in two clusters external to the trial, amended, and back-translated.

Interviewers were all women and provided both time and sufficient information for women to consider whether to participate. They were supported by three field supervisors with direct linkage to counselling services, available by phone at any time. The interview team visited the local police station and social services to discuss their activities before starting in each area. Interviewers worked in groups of 7-8 in one cluster at a time, accompanied by a supervisor. A pair worked together in each lane and administered interviews in adjacent households.

To ensure privacy, interviews were arranged by advance appointment and avoided times when partners or children were likely to return from work or school. Women were interviewed at home or in a local community office if they preferred it. The interview began with general questions about demography, household residents, education, socioeconomic position, maternity, and health. If a family member, neighbour, or friend entered, the interviewer went back to asking questions about general health. If the person showed signs of staying, the interview was terminated and completed over up to three repeat visits. As a result of the gatekeeper consent process, community members were aware that interviewers would be visiting people in their area and this limited curiosity and intrusion.

Data management

Interviewers used electronic tablets to enter information in a database in CommCare (www.dimagi.com). To optimize accuracy, the system included field constraints, lookup tables, and automated skip logic. Mobile connectivity allowed immediate contact between interviewers and supervisors. We examined variation in prevalence rates by interviewer and discussed performance in supervisory meetings. We selected for field observation interviewers who showed signs of deviation from the group average and provided feedback where necessary.

Statistical analysis

In developing our questionnaire, we aimed to provide data that would allow comparisons between studies and settings. We included questions from major surveys, international and national, and it should be possible to develop summary measures of violence against women using any of a number of combinations of questions. Our idea was to make the resulting anonymized dataset available so that others might use it, choosing specific questions to facilitate comparison or pooling of data. Here we present composite indicators of lifetime and 12-month prevalence of physical, sexual, and emotional violence.

For peer review only - http://bmjopen.bmj.com/site/about/guidelines.xhtml

Table 1 summarises the questions in three surveys from India (our TARA Trial questionnaire, the National Family Health Survey (NFHS-4) (45), and the Indian Family Violence and Control Scale (34), and two international surveys (the International Violence Against Women Survey (15) and the WHO multi-country study (14)).

Our composite estimate of prevalence of physical violence included nine questions that were comparable across surveys, although there was variation in whether threats of violence were considered incidents of physical violence. The estimate of prevalence of domestic sexual violence included four questions. The comparable questions about sexual violence were similar, although only the TARA survey and the Indian Family Violence Survey included a question on coercion to replicate activities seen in pornographic material. The estimate of prevalence of domestic emotional violence included five questions that were comparable across surveys. We asked a series of questions about domestic coercive control, addressing mobility, education, employment, appearance, and pregnancy. For control and neglect, we present the individual questions because composite indicators are not yet established.

We used the Washington Group Short Set on Functioning questions to identify disability, including difficulty with seeing, hearing, walking, remembering, self-care, and speaking (http://www.washingtongroup-disability.com/washington-group-question-sets/short-set-of-disability-questions/). We took the most permissive of four possible approaches to classification, in which disability is registered if the respondent reports at least some difficulty in at least one of the six domains (46). We classified socioeconomic position with quintiles of scores derived from standardized weights for the first component of a principal components analysis of 22 individual household assets (47, 48).

Prevalence is summarized by frequency and percentage. We plotted prevalence of physical, sexual, and emotional violence in the last year by age group, schooling, socioeconomic asset quintile, and disability, collapsed by grouping variable and rounded using the twoway (connected) command in Stata 15. We calculated odds ratios for associations using logistic regression and Stata survey commands with cluster as the primary sampling unit and phase as stratum. We calculated intracluster correlation coefficients (ICC) for prevalence of physical, sexual, and emotional violence in the last year using the loneway command in Stata 15. The paper was prepared according to the STROBE Statement for reports of observational studies (www.strobe-statement.org).

Ethical considerations

We took permission for the survey from cluster gatekeepers identified by residents (49, 50), and followed WHO guidelines for research on domestic violence against women (51), and on sexual violence (52). Participants gave signed consent after discussing a participant information sheet. We made provision for storage of participant information sheets on women's behalf if they were concerned that the paperwork might be seen by others.

Data collectors explained their right to not provide answers to specific questions, to terminate the interview, and to withdraw temporarily or permanently without penalty. All team members were trained in Good Clinical Practice for research ethics and participant protection (Scientia Clinical Services, 14th February 2019), and interviewers were supported by three field supervisors with direct linkage to counselling services.

Interviewing women about their possible experience of violence raises issues of consent, interviewer behaviour, privacy, and confidentiality. Of particular concern is the duty of care after disclosure. We believe that an interviewee who discloses experience of violence should be offered optimal support, and interviewers were members of a broader team who were able to provide a full suite of crisis and counselling services, including home visits, medical, surgical, and psychiatric referral, and negotiation with families, the police, and legal representatives. When survivors disclosed violence, we followed established intervention protocols which included safety assessment, counselling, liaison with healthcare, police, and legal services, and developing follow-up plans for the survivor and her family. Participants

For peer review only - http://bmjopen.bmj.com/site/about/guidelines.xhtml

were able to speak with counsellors immediately by phone. When a survivor was not ready to disclose violence, the interviewer provided her with information on available services and legal rights and gave her a small card that was easy to hide and listed essential contact numbers and addresses for 24-hour crisis support, medical emergencies, and the police. She took consent for any action from the participant herself.

The trial and associated data collection were approved by the Institutional Ethics Committee of Partners for Urban Knowledge, Action, and Research (PUKAR) (25th December 2017), and the University College London Research Ethics Committee (3546/003, 27th September 2017). The TARA trial within which data collection took place is registered with the Controlled Trials Registry of India (CTRI/2018/02/012047, 21st February 2018) and with ISRCTN84502355 (22nd February 2018: http://www.isrctn.com/ISRCTN84502355).

Patient and public involvement

Community members have been involved in the design of our research since 2000. It arises from the need to provide and improve services for survivors of violence, and a commitment to working with communities to prevent it. Research questions and outcome measures were derived from international efforts, but client priorities were reflected in some specific questions that we asked as a result of client experiences. All questions were discussed with participants in pilot exercises in order to understand their perceptions and ability to respond. Community guardians were involved in recruitment and the practicalities of administering the survey. Respondents took about an hour to answer the questions, and participated only after consideration. The general findings will be disseminated through community groups involved in ongoing interventions in the study areas.

Results

Participants

Between 5th December 2017 and 28th March 2019, 5277 households were approached for the survey. 423 (2%) had no eligible woman resident and in 544 (3%) an eligible woman was unavailable after three visits. Interviewers were unable to achieve privacy in 592 cases (11%) and 155 (3%) potential respondents declined interview. The final survey included 5122 respondents. A median 101 interviews were achieved in each cluster (interquartile range 100-103; range 94-118).

Table 2 summarises characteristics of households, respondents, and their partners. Dwellings were generally of robust or mixed robust and insubstantial construction (93%). Only 17% had their own toilet. Respondents were predominantly of Hindu (59%) or Muslim (37%) faith and 58% described themselves as of general caste. 68% of women had been born outside Mumbai, 96% had been married or partnered, 2% had separated or divorced, and 2% had been widowed. 36% had married before the age of 18 years, 88% of them by arrangement, 14% had had a pregnancy before the age of 18 years, and 36% had three children or more. Women were a mean 32 years old (SD 7.3) and their partners a mean 36 (SD 8.1). 18% of women had had no schooling, compared with 10% of partners, and 36% had completed high school, compared with 48% of partners. Only 24% of women had undertaken paid work in the last year, compared with 98% of men. For these women, the main types of employment were home-based (62%), informal or formal services (17%), and domestic labour (10%). The main types of employment for men were in informal or formal services (49%) or as drivers (18%).

Domestic physical violence

Table 3 presents lifetime and 12-month prevalence of domestic violence. It includes individual questions and composite estimates for physical (9 questions), sexual (4 questions), and emotional violence (5 questions). 25% of women had ever experienced domestic physical violence and 13% had experienced it in the last year. This was usually intimate partner violence (11%), but 2% had experienced physical violence from their marital

For peer review only - http://bmjopen.bmj.com/site/about/guidelines.xhtml

family and 2% from their natal family. Around 4% of women had been kicked, dragged, or beaten in the last year, and around 3% had been threatened or attacked with a blunt or sharp object.

Domestic sexual violence

6% of women had ever experienced domestic sexual violence and 4% had experienced it in the last year, almost all by their intimate partner. Removing the question about coercion to replicate pornography from the composite estimate reduced the estimated prevalence of sexual violence by 0.1% (domestic sexual violence to 5.4% ever (274 reports) and 3.5% in the last year (179), and intimate partner sexual violence to 5.1% ever (262) and 3.5% in the last year (177). Additional questions suggested that 2.5% of partners had insisted on repeated intercourse (126 women in the last year), that 1.1% of wives had been forced to view pornography (56), that 0.8% had been subjected to coercive sex without contraception (39), and that 1.0% of partners were reported as withholding sexual pleasure from their wives (49). The prevalence of domestic physical or sexual violence in the last 12 months was 14% for currently married women, but 29% for women who had separated from their partner.

Domestic emotional violence

19% of women had experienced emotional violence in the last year. The source was roughly evenly split between intimate partner (12%) and marital family members (11%). Emotional violence came from both intimate partner and marital family for 5% of women. 6% of women had been accused of infidelity, 6% had been insulted for being a woman, and 8% of 505 women with disability had been insulted for it.

Summary figures

Limiting the dataset to 4913 ever-married women for comparison with surveys such as the NFHS, the prevalence of domestic physical violence in the last 12 months was 13%, of sexual violence 4%, of physical or sexual violence 14%, and of emotional violence 19%. Recent guidance suggests that studies present the proportion of ever-partnered women and girls aged 15 years and older who have survived physical, sexual, or psychological violence by an intimate partner in the last year (53). For intimate partner violence (a subgroup within domestic violence) our findings were 11% for physical violence, 4% for sexual, 13% for emotional (psychological), 13% for physical or sexual, and 17% for physical, sexual, or emotional violence.

Figure 2 shows prevalence of physical, sexual, and emotional violence for each of 50 clusters. The ranges were substantial: for physical violence 2-27% with interquartile range (IQR) 12-16%, for sexual violence 0-8% (IQR 2-5%), and for emotional violence 7-32% (IQR 15-23%). Intracluster correlation coefficients (ICC) were 0.023 for domestic physical violence in the last year (95% CI 0.011, 0.036), 0.005 for sexual violence (0.000, 0.010), and 0.016 for emotional violence ICC (0.006, 0.026).

Figure 3 summarises prevalence of physical, sexual, and emotional violence by age group, schooling, socioeconomic asset quintile, and disability. Women were less likely to face physical and emotional violence as they aged: the prevalence of physical violence halved between the twenties and the forties. There was no obvious pattern for schooling. All three forms of violence were more common against women in the lowest socioeconomic asset quintile and against women with disability. The odds ratios in **Figure 3** did not change substantially when all four covariates were included in multivariable models, apart from for disability, which was associated with an adjusted odds ratio of 1.68 (95% confidence interval 1.24, 2.29) for physical violence, 2.18 (1.37, 3.47) for sexual violence, and 1.66 (1.31, 2.12) for emotional violence.

Domestic coercive control

For peer review only - http://bmjopen.bmj.com/site/about/guidelines.xhtml

Table 4 summarises forms and sources of coercive control. We took a conservative approach to classifying controlling behaviour. A restrictive behaviour was registered only if it was reported as always occurring, and a permissive behaviour only if it was reported as never occurring. Overall, 1816 women reported being subject to at least one controlling behavior in the list in Table 4 (35%), although we recorded responses to a larger set of questions. Respondents commonly reported that their mobility and socializing were restricted: 26% said that they always required permission to go out, 14% that they were never allowed out in the evening, and 10% that they were always accompanied when they did go out. 48% were never allowed to meet with male friends, 10% were never allowed to meet with female friends, and 5% were never allowed to meet their natal family. At home, 33% of women said that they were afraid of family members. 13% were never free to speak on the phone and 10% were never able to speak freely in the home. These kinds of restrictions were described as coming more often from marital family members than from partners. In terms of sexual and reproductive health, 52 women had been denied access to contraception and 15 had been coerced to use it. 37 (1% of 4577) had been prevented from having a termination of pregnancy, while 15 (0.3%) had been coerced to have one.

Domestic neglect

7% of women reported that their family had neglected their food in the last year, 8% that they had neglected their sleep, 8% that they had neglected their health, and 12% of 4375 with children that they had neglected their children's health. Collectively, 10% of women reported at least one of these four dimensions of neglect.

Disclosure of violence

Of 1153 women who had survived physical, sexual, or emotional domestic violence in the last year, 47% had disclosed to someone. The main confidants were family and friends (40%) and teachers, faith leaders, or local leaders (4%). Even though 18% had sought medical help, only 8% had disclosed to a healthcare provider, social worker, or counsellor. Twenty women approached our services for support within two months of the survey.

Discussion

Key findings

Our cross-sectional survey of 5122 women aged 15-49 years in informal settlements in Mumbai documented high rates of domestic violence. In the preceding year, 13% of women had suffered physical violence, 4% sexual violence, and 19% emotional violence. Physical and sexual violence was perpetrated mainly by intimate partners, but emotional violence was perpetrated equally by intimate partners and marital family members. Controlling behaviours were ascribed to both intimate partners and marital family members, with 35% of women reporting at least one of 12 restrictions.

Limitations

Our findings have three general limitations. First, although the prevalence of domestic violence was high, we should assume some underreporting given the constellation of forces inhibiting disclosure. Second, we preferentially recruited younger, married, and disabled women within households, which would tend to increase reported prevalence. Third, our study was cross-sectional and subject to the usual caveats around causal inference and the accuracy of time recall.

Comparison with other studies

We updated the review of quantitative studies by Kalokhe and colleagues (31), identifying publications from 1st January 2015 to 1st November 2019 using similar search terms, and found eight more recent published studies. The reported prevalence of domestic violence was higher in rural (54-56) than in urban settings. The estimated lifetime prevalence of physical violence ranged from 18% (57) to 27% (58, 59), of sexual violence from 10% (57) to

For peer review only - http://bmjopen.bmj.com/site/about/guidelines.xhtml

26% (59), and of emotional violence from 20% (57) to 43% (this high estimate included questions on economic violence)(59). A cross-sectional survey similar to ours, of 1137 mothers aged 18-39 years in an informal settlement in Mumbai, found a lifetime prevalence of physical intimate partner violence of 17%, of emotional violence of 12%, and of sexual violence of 5% (60).

Our findings also compare with the NFHS-4 for Maharashtra state, in which estimates of prevalence of intimate partner violence were based on interviews with 2472 ever-married women aged 15-49 years (61). Both surveys estimated lifetime physical violence at 21%. The NFHS-4 estimate for the last 12 months was 15% (our study 11%). We found higher prevalence of sexual (5% ever compared with 2% in the NFHS-4; 4% in the last 12 months compared with 2%) and emotional violence (17% ever compared with 10% in the NFHS-4; 12% in the last 12 months compared with 7%). Our estimates of prevalence of all three types of violence were higher than those of the urban sample in the Maharashtra NFHS-4, which interviewed 1220 women (lifetime intimate partner physical violence 21% in our study, compared with 16% in the NFHS-4, sexual violence 5% compared with 2%, and emotional violence 17% compared with 9%). There are three possible explanations for our higher estimates. First, they might be based on different sets of questions: we used similar questions and composites of them and do not think this was an issue. Second, they might reflect true population differences: our participants were all residents of informal settlements and might be more likely to face violence than the urban average. Third, women might have been more likely to disclose violence to our team of interviewers, who were backed up by a strong programme of support for survivors. It is also possible that women answer questions differently when they are responding to a survey on violence than when the questions on violence are included in a broad survey like the NFHS-4. In a consideration of methodological issues in research on intimate partner violence, Saltzman suggests that questionnaire context and cues given to the participant can influence response (10).

Implications

We have a clear picture of the high burden of domestic violence against women, and of the range of findings across urban and rural settings in India. The odds of physical violence are greater for younger married women, with poorer socioeconomic position, and where underage marriage is common (62). We found that 47% of women who had suffered violence had sought help from somebody. This is considerably more than the NFHS-4, which found that 14% of urban women who had experienced physical or sexual violence had sought help from any source, usually natal family, marital family, friend or neighbor. Although disclosure to a healthcare provider, social worker, or counsellor was uncommon (8%), it was also higher than the NFHS-4 urban figure of 3%. Perhaps this provides some hope. Awareness of domestic violence is certainly increasing and it may be that women are becoming more confident that what they are going through is violence and that it is not acceptable.

Our particular concerns are perpetration of violence by the marital family and the burden of emotional violence and coercive control. Although only 11% of studies in the recent review considered non-partner violence (31), domestic violence by people other than intimate partners has long been a concern in the Indian context (63). Women entering the marital household are traditionally subordinate and the spectrum of violence extends from subtle neglect to dowry death. Toleration of their subservience in exchange for security and later authority over their own daughters-in-law has been described as the patriarchal bargain (64), and the burden of violence against women by mothers-in-law (65) and other family members has been termed gender-based household maltreatment. It informed the design of our own questionnaire (66) and includes control over reproduction, limiting contact with friends and family, and access to food (32, 34). These kinds of less visible emotional violence and control have been less well studied, but they need to be: marital family members were the source of emotional violence in about half of reports in our study and of 35% of domestic violence in rural Tamil Nadu (54).

For peer review only

Recent work on conceptualising and measuring psychological violence (here termed emotional violence) has suggested that it is distinct from controlling behaviour (67). More than one-third of women in our study reported experiencing one of 12 controlling behaviors and other studies have reported high levels of control by intimate partners: 72% in rural Tamil Nadu (55), and 60% in rural Rajasthan (56), 43% in urban Delhi (57), and 12% in Karnataka (68). In a survey of married women in Uttar Pradesh, 12% had experienced reproductive control from husbands or in-laws, with a roughly equal split between them (69).

Although emotional violence and coercive control are less visible than physical violence, the gravity of their effects on women's mental health is being increasingly appreciated (70). Follingstad identified six types of emotional abuse that survivors of violence in the United States had experienced: verbal attacks (ridicule, name-calling, humiliation in public), isolation (social or financial), jealousy and possessiveness (including accusations of infidelity), threats of harm, threats of divorce or abandonment, and destruction of personal property. All had strong negative effects on women and 73% said that emotional violence had greater impact than physical violence (71). This finding is supported by both qualitative (71, 72) and quantitative research in high-income countries (73). In an analysis of the US National Violence Against Women Survey, psychological violence was more strongly associated with depressive symptoms than physical violence, and abuse of power and control were more strongly associated with depressive symptoms than verbal abuse (74).

We are seeing similar evidence from India. In rural Rajasthan, Richardson and colleagues "… found evidence that psychological abuse and controlling behavior were more damaging to mental health than physical abuse" (56). A cross-sectional survey of 9938 mothers aged 15-49 years in seven urban and rural locations, of whom 3155 lived in urban informal settlements, suggested that severe harassment by in-laws was associated with poor mental health (75). An analysis of follow-up data from 6303 rural married women aged 15-49 years in Bihar, Jharkhand, Maharashtra, and Tamil Nadu showed a strong association between 'verbal' violence and mental health and a much weaker association with physical violence (76).

Generalisability

While domestic violence is a global experience, there are differences in cultural experiences. A feature of the discussion in high-income countries from the 1970s onward was the disappointment of professional providers of support when a survivor of intimate partner violence did not leave the perpetrator (23, 24, 77). The emphasis in India – on the part of survivors and professionals – has been on interventions that allow families to remain together. Tolerating domestic violence has been a normalized element of women's reproductive labour (78, 79), and the ability to bear it and still fulfil the expectations of a wife and mother has been seen as praiseworthy (80, 81) and characteristic of the 'real' or 'good' woman (82). Even when domestic violence spills out into community knowledge, women who break with this tradition risk social ostracism (83), and their perceptions of their options are limited (27, 65)

Conclusion

Domestic violence against women continues to be common in urban informal settlements in Mumbai and we see no reason to doubt the external validity of our findings for other locations. Physical and sexual violence is perpetrated mainly by intimate partners, but emotional violence against women is attributed equally to partners and marital family. More than one-third of women describe at least one of a limited set of controlling behaviours, perpetrated more by intimate partners, but often by marital family members.

We have two recommendations for research and action. First, social workers and healthcare providers should be aware of the importance of emotional violence and coercive control. Both are common and cause substantial suffering, particularly to women's mental health. These forms of violence need to be considered in interactions with women because of their

For peer review only - http://bmjopen.bmj.com/site/about/guidelines.xhtml

subtlety and their intersection with poverty in urban informal settlements. Researchers should make sure that they are included in studies of domestic violence.

Second, violence is often perpetrated by family members other than intimate partners. Again, this is particularly true of emotional violence and coercive control. In interacting with clients, social workers and healthcare providers need to be aware that family members accompanying them might be involved in abuse, and studies should assume the possibility of other perpetrators.

Acknowledgments

Our greatest thanks go to the women and community guardians who agreed to contribute to the study. We thank the team of investigators who collected data in challenging conditions, Miheeka Vast and Manju Singh for supervising the field investigation team, Unnati Machchhar and Shilpa Adelkar for their supervision of the intervention programme, Gauri Savkur for contributing to investigator training, Bhaskar Kakad for investigator support, Archana Bagra and Vibhavari Bali for financial and human resources management, and Vanessa D'Souza and Shanti Pantvaidya for leadership at SNEHA.

List of figures and tables

Figure 1. Forms of domestic violence against women

Figure 2. Prevalence of physical, sexual, and emotional violence against 5122 women aged 18-49 years in 50 informal settlement clusters in Mumbai

Figure 3. Prevalence of physical, sexual, and emotional violence against 5122 women aged 18-49 years, by age group, schooling, socioeconomic asset quintile, and disability

Table 1. Comparison of questions in five surveys describing physical, sexual, and emotional violence

Table 2. Characteristics of households, respondents, and their partners

Table 3. Lifetime and 12-month prevalence of domestic physical, sexual, and emotional violence against 5122 women aged 18-49 years, by perpetrator: intimate partner, marital family, or natal family member

Table 4. Lifetime prevalence of domestic control of 5122 women aged 18-49 years, by perpetrator: intimate partner, marital family, or natal family member

Contributorship

ND and DO conceived the study and acquired funding. ND, AG, SK, LG, and DO developed the methodology, ND, SK, and AG oversaw investigation, and SK and DO curated the data. DO did the first analysis and visualization and wrote the first draft. ND managed the project with support from SK. All authors critically reviewed drafts of the manuscript and read and commented on the final version.

Competing interests

The authors declare no competing interest.

Funding

Wellcome Trust 206417. The funder had no role in study design, data collection and analysis, decision to publish, or preparation of the manuscript.

Data sharing

Data are available in Open Science Framework: *Osrin, D. (2020, June 30). Prevalence survey. Retrieved from osf.io/khdcm*

References

For peer review only

1. Ellsberg M, Jansen H, Heise L, Watts C, Garcia-Moreno C, on behalf of the WHO Multi-country Study on Women's Health and Domestic Violence against Women Study Team. Intimate partner violence and women's physical and mental health in the WHO multi-country study on women's health and domestic violence: an observational study. Lancet. 2008;371:1165-72.

2. Stöckl H, Devries K, Rotstein A, Abrahams N, Campbell J, Watts C, et al. The global prevalence of intimate partner homicide: a systematic review. Lancet. 2013;382:859-65.

3. Satyanarayana VA, Chandra PS, Vaddiparti K. Mental health consequences of violence against women and girls. Curr Opin Psychiatry. 2015;28:350-6.

4. WHO. Global and regional estimates of violence against women: prevalence and health effects of intimate partner violence and non-partner sexual violence. Geneva: World Health Organization; 2013. Report No.: 9789241564625.

5. Garcia-Moreno C, Zimmerman C, Morris-Gehring A, Heise L, Amin A, Abrahams N, et al. Addressing violence against women: a call to action. Lancet. 2015;385:1685-95.

6. Solotaroff JL, Pande RP. Violence against women and girls: lessons from South Asia. Washington DC; 2014.

7. UN General Assembly. Declaration on elimination of violence against women. GA res. 48/104, 48 UN GAOR. Supp. No. 49 at 217, UN Doc. A/48/49. www1.umn.edu/humanrts/instree/e4devw.htm; 1993.

8. UNDAW. Indicators to measure violence against women. Report of the Expert Group Meeting. Geneva: United Nations Division for the Advancement of Women, United Nations Economic Commission for Europe, United Nations Statistical Division; 2007.

9. Gelles RJ. Estimating the incidence and prevalence of violence against women. Violence Against Women. 2000;6:784-804.

10. Saltzman LE. Definitional and methodological issues related to transnational research on intimate partner violence. Violence Against Women. 2004;10:812-30.

11. Kilpatrick DG. What is violence against women? Defining and measuring the problem. J Interpers Violence. 2004;19:1209-34.

12. Heise L. Violence against women: an integrated ecological framework. Violence Against Women. 1998;4:262-90.

13. Piedalue A. Understanding violence in place: travelling knowledge paradigms and measuring domestic violence in India. Ind J Gender Stud. 2015;22.

14. Garcia-Moreno C, Jansen HA, Ellsberg M, Heise L, Watts C. WHO multi-country study on women’s health and domestic violence against women: initial results on prevalence, health outcomes and women’s responses. Geneva: World Health Organization; 2005.

15. Johnson H, Ollus N, Nevala S. Violence against women: an international perspective. New York NY: European Institute for Crime Prevention and Control (HEUNI); 2008.

16. Waltermaurer E. Measuring intimate partner violence (IPV): you may only get what you ask for. J Interpers Violence. 2005;20:501-6.

17. Thompson MP, Basile KC, Hertz MF, Sitterle D. Measuring intimate partner violence victimization and perpetration: a compendium of assessment tools. Atlanta GA: Centers for Disease Control and Prevention, National Center for Injury Prevention and Control; 2006.

18. Ruiz-Pérez I, Plazaola-Castaño J, Vives-Cases C. Methodological issues in the study of violence against women. J Epidemiol Commun Health. 2007;61:ii26-ii31.

For peer review only - http://bmjopen.bmj.com/site/about/guidelines.xhtml

19. Bagwell-Gray ME, Messing JT, Baldwin-White A. Intimate partner sexual violence: a review of terms, definitions, and prevalence. Trauma Violence Abuse. 2015;16:316-35.

20. Straus M. Measuring intrafamily conflict and violence: the Conflict Tactics (CT) Scales. J Marriage Family. 1979;41:75-88.

21. Straus M, Hamby S, Boney-McCoy S, Sugarman D. The Revised Conflict Tactics Scales (CTS2) development and preliminary psychometric data. J Family Issues. 1996;17:283-316.

22. Tjaden P. Defining and measuring violence against women: background, issues, and recommendations. Geneva: UN Division for the Advancement of Women, Economic Commission for Europe, World Health Organization; 2005.

23. Ferraro KJ, Johnson JM. How women experience battering: the process of victimization. Social Problems. 1983;30:325-39.

24. Mills T. The assault on the self: stages in coping with battering husbands. Qualitative Sociology. 1985;8:103-23.

25. Decker MR, Nair S, Saggurti N, Sabri B, Jethva M, Raj A, et al. Violence-related coping, help-seeking and health care-based intervention preferences among perinatal women in Mumbai, India. J Interpers Violence. 2013;28:1924-47.

26. Jejeebhoy S, Santhya KG, Acharya R. Violence against women in South Asia: the need for the active engagement of the health sector. Global Public Health. 2014;9:678-90.

27. Ragavan M, Iyengar K, Wurtz R. Perceptions of options available for victims of physical intimate partner violence in northern India. Violence Against Women. 2015;21:652-75.

28. Leonardsson M, San Sebastion M. Prevalence and predictors of help-seeking for women exposed to spousal violence in India - a cross-sectional study. BMC Women's Health. 2017;17:99.

29. Vranda MN, Kumar CN, Muralidhar D, Janardhana N, Sivakumar PT. Barriers to disclosure of intimate partner violence among female patients availing services at tertiary care psychiatric hospitals: a qualitative study. J Neurosci Rural Pract. 2018;9:326-30.

30. United Nations. Convention on the elimination of all forms of discrimination against women. http://www.un.org/womenwatch/daw/cedaw/. New York: United Nations; 1979.

31. Kalokhe A, del Rio C, Dunkle K, Stephenson R, Metheny N, Paranjape A, et al. Domestic violence against women in India: A systematic review of a decade of quantitative studies. Global Public Health. 2016:1-16.

32. Kalokhe AS, Potdar RR, Stephenson R, Dunkle KL, Paranjape A, del rio C, et al. How well does the World Health Organization definition of domestic violence work for India? PLoS ONE. 2015;10:e0120909.

33. Silverman JG, Donta B, Decker MR, Boyce SC, Ritter J, Naik DD, et al. Family violence and maltreatment of women during the perinatal period: associations with infant morbidity in Indian slum communities. Matern Child Health J. 2016;20:149-57.

34. Kalokhe AS, Stephenson R, Kelley ME, Dunkle KL, Paranjape A, Solas V, et al. The Development and Validation of the Indian Family Violence and Control Scale. PLoS One. 2016;11(1):e0148120.

35. Ruikar MM, Pratinidhi AK. Physical wife abuse in an urban slum of Pune, Maharashtra. Ind J Public Health. 2008;52:215-7.

36. Sinha A, Mallik S, Sanyal D, Dasgupta S, Pal D, Mukherjee A. Domestic violence among ever married women of reproductive age group in a slum area of Kolkata. Indian J Public Health. 2012;56(1):31-6.

For peer review only - http://bmjopen.bmj.com/site/about/guidelines.xhtml

37. Shrivastava PS, Shrivastava SR. A study of spousal domestic violence in an urban slum of Mumbai. Int J Prev Med. 2013;4:27-32.

38. Sabri B, Campbell JC. Intimate partner violence against women in slums in India. Ind J Med Res. 2015;141:757-9.

39. UNDESA. World social report 2020: inequality in a rapidly changing world. New York NY: United Nations Department of Economic and Social Affairs; 2020. Contract No.: ST/ESA/372.

40. United Nations Human Settlements Programme (UN-Habitat). The challenge of slums: global report on human settlements 2003. London and Sterling VA: Earthscan Publications Ltd; 2003.

41. Central Bureau of Health Intelligence. National health profile 2019. New Delhi: Directorate General of Health Services, Ministry of Health and Family Welfare, Government of India; 2019.

42. Chandramouli C. Housing stock, amenities and assets in slums - Census 2011. Available from www.censusindia.gov.in. New Delhi: Office of the Registrar General and Census Commissioner,; 2011.

43. Daruwalla N, Machchhar U, Pantvaidya S, D'Souza V, Gram L, Copas A, et al. Community interventions to prevent violence against women and girls in informal settlements in Mumbai: the SNEHA-TARA pragmatic cluster randomised controlled trial. Trials. 2019;20(1):743.

44. Osrin D, Das S, Bapat U, Alcock GA, Joshi W, More NS. A rapid assessment scorecard to identify informal settlements at higher maternal and child health risk in Mumbai. J Urban Health. 2011;88(5):919-32.

45. IIPS. National Family Health Survey 2015-16 (NFHS-4): interviewer's manual. Mumbai: International Institute for Population Sciences; 2014.

46. Washington Group on Disability Statistics (WG). Understanding and interpreting disability as measured using the WG Short Set of questions. Hyattsville MD; 2009.

47. Filmer D, Pritchett L. Estimating wealth effects without expenditure data - or tears: an application to educational enrollments in states of India. Demography. 2001;38:115-32.

48. Vyas S, Kumaranayake L. Constructing socio-economic status indices: how to use principal components analysis. Health Policy Plan. 2006;21:459-68.

49. Taljaard M, Weijer C, Grimshaw JM, Eccles MP. The Ottawa Statement on the ethical design and conduct of cluster randomised trials: precis for researchers and research ethics committees. BMJ. 2013;346:f2838.

50. Lignou S, Das S, Mistry J, Alcock G, More NS, Osrin D, et al. Reconstructing communities in cluster trials? Trials. 2016;17:166.

51. WHO. Putting women first: ethical and safety recommendations for research on domestic violence against women. WHO/FCH/GWH/01.1. Geneva: Department of Gender and Women's Health, Family and Community Health, World Health Organization; 2001.

52. Jewkes R, Dartnall E, Sikweyiya Y. Ethical and safety recommendations for research on perpetration of sexual violence. Pretoria: Medical Research Council, Sexual Violence Research Initiative; 2012.

53. WHO. Consensus on priority indicators on violence against women Geneva2020 [Available from: https://www.who.int/reproductivehealth/topics/violence/vaw-indicators/en/.

54. George J, Nair D, Premkumar NR, Saravanan N, Chinnakali P, Roy G. The prevalence of domestic violence and its associated factors among married women in a rural area of Puducherry, South India. J Family Med Prim Care. 2016;5(3):672-6.

For peer review only

55. Ram A, Victor CP, Christy H, Hembrom S, Cherian AG, Mohan VR. Domestic violence and its determinants among 15-49-year-old women in a rural block in South India. Ind J Commun Med. 2019;44:362-7.

56. Richardson R, Nandi A, Jaswal S, Harper S. The effect of intimate partner violence on women's mental distress: a prospective cohort study of 3010 rural Indian women. Soc Psychiatry Psychiatr Epidemiol. 2019.

57. Mukherjee R, Joshi RK. Controlling Behavior and Intimate Partner Violence: A Cross-Sectional Study in an Urban Area of Delhi, India. J Interpers Violence. 2019:886260519876720.

58. Nadda A, Malik JS, Rohilla R, Chahal S, Chayal V, Arora V. Study of domestic violence amoung currently married females of Haryana, India. Ind J Psychol Med. 2018;40:534-9.

59. Sharma KK, Vatsa M, Kalaivani M, Bhardwaj D. Mental health effects of domestic violence against women in Delhi: A community-based study. J Family Med Prim Care. 2019;8(7):2522-7.

60. Begum S, Donta B, Nair S, Prakasam CP. Socio-demographic factors associated with domestic violence in urban slums, Mumbai, Maharashtra, India Indian J Med Res. 2015;141:783-8.

61. International Institute for Population Sciences (IIPS), ICF. National Family Health Survey (NFHS-4), India, 2015-16: Maharashtra. Mumbai: IIPS; 2018.

62. Ahmad J, Khan NH, Mozumdar A. Spousal Violence Against Women in India: A Social-Ecological Analysis Using Data From the National Family Health Survey 2015 to 2016. J Interpers Violence. 2019(Oct 23):886260519881530.

63. Kumar V, Kanth S. Bride burning. Lancet. 2004;364 Suppl 1:s18-9.

64. Kandiyoti D. Bargaining with patriarchy. Gender and Society. 1988;2:274-90.

65. Ragavan M, Iyengar K. Violence perpetrated by mothers-in-law in Northern India: perceived frequency, acceptability, and options for survivors. J Interpers Violence. 2017(May 1):886260517708759.

66. Ritter J, Dasgupta A, Raj A, Decker MR, Nair S, Trivedi A, et al. Measuring gender-based household maltreatment in urban slums of Mumbai, India. J Interpers Violence. 2019;1-23(Jul 31):886260519864370.

67. Heise L, Pallitto C, García-Moreno C, Clark CJ. Measuring psychological abuse by intimate partners: constructing a cross-cultural indicator for the Sustainable Development Goals. SSM Popul Health. 2019;9:100377.

68. Kundapur R, Shetty SM, Kempaller VJ, Kumar A, Anurupa M. Violence against Educated Women by Intimate Partners in Urban Karnataka, India. Indian J Community Med. 2017;42(3):147-50.

69. Silverman JG, Boyce SC, Dehingia N, Rao N, Chandurkar D, Nanda P, et al. Reproductive coercion in Uttar Pradesh, India: Prevalence and associations with partner violence and reproductive health. SSM Popul Health. 2019;9:100484.

70. Jewkes R. Emotional abuse: a neglected dimension of partner violence. Lancet. 2010;376:851-2.

71. Follingstad DR, Rutledge LL, Berg BJ, Hause ES, Polek DS. The role of emotional abuse in physically abusive relationships. J Family Violence. 1990;5:107-20.

72. Vitanza S, Vogel LCM, Marshall LL. Distress and symptoms of posttraumatic stress disorder in abused women. Violence Victims. 1995;10:23-34.

For peer review only

73. Mechanic MB, Weaver TL, Resick PA. Mental health consequences of intimate partner abuse: a multidimensional assessment of four different forms of abuse. Violence Against Women. 2008;14:634-54.

74. Coker AL, Davis KE, Arias I, Desai S, Sanderson M, Brandt HM, et al. Physical and mental health effects of intimate partner violence for men and women. Am J Prev Med. 2002;23:260-8.

75. Kumar S, Jeyaseelan L, Suresh S, Ahuja RC. Domestic violence and its mental health correlates in Indian women. Br J Psychiatry. 2005;187:62-7.

76. Stephenson R, Winter A, Hindin M. Frequency of intimate partner violence and rural women's mental health in four Indian states. Violence Against Women. 2013;19:1133.

77. Gelles RJ. Abused wives: why do they stay. J Marriage Family. 1976;38:659-68.

78. Anitha S. Understanding economic abuse through an intersectional lens: financial abuse, control, and exploitation of women's productive and reproductive labor. Violence Against Women. 2019;25:1854-77.

79. Jejeebhoy SJ, Cook RJ. State accountability for wife-beating: the Indian challenge. Lancet. 1997;349(Suppl 1):s10-2.

80. Narula DK, Narula A. Patriarchal societies and domestic violence: need for empowering women. In: Charak P, Mohan C, editors. Women and development: self, society and empowerment. New Delhi: Primus Books; 2012. p. 169-83.

81. Ahmed-Ghosh H. Chattels of society: domestic violence in India. Violence Against Women. 2004;10:94-118.

82. Jejeebhoy SJ, Santhya KG, Sabarwal S. Gender-based violence: a qualitative exploration of norms, experiences and positive deviance. New Delhi: Population Council; 2013.

83. Ghosh S. Watching, blaming, silencing, intervening: exploring the role of the community in preventing domestic violence in India. Practicing Anthropology. 2011;33:22-6.

For peer review only

Table 1. Comparison of questions in five surveys describing physical, sexual, and emotional violence

	SNEHA TARA	NFHS-4 (45)	Indian Family Violence and Control Scale (34)	WHO multi-country study (14)	International Violence Against Women Survey (15)
**Physical**					
Pushed, shoved, shaken, hurt	√	√	√	√	√
Twisted arm, banged head, pulled hair	√	√	√		√
Slapped, pinched, bitten	√	√	√	√	√
Hit, punched	√	√	√	√	
Kicked, dragged, beaten	√	√	√	√	
Things thrown at, burned	√		√	√	
Attacked or threatened with sharp object	√	√	√	√	√
Attacked or threatened with blunt object	√	√	√	√	√
Suffocated, choked, hung, poisoned	√	√	√	√	√
**Sexual**					
Forced intercourse	√	√	√	√	√
Forced other degrading act	√	√	√	√	
Threatened other act	√	√			
Forced to replicate pornography	√		√		
Forced sex during menses	√		√		
Forced sex with someone else	√		√		
Forced sex without contraception	√		√		
Threatened sex with another person	√		√		
Forced video of sex	√		√		
Taken advantage of when drugged or drunk	√		√		
Forced to watch pornography	√				
Insisted on repeated intercourse	√				
Forced to entertain others sexually	√				
Forced drugs or alcohol for sex	√				
Partner withheld sexual pleasure	√				
**Emotional**					
Insulted, made to feel bad about herself	√	√	√	√	
Belittled, humiliated in front of others	√	√	√	√	
Ignored, treated indifferently	√		√	√	
Scared or intimidated on purpose	√		√	√	
Threated to hurt her, someone close, or take child	√	√		√	√
Insulted for not having a baby	√		√		
Insulted for not having a son	√		√		
Afraid of family members	√				
Accused of infidelity	√	√		√	
Insulted for being a woman	√				

For peer review only

Table 2. Characteristics of households, respondents, and their partners

Household	n	(%)
**Fabric**		
Kachha (insubstantial)	353	(7)
Mixed	2149	(42)
Pukka (robust)	2620	(51)
**Toilet**		
Private	875	(17)
Public or charity	4245	(83)
Open defecation	2	(0)
**Religion**		
Hindu	3002	(59)
Muslim	1902	(37)
Buddhist	158	(3)
Other	60	(1)
All	5122	(100)

Individual	Woman respondent		Husband or partner	
	n	(%)	n	(%)
**Age group**				
18-19 y	115	(2)	14	(0)
20-29 y	2038	(40)	920	(19)
30-39 y	2056	(40)	2108	(44)
40-49 y	913	(18)	1372	(29)
50+ y			391	(8)
**Education**				
None	947	(18)	474	(10)
Primary 1-5 y	859	(17)	590	(12)
Middle 6-8 y	1122	(22)	981	(20)
High 9-10 y	1158	(23)	1543	(32)
Senior 11-12 y	572	(11)	698	(15)
Undergraduate	363	(7)	371	(8)
Postgraduate	100	(2)	123	(3)
Other	1	(0)	25	(1)
**Paid work in last 12 m**				
Yes	1252	(24)	4697	(98)
No	3870	(76)	108	(2)
All	5122	(100)	4805	(100)

For peer review only - http://bmjopen.bmj.com/site/about/guidelines.xhtml

Table 3. Lifetime and 12-month prevalence of domestic physical, sexual, and emotional violence against 5122 women aged 18-49 years, by perpetrator: intimate partner, marital family, or natal family member

	Lifetime		Lifetime by perpetrator						Last 12 m		Last 12 m by perpetrator					
	Domestic		Intimate partner		Marital		Natal		Domestic		Intimate partner		Marital		Natal	
	n	(%)	n	(%)	n	(%)	n	(%)	n	(%)	n	(%)	n	(%)	n	(%)
**Physical violence**																
Pushed, shoved, shaken, hurt	595	(11.6)	458	(8.9)	186	(3.6)	33	(0.6)	292	(5.7)	247	(4.8)	70	(1.4)	17	(0.3)
Twisted arm, banged head, pulled hair	464	(9.1)	371	(7.2)	121	(2.4)	23	(0.4)	224	(4.4)	194	(3.8)	43	(0.8)	10	(0.2)
Slapped, pinched, bitten	1154	(22.5)	986	(19.3)	134	(2.6)	139	(2.7)	565	(11.0)	504	(9.8)	52	(1.0)	71	(1.4)
Hit, punched	421	(8.2)	351	(6.9)	90	(1.8)	28	(0.5)	206	(4.0)	182	(3.6)	30	(0.6)	10	(0.2)
Kicked, dragged, beaten	398	(7.8)	326	(6.4)	88	(1.7)	28	(0.5)	199	(3.9)	175	(3.4)	32	(0.6)	10	(0.2)
Things thrown at, burned	51	(1.0)	41	(0.8)	14	(0.3)	0	(0.0)	15	(0.3)	13	(0.3)	3	(0.1)	0	(0.0
Attacked or threatened with sharp object	96	(1.9)	74	(1.4)	22	(0.4)	3	(0.1)	51	(1.0)	44	(0.9)	6	(0.1)	2	(0.0)
Attacked or threatened with blunt object	218	(4.3)	165	(3.2)	51	(1.0)	22	(0.4)	103	(2.0)	90	(1.8)	16	(0.3)	6	(0.1)
Suffocated, choked, hung, poisoned	114	(2.2)	96	(1.9)	20	(0.4)	5	(0.1)	52	(1.0)	48	(0.9)	8	(0.2)	1	(0.0)
**Sexual violence**																
Forced intercourse	231	(4.5)	219	(4.3)	6	(0.1)	4	(0.1)	138	(2.7)	136	(2.7)	1	(0.0)	0	(0.0)
Forced other degrading act	128	(2.5)	126	(2.5)	1	(0.0)	1	(0.0)	95	(1.9)	95	(1.9)	1	(0.0)	0	(0.0)
Threatened other act	74	(1.4)	72	(1.4)	0	(0.0)	1	(0.0)	43	(0.8)	43	(0.8)	0	(0.0)	0	(0.0)
Forced to replicate pornography	75	(1.5)	75	(1.5)	0	(0.0)	0	(0.0)	45	(0.9)	45	(0.9)	0	(0.0)	0	(0.0)
**Emotional violence**																
Insulted, made to feel bad about herself	1296	(25.3)	536	(10.5)	877	(17.1)	99	(1.9)	690	(13.5)	347	(6.8)	419	(8.2)	64	(1.2)
Ignored, treated indifferently	773	(15.1)	339	(6.6)	541	(10.6)	50	(1.0)	449	(8.8)	230	(4.5)	290	(5.7)	34	(0.7)
Belittled, humiliated in front of others	682	(13.3)	313	(6.1)	445	(8.7)	52	(1.0)	417	(8.1)	223	(4.4)	242	(4.7)	35	(0.7)
Scared or intimidated on purpose	841	(16.4)	592	(11.6)	330	(6.4)	67	(1.3)	570	(11.1)	432	(8.4)	191	(3.7)	52	(1.0)
Threated to hurt her, someone close, or take child away	213	(4.2)	147	(2.9)	100	(2.0)	6	(0.1)	130	(2.5)	100	(2.0)	49	(1.0)	4	(0.1)
**Any physical violence**	1293	(25.2)	1084	(21.2)	261	(5.1)	170	(3.3)	644	(12.6)	562	(11.0)	103	(2.0)	84	(1.6)
**Any sexual violence**	289	(5.6)	277	(5.4)	7	(0.1)	4	(0.1)	188	(3.7)	186	(3.6)	2	(0.0)	0	(0.0)
**Any emotional violence**	1599	(31.2)	876	(17.1)	1045	(20.4)	138	(2.7)	963	(18.8)	607	(11.9)	538	(10.5)	90	(1.8)
**All**	**5122**	**(100.0)**	**5122**	**(100.0)**	**5122**	**(100.0)**	**5122**	**(100.0)**	**5122**	**(100.0)**	**5122**	**(100.0)**	**5122**	**(100.0)**	**5122**	**(100.0)**

For peer review only

For peer review only - http://bmjopen.bmj.com/site/about/guidelines.xhtml

Table 4. Lifetime prevalence of domestic control of 5122 women aged 18-49 years, by perpetrator: intimate partner, marital family, or natal family member

Control	Domestic		Intimate partner		Marital		Natal	
	n	(%)	n	(%)	n	(%)	n	(%)
**Mobility**								
Movement always monitored	419	(8.2)	278	(5.4)	140	(2.7)	44	(0.9)
Always prevented from attending meetings	227	(4.4)	129	(2.5)	118	(2.3)	5	(0.1)
**Education**								
Prevented from schooling	152	(3.0)	53	(1.0)	47	(0.9)	62	(1.2)
**Employment**								
Prevented from seeking employment	853	(16.7)	728	(14.2)	162	(3.2)	31	(0.6)
Coerced to seek employment	78	(1.5)	42	(0.8)	35	(0.7)	7	(0.1)
**Home dynamics**								
Excluded from family matters	366	(7.1)	108	(2.1)	300	(5.9)	14	(0.3)
Dress or hairstyle always controlled	267	(5.2)	119	(2.3)	145	(2.8)	25	(0.5)
Limited access to house	173	(3.4)	56	(1.1)	137	(2.7)	7	(0.1)
Forced out of house	165	(3.2)	52	(1.0)	121	(2.4)	5	(0.1)
Locked in house	39	(0.8)	18	(0.4)	22	(0.4)	1	(0.0)
**Health**								
Made to do excessive work	239	(4.7)	39	(0.8)	209	(4.1)	11	(0.2)
Always requires permission for healthcare	753	(14.7)	513	(10.0)	286	(5.6)	48	(0.9)
**Any of the above**	**1816**	**(35.4)**	**1303**	**(25.4)**	**830**	**(16.2)**	**184**	**(3.6)**
**All**	5122	(100.0)	5122	(100.0)	5122	(100.0)	5122	(100.0)

For peer review only

For peer review only - http://bmjopen.bmj.com/site/about/guidelines.xhtml

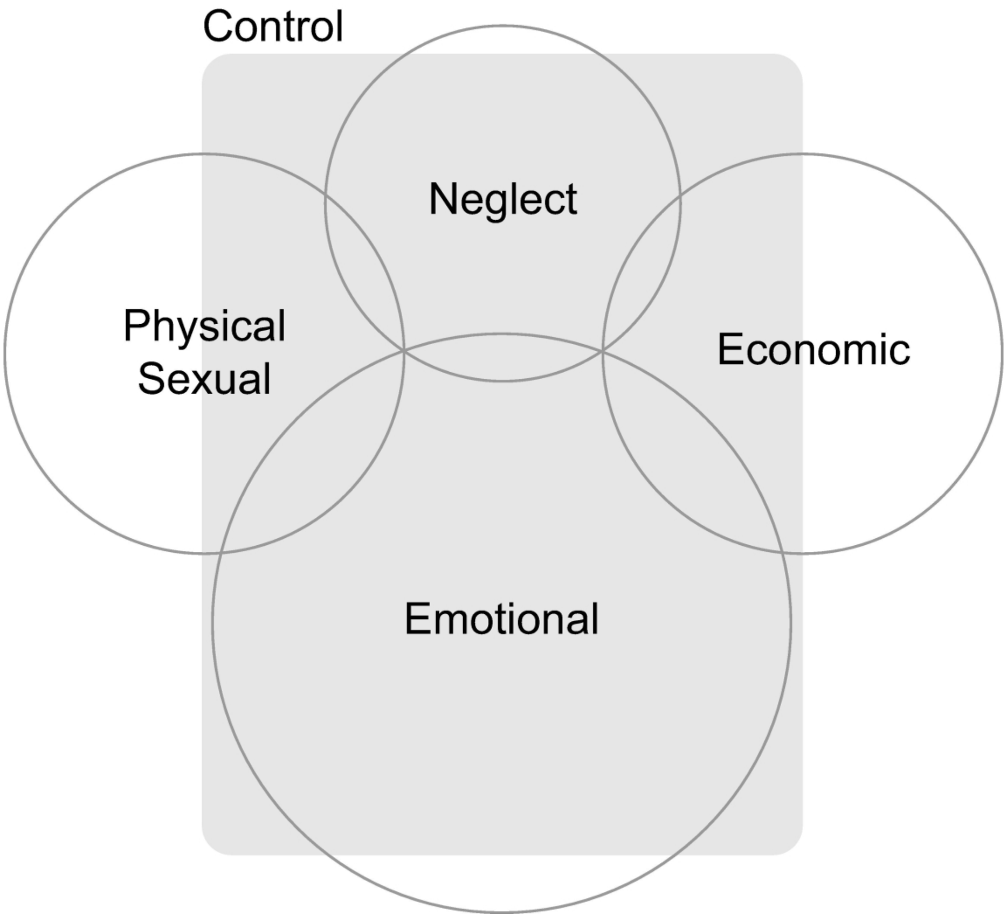

Figure 1. Forms of domestic violence against women

143x130mm (600 x 600 DPI)

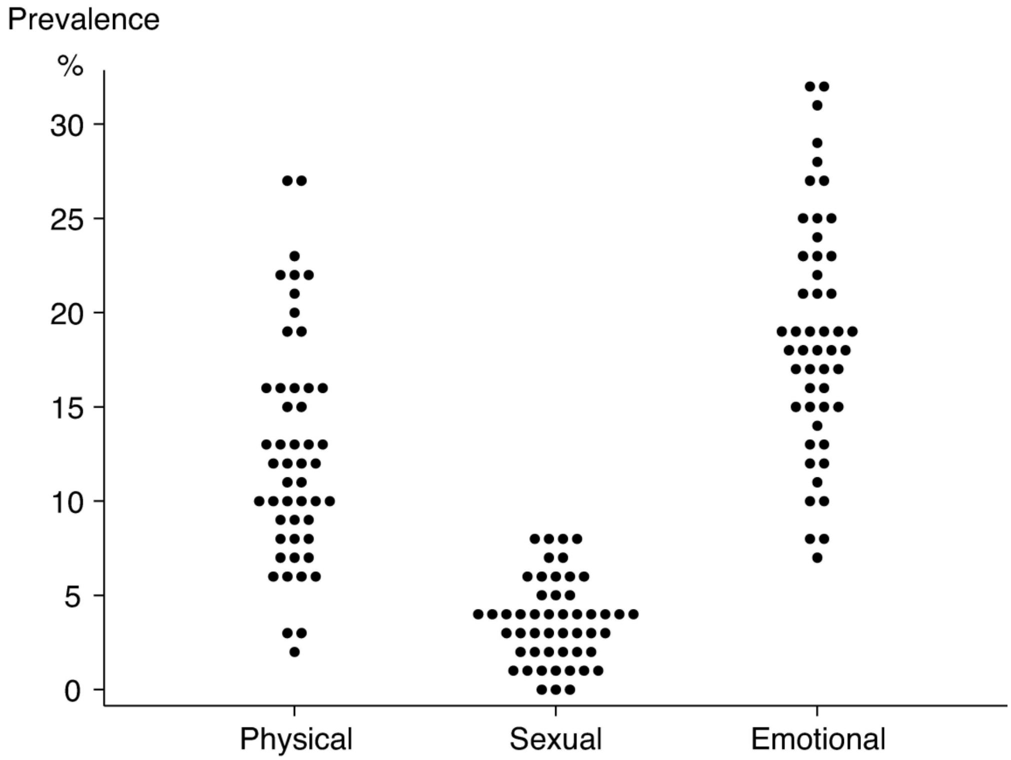

Figure 2. Prevalence of physical, sexual, and emotional violence against 5122 women aged 18-49 years in 50 informal settlement clusters in Mumbai

138x105mm (600 x 600 DPI)

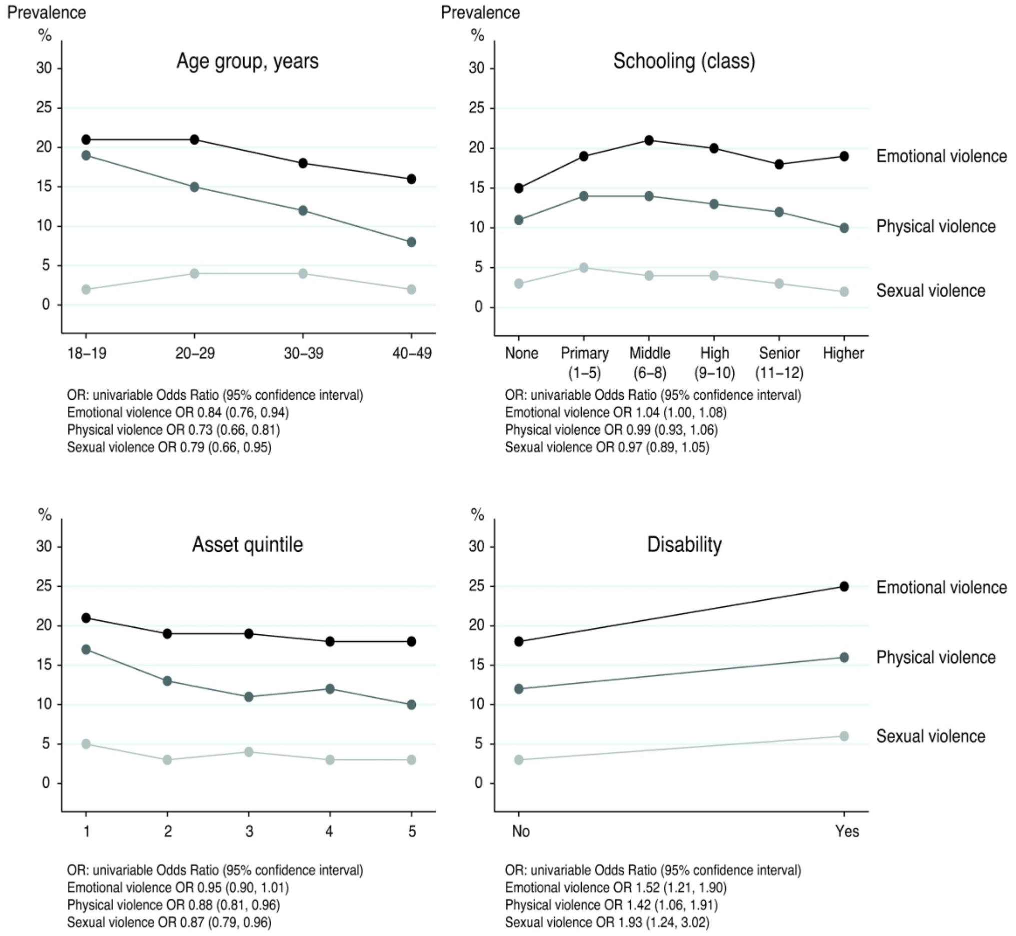

Figure 3. Prevalence of physical, sexual, and emotional violence against 5122 women aged 18-49 years, by age group, schooling, socioeconomic asset quintile, and disability

195x181mm (600 x 600 DPI)

STROBE 2007 (v4) Statement—Checklist of items that should be included in reports of *cross-sectional studies*

Section/Topic	Item #	Recommendation	Reported on page #
**Title and abstract**	1	(*a*) Indicate the study's design with a commonly used term in the title or the abstract	1
		(*b*) Provide in the abstract an informative and balanced summary of what was done and what was found	2
**Introduction**			
Background/rationale	2	Explain the scientific background and rationale for the investigation being reported	4-5
Objectives	3	State specific objectives, including any prespecified hypotheses	5
**Methods**			
Study design	4	Present key elements of study design early in the paper	5
Setting	5	Describe the setting, locations, and relevant dates, including periods of recruitment, exposure, follow-up, and data collection	5, 8
Participants	6	(*a*) Give the eligibility criteria, and the sources and methods of selection of participants	6
Variables	7	Clearly define all outcomes, exposures, predictors, potential confounders, and effect modifiers. Give diagnostic criteria, if applicable	7
Data sources/ measurement	8*	For each variable of interest, give sources of data and details of methods of assessment (measurement). Describe comparability of assessment methods if there is more than one group	7
Bias	9	Describe any efforts to address potential sources of bias	6
Study size	10	Explain how the study size was arrived at	5
Quantitative variables	11	Explain how quantitative variables were handled in the analyses. If applicable, describe which groupings were chosen and why	6-7
Statistical methods	12	(*a*) Describe all statistical methods, including those used to control for confounding	6-7
		(*b*) Describe any methods used to examine subgroups and interactions	7
		(*c*) Explain how missing data were addressed	7
		(*d*) If applicable, describe analytical methods taking account of sampling strategy	7
		(*e*) Describe any sensitivity analyses	N/A
**Results**			

For peer review only

Participants	13*	(a) Report numbers of individuals at each stage of study—eg numbers potentially eligible, examined for eligibility, confirmed eligible, included in the study, completing follow-up, and analysed	8
		(b) Give reasons for non-participation at each stage	8
		(c) Consider use of a flow diagram	N/A
Descriptive data	14*	(a) Give characteristics of study participants (eg demographic, clinical, social) and information on exposures and potential confounders	8 (Table 2)
		(b) Indicate number of participants with missing data for each variable of interest	8 (Table 2)
Outcome data	15*	Report numbers of outcome events or summary measures	8-10 (Tables 3, 4)
Main results	16	(*a*) Give unadjusted estimates and, if applicable, confounder-adjusted estimates and their precision (eg, 95% confidence interval). Make clear which confounders were adjusted for and why they were included	9
		(*b*) Report category boundaries when continuous variables were categorized	9
		(*c*) If relevant, consider translating estimates of relative risk into absolute risk for a meaningful time period	N/A
Other analyses	17	Report other analyses done—eg analyses of subgroups and interactions, and sensitivity analyses	9
**Discussion**			
Key results	18	Summarise key results with reference to study objectives	10
Limitations	19	Discuss limitations of the study, taking into account sources of potential bias or imprecision. Discuss both direction and magnitude of any potential bias	10
Interpretation	20	Give a cautious overall interpretation of results considering objectives, limitations, multiplicity of analyses, results from similar studies, and other relevant evidence	11
Generalisability	21	Discuss the generalisability (external validity) of the study results	11-12
**Other information**			
Funding	22	Give the source of funding and the role of the funders for the present study and, if applicable, for the original study on which the present article is based	13

*Give information separately for cases and controls in case-control studies and, if applicable, for exposed and unexposed groups in cohort and cross-sectional studies.

Note: An Explanation and Elaboration article discusses each checklist item and gives methodological background and published examples of transparent reporting. The STROBE checklist is best used in conjunction with this article (freely available on the Web sites of PLoS Medicine at http://www.plosmedicine.org/, Annals of Internal Medicine at http://www.annals.org/, and Epidemiology at http://www.epidem.com/). Information on the STROBE Initiative is available at www.strobe-statement.org.

For peer review only
